# Cognitive and behavioral radicalization: A systematic review of the putative risk and protective factors

**DOI:** 10.1002/cl2.1174

**Published:** 2021-07-20

**Authors:** Michael Wolfowicz, Yael Litmanovitz, David Weisburd, Badi Hasisi

**Affiliations:** ^1^ Faculty of Law, Institute of Criminology Hebrew University of Jerusalem Jerusalem Israel; ^2^ Department of Criminology, Law and Society George Mason University Fairfax Virginia USA

## Abstract

**Background:**

Two of the most central questions in radicalization research are, (1) why do some individuals radicalize when most of those from the same groups or exposed to similar conditions do not? and (2) why do radicalized individuals turn to radical violence while the majority remain inert? It has been suggested that the answer to both questions lie in the cumulative and interactive effects of a range of risk factors. While risk assessment and counter‐radicalization take a risk‐protective factor approach, there is widespread debate as to what these factors are and which are most important.

**Objectives:**

This review has two primary objectives.

1) To identify what the putative risk and protective factors for different radicalization outcomes are, without any predeterminations.

2) To synthesize the evidence and identify the relative magnitude of the effects of different factors.

The review's secondary objectives are to:

1) Identify consistencies in the estimates of factors across different radicalization outcomes.

2) Identify whether any significant heterogeneity exists within factors between (a) geographic regions, and (b) strains of radicalizing ideologies.

**Search Methods:**

Over 20 databases were searched for both published and gray literature. In order to provide a more comprehensive review, supplementary searches were conducted in two German and one Dutch database. Reference harvesting was conducted from previous reviews and contact was made with leading researchers to identify and acquire missing or unpublished studies.

**Selection Criteria:**

The review included observational studies assessing the outcomes of radical attitudes, intentions, and/or radical behaviors in OECD countries and which provided sufficient data to calculate effect sizes for individual‐level risk and protective factors.

**Data Collection and Analysis:**

One‐hundred and twenty‐seven studies, containing 206 samples met the inclusion criteria and provided 1302 effect sizes pertaining to over 100 different factors. Random effects meta‐analyses were carried out for each factor, and meta‐regression and moderator analysis were used to explore differences across studies.

**Results:**

Studies were primarily cross‐sectional, with samples representing 20 countries OECD countries. Most studies examined no specific radicalizing ideology, while others focussed on specific ideologies (e.g., Islamist, right‐wing, and left‐wing ideologies). The studies generally demonstrated low risk of bias and utilized validated or widely acceptable measures for both indicators and outcomes. With some exceptions, sociodemographic factors tend to have the smallest estimates, with larger estimates for experiential and attitudinal factors, followed by traditional criminogenic and psychological factors.

**Authors' Conclusions:**

While sociodemographic factors are the most commonly examined factors (selective availability), they also tend to have the smallest estimates. So too, attitudinal and even experiential factors, do not have effect sizes of the magnitude that could lead to significant reductions in risk through targeting by interventions. Conversely, traditional criminogenic factors, as well as psychological factors tend to display the largest estimates. These findings suggest the need to broaden the scope of factors considered in both risk assessment and intervention, and this review provides much needed evidence for guiding the selection of factors.

## 1 PLAIN LANGUAGE SUMMARY

### 1.1 Criminogenic factors are the most important risk factors for cognitive and behavioral radicalizationThe review in brief

This systematic review and meta‐analysis examines risk and protective factors for radicalization in democratic countries. The review includes 127 studies, half of which were published from 2018 to 2020. Among 101 risk and protective factors analysed, the most significant factors are those known to be related to criminal attitudes and behaviors, and social‐psychological factors.

#### What is this review about?

Radicalization entails the development of attitudes supportive of the use of violence in the name of a cause, and for a small number (<1%) of radicalized individuals, the carrying out of such violence.

Risk and protective factors, which increase or decrease the likelihood of these radicalization outcomes, are used in risk assessment and counter‐radicalization interventions. However, in practice, the selection of factors is often not evidence‐based. As a result, policies and practices are unlikely to be as effective as they could be, and can even increase stigmatization of certain communities, thereby increasing the risk of radicalization.

This systematic review supports the development of more evidence‐based approaches by identifying the relative magnitude of the effects for a large number of factors.

This Campbell systematic review examines putative risk and protective factors (correlates) of radical attitudes, intentions, and behaviours (including terrorism) in democratic countries. The review examines 101 factors, derived from over 1300 effect sizes extracted from 127 studies.

### 1.2 What are the putative risk and protective factors for radicalization and what are the relative magnitudes of their effects?

The review identifies 101 individual‐level factors for radical attitudes, 45 for radical intentions, and 33 for radical behaviors. The factors can be grouped into five domains:

1) Sociodemographic and background factors,
2) Psychological and personality trait factors,
3) Attitudinal and subjective belief related factors,
4) Experiential factors, and
5) Criminogenic and criminotrophic, factors known for fostering or protecting against a range of deviant outcomes, both cognitive and behavioral.

**Figure 1:**
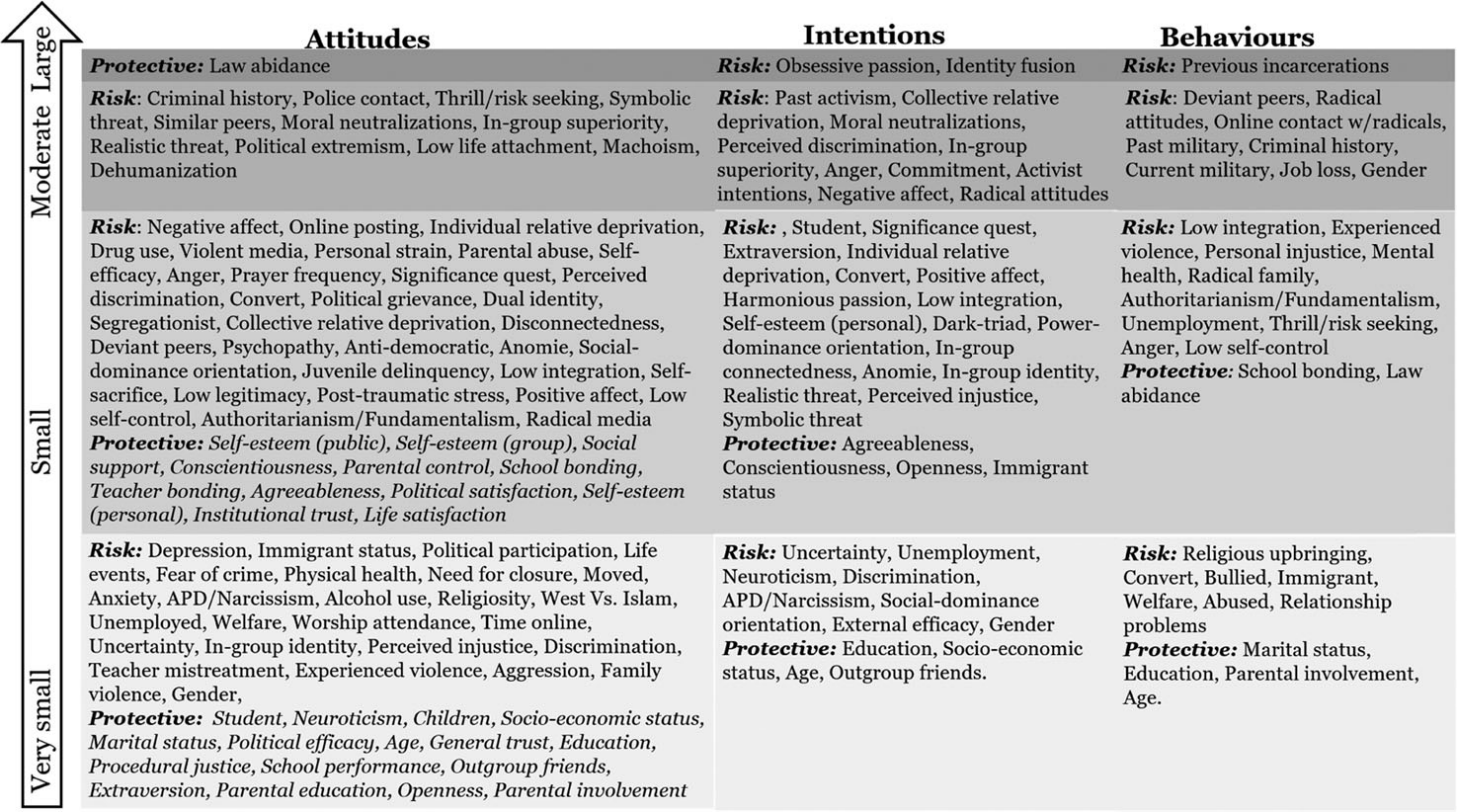
Distribution of factors identified across outcomes according effect size category

A small number of factors have moderate (*r* = .30–.49), or large estimates (*r* = .50–.63), while the majority of the factors have small‐very small relationships with radicalization outcomes. Across all outcomes, key sociodemographic factors tend to have the smallest estimates, with increasingly larger estimates for experiential and attitudinal factors, and criminogenic and psychological factors (see Figure 1).

### 1.3 Differences in estimates by geographic region and ideology

For the outcome of radical attitudes, the estimate for Moral neutralizations was largest for US‐based samples. For radical intentions, the estimates for Personal self‐esteem and Commitment to a cause were largest in Europe‐based samples. With regard to radical behaviors, the estimate for Unemployment was significantly larger for Europe‐based samples.

**Figure 2:**
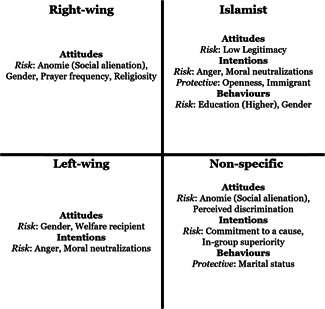
Factors with significant between‐ideology differences

For the most part, there were no significant differences found in the size of the estimates for factors across ideological strains. However, differences were found for seven factors for radical attitudes, six for radical intentions, and three for radical behaviors. The findings are summarized in the below figure which highlights for which ideology significantly larger estimates were found for a given factor (see Figure 2).

#### What do the findings of this review mean?

Some of the factors most central to risk assessment and counter‐radicalization interventions actually have relatively small relationships with radicalization outcomes. Conversely, factors known to be associated with ordinary criminal outcomes have the largest relationships. These findings suggest the need for moving towards weighted risk assessment instruments, and alternative interventions.

Additionally, findings of differences in the magnitude of the effects for different factors according to regional context suggest that risk assessment and interventions may be tailored to local contexts.

#### How up‐to‐date is this review?

The review authors searched for studies up to March 2020.

## 2 BACKGROUND

### 2.1 The Issue

Over the last three decades there have been numerous shifts in the popular paradigms through which we understand terrorism and conceive of ways to counter it. The different perspectives have included a focus on profiles, root causes, and pathways (Horgan, 2008). But in more recent years, a new perspective has developed from changes to the landscape of terrorism threats facing democratic countries, such as the rise of home‐grown and lone‐actor terrorism, and a deeper understanding of the difficulties in combatting such threats. It is from this point of departure that researchers and policy‐makers have increasingly come to adopt the now dominant “radicalization” perspective (Neumann & Kleinmann, 2013).

In its 2005 definition, the EU defines radicalization as “the phenomenon of people embracing opinions, views and ideas which *could* [sic.] lead to acts of terrorism” (European Comission, 2005). The EU's definition addresses two key issues that have been raised in the literature. First, it demonstrates that there are two outcomes of radicalization. The first are certain opinions, views, and ideas, and the second is terrorism, representing cognitive and behavioral outcomes respectively. Second, in making these distinctions, this definition emphasizes that while certain opinions, views and ideas can underpin or lead to terrorism, it is not necessarily the case that they will; only that they could. These distinctions have been adopted by most researchers, who have consistently and repeatedly emphasized the need to differentiate the cognitive from the behavioral outcomes of radicalization. While cognitive radicalization, is likely to underpin almost all acts of behavioral radicalization, such as terrorism, less than 1% of those who hold radical beliefs, ideas and opinions will ever engage in acts of radical violence. That is, the overwhelming majority of cognitive radicals will forever remain inert (McCauley & Moskalenko, 2017). Nevertheless, the evidence overwhelmingly indicates a strong inter‐correlation between radical attitudes and intentions (e.g., Feddes et al., 2015; Schbley, 2004), and between these cognitive outcomes with radical behaviors (e.g., Baier et al., 2016; Bélanger et al., 2014).

While the EU's definition is useful for understanding what radicalization is, like many others it fails to specify what ideas or types of beliefs may constitute the cognitive elements of radicalization (Schmid, 2013). While many different proxies have been used in research, as with any attempt to assess attitudinal or cognitive antecedents of a given behavior, the attitudinal measures need to have a high degree of specificity with reference to the behavioral outcome of interest (Ajzen & Fishbein, 1980; Fishbein & Ajzen, 1975). In the case of radicalization, the primary behavioral outcome of interest is terrorism. As such, attitudinal measures of radicalization that include specific appraisals of terrorism would be considered to have a high level of specificity (Schmid, 2017).

**Figure 3:**
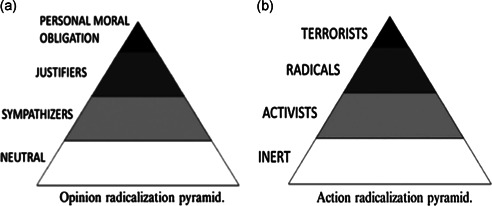
McCauley and Moskalenko's (2014) Two‐Pyramid Model

The need for measures of cognitive radicalization to demonstrate a high level of specificity to the potential behavioral outcomes of radicalization underpins McCauley and Moskalenko's (2017) outcome‐based typology model, known as the Two‐Pyramid Model (TPM) of radicalization. Like other models, the TPM provides a clear distinction between the cognitive and behavioral outcomes of radicalization, without distinction between motivating ideologies. Unlike some models which are ideology‐specific (e.g., Silber & Bhatt, 2007) the TPM is a general model. Indeed, while differences certainly exist, the degree of overlap in the factors that predict radicalization to different ideologies is larger (e.g., Chermak & Gruenewald, 2015). The TPM is even more general in that, unlike other models, it does not specify any paths between the outcomes. Rather its focus is on the typologies, determining when a specific opinion or behavior can be defined as being 'radical', and in which classification the specific opinion or behavior falls. According to the TPM, every individual “radical” exists at some level on each of the pyramids simultaneously at any given point in time. The narrowing shape of the pyramid indicates that a smaller segment population falls into the category. On the cognitive radicalization pyramid there are those who sympathize and empathize with, or outright justify subterroristic radical violence or terrorism. Subterroristic radical violence includes acts of violence against persons and property that is usually nonlethal and falls short of the law's definitions of terrorism. Terrorism includes serious acts of violence against persons, usually intended to inflict injury or death, or attempts to disrupt or destroy critical infrastructure in the name of a cause or ideology. A small percentage of justifiers believe they have a personal moral obligation to carry out actions in defence of their cause. This may be expressed as intentions or a willingness to engage in a variety of legal, nonviolent actions (activism), or illegal and violent actions (radicalism/terrorism) (Leuprecht et al., 2010). However, even among those individuals from this category, the majority will remain forever inert (Figure 3).

Given what is known about these relationships, researchers have been primarily interested in identifying what leads to the shift from cognitive to behavioral radicalization. The TPM does not, however, provide a set of mechanisms or explanation as to why some individuals develop cognitive radicalization while other similar individuals do not. Similarly, it does not provide a set of mechanisms or explanations as to why some cognitive radicals will engage in radical behaviors while the majority will not. The developers of the TPM have suggested a number of possible risk and protective factors, including personal or group grievance and thrill‐seeking, or parental bonds (Leuprecht et al., 2010; McCauley & Moskalenko, 2008; Moskalenko & McCauley, 2011). These, as well as a host of other factors have been noted in previous systematic reviews. However, these reviews, which primarily use narrative or 'vote counting methods' have found that the evidence for most factors is quite mixed and often contradictory (Wolfowicz et al., 2020).

In the absence of systematic assessment, it is difficult to reach a consensus as to which factors should be considered risk and protective for radicalization (Allan et al., 2015; Bondokji et al., 2017 et al., 2017; Victoroff, 2005). Moreover, this gap in the literature means that the relative magnitude—or importance—of the different factors remains unknown (Crenshaw, 2007; Gill, 2016; Hafez & Mullins, 2015; Haggerty & Bucerius, 2018; Staring, 2014). These gaps have serious implications for counter‐radicalization policy and practice. First, with regard to risk assessment tools, the inability to establish evidence‐based weights for different items means that they overwhelmingly take a nominal scaling approach. This approach can lead to a high false‐positive rate for at‐risk classifications (Klausen et al., 2016). This can lead to multiple issues, such as a false impression about the extent of radicalization in a population, and the potential stigmatization of certain communities (Klausen et al., 2016; Lösel et al., 2018). This can also lead to an erosion of legitimacy for a country's counter‐radicalization approach more generally (van de Weert & Eijkman, 2020). Perhaps even more so with counter and de‐radicalization programs, “poorly designed programs are not only a waste of resources but also may increase the risk of violence” (Koehler, 2019, p. 59). There is therefore a need to systematically categorize risk and protective factors for radicalization and to identify the relative magnitude of their effects.

### 2.2 Risk and protective factors

#### 2.2.1 The risk‐protective factor approach

In line with what is known about radicalization, the criminological risk‐protective factor framework maintains that no specific or single risk factor can or will cause violent behaviors. Rather, it is the accumulative and interactive weight of present risk factors, either in the absence or outweighing of protective factors, that increases or decreases the likelihood or risk of offending (Farrington et al., 2016). One of the most significant risk factors for criminal behaviors is criminal attitudes (or cognitions). However, only a small number of individuals who hold criminal attitudes will go on to engage in criminal behaviors (Kim & Hunter, 1993; Pogarsky, 2004). Nevertheless, the majority of those who engage in criminal behaviors are likely to hold criminal attitudes. In recognition of this fact, while most of the research on risk factors has focused on behavioral outcomes, scholars have increasingly been interested in examining risk and protective factors for antecedent cognitive outcomes, including both criminal attitudes and intentions (e.g., Mazerolle & Piquero, 1997; Walters, 2017; Willits, 2015, 2019).

Many of the same risk factors and protective factors tend to predict each of the outcomes of criminal attitudes, intentions, and behaviors. For example, anger and exposure to violence have been found to predict criminal attitudes (Baron et al., 2001), and intentions (e.g., Mazerolle & Piquero, 1997). Similarly, deviant peers (differential associations) and low self‐control have been found to predict criminal intentions (Skrzypiec, 2017). Willits (2015) found that anger, low self‐control, prior violence and peer violence were all risk factors for violent intentions. Each of these factors figure prominently among risk factors for criminal offending behaviors as well (Hawkins et al., 2000; Walters, 2015).

The differential balance of risk and protective factors that present themselves in individuals, may serve to explain why only a small number of those who hold criminal attitudes or intentions will engage in criminal or criminal analogous behaviors, and most would not (Farrington et al., 2016; Folk et al., 2018; Walters, 2018). In other words, risk and protective factors mediate the relationship between criminal cognitions and behaviors. Some traditional criminogenic factors have been found to be moderators for this relationship, including; thrill‐seeking and low self‐control (Brezina, 2010; Bulten et al., 2009; Skrzypie, 2017; Walters, 2016), deviant peers and criminal history (Boduszek et al., 2011, 2013).

#### 2.2.2 Risk and protective factors for radicalization

Understandings about how risk and protective factors affect criminal attitudes and behaviors, and the relationship between these distinct but interrelated outcomes, have important implications for understanding the distinctiveness between the cognitive and behavioral outcomes of radicalization, but also the nature of their relationship (Hafez & Mullins, 2015; Khalil, 2014; Neumann, 2013; Carpenter et al., 2009). The literature indicates that there are significant overlaps between criminals and terrorists, who also tend to come from similar segments of the population (Clarke & Newman, 2006). Additionally, criminality is a common feature of terrorists' backgrounds. As such, traditional criminogenic and criminotrophic factors may have relevance for radical attitudes, intentions and behaviors as well (Lösel et al., 2018; Wolfowicz et al., 2020). However, there are also a number of theoretical perspectives that emphasize different factors that are thought to be more specific to radicalization. And while differences certainly exist, it is believed that risk and protective factors for radicalization are relevant for a spectrum of radicalizing ideologies, including Left‐wing, Right‐wing, and religious ideologies. While it would be both impossible and undesirable to attempt to review all of the factors noted in the literature (Monahan, 2012, 2016), below are some of the most widely discussed factors pertaining to the domains of sociodemographics, attitudinal and experiential factors, psychological/personality trait related factors, and criminogenic factors.

#### 2.2.3 Sociodemographic factors

Almost all perspectives on radicalization place some degree of emphasis on sociodemographic background characteristics. While there are certainly exceptions, young, single males are seen as the most susceptible to radicalization and indeed make up the largest number of radical offenders (Meloy & Gill, 2016). However, there are mixed perspectives concerning other factors. For example, poverty and low socioeconomic status have traditionally been viewed as risk factors for radicalization (Atwood, 2003). However, the evidence is actually quite mixed (Victoroff, 2005). While some studies have found a high prevalence of poor socioeconomic standing among terrorists in both the US and Europe (Bakker, 2011; Handler, 1990; Ljujic et al., 2020), other have found that many terrorist offenders come from the middle and even upper‐middle classes (Berrebi, 2007; Russell & Miller, 1977; Sageman, 2008). Additionally, those of the poorest settings appear the least likely to be involved in terrorism (Berrebi, 2007; Lee, 2011). Similarly, while some studies examining radical attitudes have also found correlations between low socioeconomic status and support or justification of terrorism (e.g., Pedersen et al., 2017), others have found the relationship to point in the opposite direction (e.g., Berger, 2016; Bhui et al., 2016).

There is also mixed evidence with regard to other sociodemographic factors such as education and employment (Victoroff, 2005). While many have assumed that low education breeds radicalization, studies have found that terrorist offenders in the West often have some degree of post‐secondary education, or were even current students at the time of their attacks (Carlton, 1979; Gambetta & Hertog, 2017; Russell & Miller, 1977; Weinberg & Eubank, 1988). Similarly, while some studies have found an association between lower education and support for terrorism, other have found that the relationship exists in the opposite direction, and that full‐time students are more likely to express these types of attitudes (Bhui et al., 2014a; Krueger & Maleckova, 2002; Schbley, 2003). Indeed, the university has long been considered as a 'hot‐spot' for radicalization going back to at least the 1960's (Brown & Saeed, 2015; Glees & Pope, 2005; Saeed & Johnson, 2016). According to the NYPD's original four‐stage model of radicalization, those with higher education are considered at higher risk, and this factor is most relevant to the preradicalization stage (Silber & Bhatt, 2007).

Unemployment has traditionally been viewed as a key risk factor for radicalization (Sageman, 2004), and has been found to be more prevalent among violent radicals than the general population (Altunbas & Thornton, 2011; Ljujic et al., 2020) and nonviolent radicals (LaFree et al., 2018). However, Krueger (2008) found no statistically significant differences between a samples of terrorists and the general population in being unemployed (and not currently in education). In a study comparing Palestinian terrorists with the general population, it was found that 90% of terrorists were in full‐time employment, compared to only 60% of the general population (Berrebi, 2007). Similarly, with regard to radical attitudes, while some studies indicate a positive correlation with employment (e.g., Bhui et al., 2016), others have found a negative correlation (e.g., Acevedo & Chaudhary, 2015).

#### 2.2.4 Experiential and attitudinal factors

##### Strain theory

Sociodemographic factors such as socioeconomic status and (un)employment can also operate as second‐order factors (Boehnke et al., 1998). For example, poor socioeconomic outcomes can also increase the likelihood of other factors that are prominent in radicalization models, such as grievance and personal injustice (Borum, 2003, 2011). According to Agnew's General Strain Theory as applied to the context of radicalization and terrorism (Agnew, 2010, 2016), individual level stressors, such as worries about money, may push individuals into the arms of extremists. Agnew (2016) explains that even anticipated strains can be risk factors for radicalization and terrorism. This is perhaps linked to the perceptions that many members of minority groups are discriminated against in the job market, and that while employed, they are underemployed. It can also relate to uncertainties about the potential for future strains, both at the individual level (e.g., economic) and group level (e.g., discrimination).

##### Relative deprivation

Similar to strain theory, the relative deprivation perspective holds that actual material resources available to an individual may be less important than perceptions. However, in the case of relative deprivation theory, the perceptions pertain to evaluations against other individuals, groups, or alternative possibilities (Davis & Cragin, 2009; Gurr, 1970; King & Taylor, 2011). As such, there are essentially three different types of relative deprivation. The first is when an individual evaluates their group's situation, status, and standing as subjectively and relatively worse than those of other groups, presumably as a result of some form of discrimination against the group. The second type comes in the form of a sense that one's group or its members lack those things that they truly desire or believe they deserve, even if their material needs are being met; the desired outcomes may be nonmaterial, such as political power. The third possible source of relative deprivation is the vicarious identification with an objectively deprived group. In this case, the individual or group identifying with the deprived group may not necessarily be collectively or individually deprived (McCauley, 2002; Moghaddam, 2005). While originally used to explain group‐based political violence (Pasquino & Della Porta, 1986; Wilkinson, 1982), the individual expression of both individual and collective variants of relative deprivation may account for the radicalization of relatively well off and educated individuals' participation in terrorism (Borum, 2004; Campana & Lapointe, 2012; Gambetta & Hertog, 2009).

The three types of relative deprivation demonstrate considerable overlap with a number of other perspectives. For example, Anomie theory, which points to a lack or breakdown of norms in an individual or group as a result of societal alienation, sometimes resulting from clashes between cultures (Coolsaet et al., 2019). Similarly, according Agnew's (2016) collective strain theory, groups and their members may resort to violence under conditions of strain and stress, especially when prolonged. A number of studies have found that collective and individual measures of relative deprivation correlate with radical attitudes (e.g., Doosje et al., 2012, 2013). However, as with other subjective, attitudinal factors, it is difficult to assess how such relative deprivation affects radical behaviors directly (Dartnell, 1995). But relative deprivation also has implications for other factors, such as identity and belonging, community, trust, and discrimination. The vicarious forms of relative deprivation can increase the salience of an individual's actual situation, or increase the degree to which they view themselves as being relatively deprived (McCauley & Moskalenko, 2008; Victoroff, 2005). This means that relative deprivation may also increase the degree to which an individual's identity becomes fused with that of a particular group, and detached from others (Abbas, 2005, 2007; Spalek & Imtoual, 2007; Spalek, 2007).

##### Integration, trust, and discrimination

Poor social integration alongside a lack of institutional trust are often the outcome of both collective and individual experiences of discrimination (Burt & Simons, 2015; Simons et al., 2003). Victimization and discrimination can lead to feelings such as anger and a desire for revenge, and accordingly increase the likelihood of radicalization (Bjørgo, 2005; Dandurand, 2014). Similar to relative deprivation perspectives, victimization or perceptions of discrimination, racism, and injustices at both the individual and group level can be risk factors for radicalization (Berger, 2016; Brettfeld & Wetzels, 2007; Bhui et al., 2014b; McCauley, 2012; Pauwels & de Waele, 2014; Simons et al., 2003; de Waele & Pauwels, 2014). When individuals view a lack of governmental action being taken to combat discrimination, it can erode existing levels of integration and institutional trust. All of these factors are believed to greatly increase the risk of radicalization (King & Taylor, 2011; Wilner & Dubouloz, 2010). As Pressman (2006, p. 3) writes: “it is generally accepted by experts that failed integration, frustration within the host society, identity issues, and conflicting values with western democracies are contributing factors to radicalization.” Indeed, there is evidence that all of these factors are positively related to increased support for terrorism (Bhui et al, 2014a; Brettfeld & Wetzels, 2007) and radical behaviors (Pauwels & de Waele, 2014; Pauwels & Schils, 2016). In two separate experiments, social exclusion and ostracism increased the likelihood of a willingness to join, and act violently on behalf of a radical group (Pfundmair & Wetherell, 2019). Other studies have found that higher levels of general and social trust may act as protective factors against radicalization (Bhui et al., 2014a; Nivette et al., 2017; Szlachter et al., 2012). Ellis and Abdi's (2017) research among Somalian immigrants in North America led them to conclude that strengthening community integration and cohesion can lead to resilience against radicalization.

According to King and Taylor (2011), issues pertaining to integration, trust, and discrimination can lead to an identity crisis in which individuals may become torn between the degree to which they identify with their in‐group versus their membership in the state or society in which they reside. Identity crises of this nature may spur a quest for identity (See further discussion below). In some cases, this quest may lead to a renewed interest in religion (Ghosh et al., 2013). This quest may bring an individual into contact with new people, including radicalizing agents (King & Taylor, 2011; Sageman, 2008). It is important to note that this process has also been noted for right‐wing extremism (Bjørgo, 1997).

#### 2.2.5 Psychological/personality trait factors

##### Quest for significance

A quest for identity, or significance, is widely regarded as being a source of risk for both religious and nonreligious variants of radicalization (Borum, 2014). Poor social integration, or perceived exclusion can lead to a loss of significance (Bäck et al., 2018). In seeking to restore significance, individuals from marginalized groups may be drawn to radical identities, groups, and ideologies (Silber & Bhatt, 2007). There is some good evidence that the loss of significance, stemming from a range off experiences, is quite prevalent among radical offenders (Jasko et al., 2016). As identified by a recent group of experiments, which included samples of incarcerated terrorists in Sri Lanka and the Philippines, feelings of insignificance increased uncertainty and a need for closure, which moderated the effects on radicalization. As the researchers note, a loss of significance can be the result of both individual and group‐based discrimination and humiliation (Kruglanski et al., 2016; Webber et al., 2018). Related to the quest for significance and identity is a quest for status (Venhaus, 2010). Some believe that many radicals may turn to terrorism in order to fulfill their psychological needs for status, similar to the criminogenic needs of many ordinary criminals (Clarke & Newman, 2006; Lloyd & Kleinot, 2017). Others have classified status seeking as a key 'pull factor' for radicalization (Bartlett & Miller, 2012).

##### Thrill‐seeking

Another type of “quest” is found in the form of thrill‐seeking. Venhaus (2010) defined the thrill‐seeker radical as one who becomes attracted to radicalization because it offers the potential to provide them with excitement, adventure, or glory. Like the status seeker, thrill‐seeking also overlaps with risk factors known for ordinary crime. According to Gottfredson and Hirschi's (1990) Self‐Control Theory (SCT), certain innate psychological characteristics, namely self‐control and thrill‐seeking behaviors, determine whether or not someone will engage in illegal behaviors. While there are both attitudinal and behavioral measures of self‐control, both in self‐control theory and the Theory of Planned Behavior (TPB), there is evidence that both measures have positive correlations with both radical attitudes and behaviors (Pauwels & Schils, 2016). Biographical accounts offered by former terrorists provide indications that thrill‐seeking and risk‐taking were among the factors that attracted them to radical groups (Silke, 2008).

Regarding low self‐control however, early considerations about its applicability to terrorism were dismissive (Hoffman, 1998). Even Hirschi and Gottfredson (2001) themselves sought to minimize the theory's application to terrorism, which they considered to be fundamentally different than ordinary crime. But despite these rejections, a number of leading researchers believed that personality traits such as low self‐control and thrill‐seeking/risk taking may be risk factors for radicalization (Borum, 2011; Silke, 2008). Indeed, recent studies have found positive correlations between low self‐control and radicalization (Baier et al., 2016; Koomen & Van Der Pligt, 2015; Nivette et al., 2017; Pauwels & de Waele, 2014; Pauwels & Schils, 2016).

##### Other psychological and personality related factors

Early terrorism and radicalization research were guided by popular notions that terrorists were 'mad'. However, as early as the late 1980's researchers concluded that this was not the case, with evidence pointing to an overwhelming tendency towards normalcy among terrorism offenders (Crenshaw, 1981; Heskin, 1984). However, in more recent years the debate concerning psychological health and characteristics has been revived (Corner & Gill, 2015, 2017; Corner et al., 2016). Research on homegrown terrorists, lone wolves, and foreign fighters have found a high prevalence of psychological‐related issues among offenders. For example, among 140 failed foreign fighters from the Netherlands Weenink (2015) found that 60% had evidence of psychological problems. In interviews with 44 former white supremacists, Simi et al. (2016) found that 41% suffered from some form of mental health issues (Simi et al., 2016). However, a series of studies from a group of psychiatrists in the UK have found either nonsignificant or negative effects of mental health issues such as depression and anxiety on support and justification of terrorism (Bhui et al., 2014a, 2014b; Coid et al., 2016).

These discrepancies may relate to issues of measurement or classification. For example, in Weenink's (2015) study, the majority of these issues related to problem behaviors (46%) and serious problem behaviors (14%), with only 6% having a diagnosed personality disorder or mental illness. Coid et al. (2016) found that while depression and anxiety were not correlated with radical attitudes, clinical antisocial personality disorder was. As such, while clinical mental illness may not be especially important risk factors for radicalization, other types of psychological or personality traits may be (Dalgaard‐Nielsen, 2008). Indeed, in Simi et al.'s (2016) study, 73% of the sample reported having a history of a range of conduct problems. European studies have also found that conduct problems have significant correlations with radical attitudes (Baier et al., 2016; Pederson et al., 2017). Unfortunately, radicalization research has been conspicuously averse to examining a broader range of psychological and personality traits as risk factors for radicalization (Stern, 2016).

#### 2.2.6 Criminogenic factors

##### Radical peers and networks

Virtually all theoretical models of radicalization place some degree of emphasis on the role of peers, networks, and communities. The potential negative effects of deviant peers who support, or who are involved in criminal analogous behaviors form the basis of social learning and social control perspectives, as well as related theories such as techniques of neutralization and subculture theories. All of these theories have previously been proposed as possible frameworks through which to analyze and understand the effects of peers and networks on radicalization and recruitment to terrorism (e,g, Holt et al., 2018; Pisoiu, 2015). Studies have found that radicals are likely to have highly similar associations (Wojcieszak, 2010). Compared with nonviolent radicals, violent radicals are more likely to have radical peers or to have been part of a radical network (LaFree et al., 2018). In experimental settings, socialization with peers was found to have a causal effect on choosing radical and nonradical political solutions. Importantly, the choice for radical solutions was found to be dependent on participants' preexisting attitudes towards radical actions as being legitimate (Thomas et al., 2014).

The effects of deviant peers, or differential associations need not be restricted to in‐person associations, or even associations with persons per se. Social learning theorists have pointed out that pieces of media content are also forms of differential associations and can be sources of social learning (Akins & Winfree Jr., 2016). While Sageman's (2008) prediction that online associations would replace offline associations has not fully come to fruition (von Behr et al., 2013), the role of online media as a risk factor for radicalization has increased in recent years. Recent studies have shown that both jointly and severely, passive exposure to radical content online, and active engagement with other radicals over the internet, have a positive correlation with radical attitudes and behaviors (Frissen, 2019; Pauwels & Schils, 2016).

### 2.3 How risk and protective factors might work

As noted above, almost all of the major perspectives of radicalization hold that cognitive elements of radicalization, namely radical attitudes and intentions, are among the most important antecedent to radical behaviors. One of the unique features of the Two‐Pyramid model (TPM) is that it does not specify which types of factors may lead to the crossover from those with radical attitudes and who are inert, to becoming behaviorally radicalized. While the authors of the TPM have elsewhere suggested a number of different factors, as they acknowledge, these are not the only factors. What the TPM does emphasize however is that it is radical attitudes or intentions that tend to underpin the likelihood of radical behaviors. As noted above, while there are some cases in which those who engage in radical behaviors may not have been among the most radicalized cognitively, most behavioral radicals were first cognitively radicalized before engaging in radical behaviors.

These basics of the TPM are heavily rooted in behavioral and social psychology, namely the Theory of Reasoned Action (TRA), and its successor the Theory of Planned Behavior (TPB). The developers of the TPM reference these theories in the development of their outcomes and in explaining the relationships between them, as well as in the development of their own Activism‐Radicalism‐Intensions‐Scales (ARIS) to measure radical intentions (Moskalenko & McCauley, 2009). According to both the TRA and TPB, while attitudes and intentions towards a given behavior rarely predict engagement in the behavior, they are still one of the best predictors of engagement in the behavior. In order for an individual to engage in the behavior, they almost always first hold positive predispositions towards the behavior. As discussed above, attitudes and intentions have the greatest predictive quality for behaviors when the attitudinal and intentional measures have a high degree of specificity with respect to the behavioral outcome (Ajzen & Fishbein, 1980; Fishbein & Ajzen, 1975).

To a large degree, the TPB was developed in order to account for the limitations of the TRA in explaining behavioral outcomes that could be seen as being somewhat beyond the individual's control. In order to account for this, the TPB improved on the TRA by adding two sets of “risk factors,” namely normative values and beliefs, and perceived and actual self‐control. In the context of deviant or criminal behaviors, both of these factors are known criminogenic factors, and are central to control theories (social control and self‐control theories respectively). Other known criminogenic factors, such as moral neutralizations and personal strains may be viewed as falling within the categories of normative values and perceived control respectively (Skrzypiec, 2017). While factors associated with these prescribed domains may explain a large proportion of the variance in the attitude‐intention‐behavior continuity for a wide range of behaviors, Beck and Ajzen (1991) assert that in the context of offending behaviors, it is necessary to identify and include additional antecedent factors. This position was already expressed by Tuck and Riley (1986), who argued that all known risk factors for criminal outcomes operate through the attitudes‐intentions‐behaviors continuum. With regard to radicalization, there is some evidence to support this logic model. In studies of U.S. based violent and nonviolent radical offenders, group based grievances (a known risk factor) significantly predicted radical attitudes, which in turn predicted increased odds of being a violent over a nonviolent radical (Grace, 2018; Jensen et al., 2018).

A recent collaboration between one of the authors of the TRA and TPB (Icek Ajzen) with leading radicalization researcher Arie Kruglanski (who is also responsible for the development of the Quest for Significance Theory (QST) of radicalization) has led to the integration of TPB with Goal Systems Theory (GST). The authors emphasize that all behavioral decisions are made based on the weighing of alternative options, and how these different options are perceived to achieve the individual's goals. Differential behavioral outcomes are the result of differences in individual motivations, goals, and contemporary assessments of which behavioral option has the greatest subjective values. These differences can serve to explain why for most cognitive radicals the behavioral choice is to remain inert (Ajzen & Kruglanski, 2019). At the cognitive level, for some individuals, the adoption of radical attitudes may help them achieve goals of belonging, or conforming to the norms of their group, thereby attaining the significance that they seek. At the behavioral level, many radicals may view nonviolence as the best way to achieve their goals, or they may view that there are multiple means to achieve their goals. However, some may view radical behaviors such as terrorism as the only possible means for achieving their goals (Kruglanski et al., 2015).

**Figure 4:**
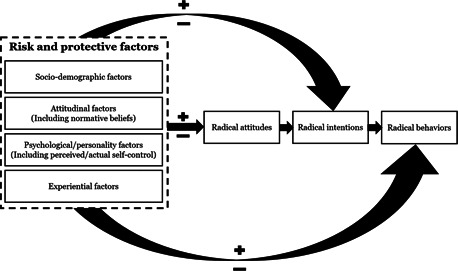
Logic model abstracted from the Theory of Planned Behavior

The logic model depicted below (Figure 4) is similar to what has been described and demonstrated by a number of scholars with regard to a range of criminal and criminal analogous outcomes. It demonstrates how the cumulative weight of risk and protective factors increases or decreases the likelihood of radical attitudes, which in turn predict radical intentions, and intentions predict behaviors. As noted above, risk and protective factors can also have interactive effects. For example, as described above, experiences of discrimination can influence factors such as integration, grievance or anger. Given the wide‐range of possible factors and interactions the below model does not depict these but does assume that they occur. In line with the TPM, there can also be direct effects of risk and protective factors on the outcomes of radical intentions and behaviors, both in the presence and absence of strong radical attitudes. Moreover, unlike some applications of the TPB that consider attitudes or intentions as proxies for behavior, this logic model is in line with the TPM that views attitudinal, intentional, and behavioral outcomes as distinct, albeit inter‐related outcomes.

Another important aspect of this model is that it can take into consideration that involvement in radical behaviors can serve as a risk factor for radical attitudes, as well as future radical behaviors. As per the TPM, radical attitudes generally (although not in all cases0 precede radical behaviors (Moskalenko & McCauley, 2020). Additionally, it is important to bear in mind that the development of radical attitudes can be something quite instantaneous or something that takes place over a long period of time. So too, there is a wide distribution of time from radical attitudes to behaviors, and the timing between the development or experiencing of risk factors can also impact the different outcomes at any given point in time (Klausen et al., 2016, 2020) (Figure 4).

This logic model serves not only to help contextualize the role of risk and protective factors in increasing or decreasing the likelihood of differential radicalization outcomes, or movements between them. It also provides a model for integrating the risk‐protective framework into counter and de‐radicalization interventions. As noted above, most initiatives of this variety attempt to reduce the likelihood of radical behaviors by tackling radical attitudes, and they go about doing this through the targeting of underlying risk and protective factors. While a number of scholars question the potential for actual de‐radicalization (the changing of radical beliefs), there is evidence to suggest that by tackling underling risk and protective factors it is possible to change radical attitudes, and that changes in these attitudes lead to changes in behavioral intentions, and actual behaviors (e.g Kruglanski et al., 2014).

### 2.4 Why it is important to do the review

#### 2.4.1 The current state of the literature

Despite the growth of radicalization and terrorism research in recent years, empirical studies still only account for a small percentage of the knowledge (Christmann, 2012; Schuurman, 2020). There is therefore little concrete information upon which policies and interventions can be developed, and they are therefore unlikely to have the desired impact (Davis, 2014). While such policies and strategies aim to tackle risk factors, there is often mixed and contradictory evidence about what the risk factors are, and their relative importance (Hafez & Mullins, 2015). The lack of systematic investigation has led policy makers to develop policies and strategies that are not evidence based (Neumann & Kleinmann, 2013; Victoroff, 2005). Only “(g)reater analytical depth may eventually reconcile contradictory claims” (Wikström & Bouhana, 2016, p. 183).

While some systematic reviews have been conducted in the broader topic of radicalization, the evidence that they have synthesized with regard to risk and protective factors can generally be considered to be mixed. For example, the review prepared by the International Centre for the Prevention of Crime (ICPC 2015) identified more than half of the 32 risk factors examined as being characterized by “mixed evidence.”Gilloway et al. (2015) similarly described the evidence concerning most of the 15 risk factors examined as mixed. These findings highlight the limitations of reviews with inclusion criteria that are too broad, narrative reviews, or “vote counting” approaches. It continues to be the case that there is a lack of systematic synthesis and reconciliation of data on *risk factors for radicalization. The literature therefore continues to be highly contradictory (Allen et al., 2015; Bondokji et al., 2017; Victoroff, 2005). Even when there exists a relative consensus as to which factors represent risk, it remains unknown as to what sort of relative weight should be assigned to them (Hafez & Mullins, 2015; Haggerty & Bucerius, 2018; Rahimi & Graumans, 2016; Richards, 2003).

The most promising approach for dealing with such inconsistencies, is systematic reviews employing meta‐analytic techniques. Meta‐analysis advances parsimony, helps to settle inconsistencies, and importantly, it can provide data concerning the relative effect sizes of different individual‐level factors. Meta‐analysis therefore also provides for the possibility of a degree of reconciliation between divergent findings and debates (Pratt & Cullen, 2005). Meta‐analysis also adds an additional benefit to the study of risk factors for multiple outcomes, namely inter‐related cognitive and behavioral outcomes of the same phenomenon. It can quantify variations in magnitudes of the effects of the same risk factors across the different outcomes (Ribeiro et al., 2016). This can potentially provide important indications as to which factors may be relevant for the development between cognitive and behavioral outcomes.

## 3 OBJECTIVES

Seeking to address key gaps in the literature, this systematic review takes a field‐wide approach to identify risk and protective factors for radicalization. As opposed to traditional reviews which focus on a specific type of factor, field‐wide reviews seek to allow the literature to determine the identification of the full range of factors associated with the outcomes of interest, without predeterminations (Serghiou et al., 2016; Murray et al., 2009). This methodology enables the review to address its first primary objective, which is to identify what the putative risk and protective factors for radicalization are.

The review carries out a separate meta‐analysis for each of the identified factors and arranges the factors in rank‐orders according to the outcomes to which they pertain. This approach enables the review to address its second primary objective, namely to identify the relative magnitude of the effects for the different factors identified. In addition, this approach enables the review to address its first secondary objective, namely the identification of consistencies and differences in the risk and protective factors and their relative magnitudes across outcomes. Systematic reviews and meta‐analyses often seek to provide comparisons across related cognitive and behavioral outcomes of a given phenomenon (e.g., May & Klonsky, 2016).

The review makes extensive use of meta‐regression and moderator analysis to investigate sources of heterogeneity. This approach serves to address the review's other secondary outcome, namely to investigate consistencies in the estimates across region and radicalizing ideologies.

*Primary objectives*

1. *To identify the putative risk and protective factors for different radicalization outcomes*.
2. *To identify the relative magnitude of the effects for the different factors identified*

*Secondary objectives*

1. *To identify consistencies and differences in the putative risk and protective factors and their relative magnitudes across outcomes*.
2. *To identify consistencies and difference in the putative risk and protective factors and their relative magnitudes across regions and radicalizing ideologies*.

## 4 METHODS

The methods for this review were predetermined in a systematic review protocol published in the Campbell Collaboration journal (Wolfowicz et al., 2020). Below we re‐iterate the inclusion and exclusion criteria and the methods used in this review.

### 4.1 Criteria for considering studies for this review

#### 4.1.1 Types of studies

This review sought to extract only quantitative studies and excluded qualitative studies, including studies that are purely theoretical, provide theoretical models, literature reviews, opinion pieces, and those studies based on basic descriptive statistics. Given the nature and objectives of the review, we sought studies employing case‐control, single‐sample longitudinal, and single‐sample cross‐sectional designs. Experimental designs that also reported on cross‐sectional or longitudinal correlations were also considered eligible for inclusion. Studies were considered to be eligible for inclusion irrespective of the language in which they were written or their publication status (see “Search methods“).

In order for a study to have been included, its design must have been one that provided for the possibility of calculating an effect size. This means that in order for a study to have been eligible it must have included either a direct comparison or control group not displaying the outcome of interest, or a single sample in which there was variation on the dependent variable (Higginson et al., 2014).

For radical behaviors, studies were excluded when they compared terrorists or other radical offenders with samples of ordinary criminal offenders (e.g., Liem et al., 2018; Lyons & Harbinson, 1986). This decision was made on account of the great similarity that exists between terrorists and ordinary criminals. While important, we believe that effect sizes from such studies do not represent risk and protective factors for radicalization per se. Rather, they represent the study of similarities and differences between terrorists and ordinary criminals, which we view as being a separate topic and set of outcomes. Similarly, studies that compared terrorists of different ideologies (e.g., religious vs. right‐wing) (e.g., Smith & Morgan, 1994) or types (e.g., lone wolf vs. organizational), or studies examining macro‐level predictors of the occurrence of terrorism events (e.g., Piazza, 2006) were excluded as the nature of their comparison groups mean that they are examining outcomes that are divergent from those that are the focus of the current study.

#### 4.1.2 Types of participants

The review included studies in which the unit of analysis were individuals. The review set no limitations on the types of individuals contained in a sample in order for a study to be included. That is, the review set no limitations based on age, gender, race, religion, or the type of ideological strain being investigated (e.g., right‐wing, left‐wing, religious etc.).

#### 4.1.3 Types of factors

The review included all individual‐level factors for which a positive or negative association with the outcome of interest could be identified.

The literature discusses many classifications of factors, such as:

- Social, economic and psychological factors
- Proximal and distal factors
- Push and pull factors
- Individual, family, school, peers, and social factors

There are many other classifications and this review includes factors from all such categories. We note the review discusses factors in the context of four domains:

- Sociodemographic/background characteristic factors
- Attitudinal/subjective belief factors
- Psychological/personality trait factors
- Experiential factors

The review excludes all factors that are not measured at the individual level, for example:

- Meso‐level factors: Community level deprivation, population density, and so forth
- Macro level factors: GDP, GINI, and so forth
- Time‐series factors: The occurrence of specific events (e.g., terror attacks), or rates of social phenomena (e.g., crime rates).

Additionally, the review did not include experimental manipulations as factors since combining these with nonmanipulated versions of the same factors would lead to methodological inconsistencies.

#### 4.1.4 Types of outcome measures

##### Inclusion

The review sought to include studies which measured cognitive and behavioral radicalization outcomes. However, it is known that the literature includes a heterogeneous range of proxies for such outcomes, and not all measures necessarily capture the substantive and conceptual elements of the types of radicalization that this review was interested in. To ensure consistency across the outcomes measured in included studies, inclusion was limited to studies whose outcome variable(s) were in line with at least one of three relevant outcomes derived from McCauley and Moskalenko's (2017) two‐pyramid model (TPM) of radicalization:

1. *Radical attitudes*: Justification/support for radical behaviors carried out in the name of a cause.
2. *Radical intentions*: Willingness/intentions towards engagement in radical behaviors in the name of a cause.
3. *Radical behaviors*: Actual involvement in violent radical behaviors in the name of a cause, including terrorism.

In line with the TPM, radical behaviors include subterroristic radical violence and terrorism. The former refers to acts of violence in the name of a cause or ideology that is carried out against persons or property and is usually nonlethal. These types of actions are illegal but fall short of terrorism laws in the state in which the action is carried out. The latter refers to acts of violence in the name of a cause or ideology that has a significant potential to be lethal or is otherwise intended to be lethal, and is considered to be an act of terrorism under the laws of the state in which the action(s) is carried out. In many countries, attacks against critical infrastructure that have the potential to seriously damage or destroy them, or otherwise do, would also be considered an act of terrorism.

The review set no specific limitations on the types of outcome measures. That is, the review included studies employing both validated and nonvalidated instruments, originally developed instruments, and measures that were made up of multiple and single items measures, or which were dichotomous, ordinal, or continuous measurements. Measures of outcomes were included when derived from self‐reports, family reports, administrative reports (e.g., government or law‐enforcement), practitioner/clinical reports, and open‐source database‐generated data.

##### Exclusion

Applications of the TPM have found important differences between “activists” and “radicals,” as well as attitudes and intentions towards these behaviors, and the factors which predict them. The primary distinguishing feature between these categories being that activism behaviors are generally legal, nonviolent behaviors, whereas radical behaviors are generally illegal and violent (Decker & Pyrooz, 2018; McCauley & Moskalenko, 2017). As such the review excluded studies which examined outcomes that measured:

- Justification/support for the use of normative and non‐normative actions that make no specific reference to the use of violence.
- Willingness/intentions towards engagement in normative and non‐normative actions that make no specific reference to the use of violence
  a. Such as the willingness to self‐sacrifice, or give up one's life, when no reference to the use of violence is included
- Actual involvement in normative and non‐normative actions that do not include violence or which do not constitute breaches of terrorism laws in the countries from which the sample was derived.

Further, studies which examined justification/support for, willingness/intentions towards, or actual involvement in violence without reference to a defence of a cause or ideology were excluded. These exclusions result in the review erring on the side of caution in order to ensure the outcomes relate without doubt to those we sought to assess.

#### 4.1.5 Duration of follow‐up

As the review expected to collect data primarily from cross‐sectional studies, no limitations were placed on the duration of follow‐up for a longitudinal study to be included.

#### 4.1.6 Types of settings

It is well known that differences in political, socioeconomic and cultural contexts affect the types of factors that contribute to radicalization in different countries (Brockhoff et al., 2016). Studies that have examined support for suicide bombings for example, have found significant differences in the direction and magnitude of the effects for risk and protective factors between western and non‐western countries (e.g., Zhirkov et al., 2014). The issue of heterogeneity across contexts provides for the methodological justification for systematic reviews to limit their focus to particular types of countries, such as high and low‐medium income countries, or democratic and nondemocratic countries (Higginson et al., 2018; Litmanovitz & Montgomery, 2016; Murray et al., 2018; Shenderovich et al., 2016).

Given that there is no established norm as to whether systematic reviews should distinguish countries by income level or the system of government, in this review the approaches are combined. High‐income countries are considered to be countries who are member states of the Organisation for Economic Co‐operation and Development (OECD), and democratic states are those states ranked as being full or partial democracies by the Democracy Index. In cross‐matching countries from these two sources, all OECD countries, with the exception of Turkey and Columbia are considered countries eligible for inclusion. In order for a study to be included, its sample must originate from one of the eligible countries, and data had to have been collected in a year in which the country was eligible. This means, for example, that studies from Columbia prior to 2020 would be excluded, since Columbia only became a member of the OECD in 2020.

Additionally, in cases where studies' samples were drawn from multiple countries, and included participants originating from ineligible countries, the study would be included if at least 50% of the sample originated from eligible countries (Table 1).

**Table 1:** Countries eligible for inclusion in the review

Eligible countries
Australia, Austria, Belgium, Canada, Chile, Columbia, Czech Republic, Denmark Estonia, Finland, France, Germany Greece Hungary, Iceland, Ireland, Israel, Italy, Japan, Korea, Latvia, Lithuania, Luxemburg, Mexico, the Netherlands, New Zealand, Norway, Poland, Portugal, Slovakia, Slovenia, Spain, Sweden, Switzerland, UK, United States

### 4.2 Search methods for identification of studies

#### 4.2.1 Electronic searches

Electronic searches were performed across a large number of electronic databases, organizational databases, and specialty journals (Table 2). While these searches were performed in English, many items written in other languages are indexed in these databases as well. Given that these items are indexed in English, it was possible for them to be screened at the first and second stages in English. Where necessary, items in other languages were sent for translation.

**Table 2:** Search locations

Search locations
*Systematic reviews:* Campbell Collaboration library, Cochrane Library, DARE
*Journals and other publications:* Criminal Justice Abstracts, ERIC, ISI Science/Social Science Citation Index, Open dissertations, Medline, Political Science Complete, PsycINFO, PubMed, Social Science Research Network e‐library (SSRN), Social Care Online, SocIndex, Social work abstract, Sociological abstracts, Bibsys, ProQuest dissertations, Violence and Abuse abstracts

Searches were ongoing from December 2019 to April 2020.

In addition to the above, searches were conducted in the databases of the following organizations: The National Consortium for the Study of Terrorism and Responses to Terrorism (START), National Institute of Justice (NIJ), NCJRS (National Criminal Justice Reference Center), The Crime Prevention Council, Sweden (BRÅ).

Furthermore, searches were conducted in specialty journals, including: Perspective on Terrorism, and Journal of Deradicalization.

Extensive supplementary searches were carried out on Google scholar on an ongoing basis in order to identify the most up to date studies which had yet to be indexed in the electronic database.

In order to ensure a more comprehensive review, supplementary searches were also carried out in German and Dutch, the local languages of countries known for producing research relevant to the topic of the review. German sources were searched for in two databases, namely the German National Library and GESIS. Dutch sources were searched for in PiCarta. These databases do not allow for the use of the Boolean search strings used in the English language databases. As such, they were searched for three key terms: Terrorism, Radicalization, and Extremism.

#### 4.2.2 Searching other resources

In addition to the electronic searches, we contacted a number of leading researchers to try to identify missing studies. When we contact them we provided them with the review's topic and inclusion criteria, as well as a list of the included studies that we had collected by the time and inquired as to whether they were aware of any additional studies that would potentially meet the inclusion criteria that we had not identified. We also reviewed the bibliographies of other systematic reviews.

### 4.3 Data collection and analysis

#### 4.3.1 Selection of studies

##### Title and abstract screening: Double screening

Search results were downloaded in files that contained their full reference information, titles, and abstracts and subsequently imported into the EndNote X8 reference manager program. The lead authors, together with a team of 4 trained research assistants, used the “reference” window to carry out the initial screen by manually scanning the titles and abstracts as they appeared. When a study's abstract included information indicating that it may meet the inclusion criteria, it was transferred to a new folder which would undergo a second screening. The lead author carried out the second screening using the same approach as the first screening. All items that were believed to meet the inclusion criteria were transferred to a new folder which would undergo the third screening, as part of which a full‐text screening was carried out.

##### Full text screening

For the full text screening, the researchers downloaded the PDF file of the full paper and attached it to its reference in EndNote. The researchers then examined the methodology section of the paper, specifically the sections describing the outcome measures, and the sample. The researchers assessed the following:

1. The study's outcome is in line with the inclusion criteria and measures Radical attitudes and/or Radical intentions, and/or Radical behaviors as per the predetermined definitions (Y/N)
2. The study's sample is made up of individuals (Y/N)
3. The study examines at least one individual level factor (Y/N)
4. The study's sample is derived from an eligible country (Y/N)
5. The study uses an eligible design
6. The study provides sufficient information to calculate at least one effect size (Y/N)
  When all of the above criteria (1‐6) were screened as 'Yes', the study was moved to a new folder in the shared EndNote library entitled “Final inclusion.” At the analysis stage, a further inclusion criterion was imposed:
7. The study is able to contribute at least one unique effect size to the meta‐analysis (Y/N)

#### 4.3.2 Data extraction and management

Data extraction was carried out by a team of five researchers. A shared Microsoft Excel spreadsheet was created which included multiple sheets. The first sheet was used for filling out the study‐level characteristics of included studies:

- Study name
- Author name(s), year of publication
- Sample size(s)
- Outcome variable construct(s)
- Outcome variable measurement (Dichotomous/ordinal/continuous/discrete)
- Mean age of sample(s)
- Proportion of males (%) in the sample(s)
- Year of data collection
- The number of effect sizes extracted from the study
- Country from which the sample(s) was derived
- The ideological strain that the study examined (e.g., right‐wing, left‐wing, religious, ethno‐nationalist, nonspecific/mixed samples
- Publication status
- Type of study design
- Makeup of control/comparison group

A separate workbook was then created for each of the outcomes examined in the review (titled: radical attitudes, radical intentions, and radical behaviors); in each of these workbooks a separate sheet was created for each factor and labeled. For each effect size, a separate row was created that included the following information:

- Study name
- The standardized effect sizes (*r* correlation)
- Sample size
- Mean age of samples
- Proportion of males
- Year of data collection
- Region from which the sample was derived (EU, US, and other countries)
- Ideological strain examined in the study

Where the main study documents failed to include any of the above information, searches were conducted to identify supplementary materials. Where supplementary materials could not be identified, or where identified supplementary materials also failed to provide the missing information, the authors of the relevant documents were contacted directly by the project team (See “Missing data” below).

#### 4.3.3 Assessment of risk of bias in included studies

Risk of bias was assessed by using the coding fields contained in the extraction tool (see Supplementary materials). The factors assessed were:

- Did the study report its sample in replicable detail?
- Does the study list inclusion/exclusion criteria for participant inclusion?
- Did the study report its sampling method?
- Did the study use validated outcome measures?
- Did the study use validated instruments to measure independent variables?
- Did the study use data that overlaps with other studies?
- Did the study fail to report on nonsignificant findings?
- Did the study fail to analyse important factors noted in its sample description?

#### 4.3.4 Calculation of effect sizes

All effect sizes were calculated as *r* and subsequently transformed to Fisher's *Z* in order to approximate a normal sampling distribution and achieve a more stable variance across different values (Borenstein et al., 2009; Rosenthal, 1984).

In line with previous research, the current review gave preference to bivariate correlations, which provide for a consistent and uncontaminated measurement of effect metrics for the same factors between studies (Hanushek & Jackson, 1977; Hunter & Schmidt, 2004; Pratt et al., 2014). Bivariate effect sizes were derived from zero‐order correlation matrices, but were also calculated from summary statistics such as *t* tests, *F* tests, *χ* ^2^ tests, and ANOVAs, and other classical hypothesis tests. In other cases, bivariate effect sizes were calculated from descriptive data such as means and standard deviations, frequencies, and binary proportions. In all such cases, we used Lipsey and Wilson's (2001), “Practical Meta‐Analysis Effect Size Calculator” available through the Campbell Collaboration website. The effect sizes were calculated as Cohen's *d* and subsequently converted to *r* using the formula:

$$r=\frac{d}{\sqrt{4+d^{2}}}$$

However, as anticipated, some studies did not provide sufficient information to calculate a bivariate effect size; these studies only provided the results of a range of different types of regression models together with basic sample level descriptive data. In such cases, we attempted to identify sources for the missing data (see Section 4.3.9), including contacting the authors (Aloe & Thompson, 2013). Where we were unable to acquire the missing data, we standardized the partial effect sizes derived from the regression models to be used as supplementary effect sizes (e.g., Najaka et al., 2001; Wong et al., 2010). While not ideal, this approach is preferable to conducting multiple separate meta‐analyses for each risk factor split by effect size measurement type, which would entail losing important data (Borenstein et al., 2009). In case in which there were more than two bivariate effect sizes and two standardized partial effect sizes in a single analysis, meta‐regression and moderator analysis (see Section 4.3.10) were used to identify whether combining these effect sizes had any effect on the pooled estimates (Aloe et al., 2016).

With regard to the standardization of partial effects sizes, there are no standard conventions (Aloe & Thompson, 2013). As such, we adopted a number of widely accepted methods for each of the different types of measures that we encountered, as described below.

1. For linear regression models where the independent variable (IV) and dependent variable (DV) are both continuous, *r* was calculated as
$$r=\frac{SD_{x}B}{SD_{y}}$$
2. In situations in which *SD* were not reported, thereby precluding using the above approach, and also in situations in which the IV was dichotomous and the DV was continuous, and in situations in which the IV was ordinal or continuous and the DV was dichotomous, *r* was calculated based on the *t* ratio (B/SE) using the following formula:
$$r=t/\sqrt{t^{2}+}n-2$$
3. In instances in which both the IV and the DV were dichotomous measures, and only B was reported, we first calculated Cohen's *d* and then subsequently converted *d* to *r* as per the above. In such situations, Cohen's *d* was calculated as:
$$d=B\left(\;,\frac{\sqrt{3}}{\pi }\right)$$
4. In situations in which only the odds ratio (Exp. B) was reported, we calculated *r* by first converting the odds ratio to Cohen's *d* and then subsequently converted *d* to *r* as per the above.

##### Calculation of standard errors

For all effect sizes derived from bivariate sources, the standard error was calculated as the square root of the variance of the *z* transformed correlation, which is calculated as 1/*N* – 3 (N = sample size).

With regard to effect sizes standardized from regression models, standard errors were calculated based on a rescaling of the model‐based standard error, which is calculated as:

$${\mathrm{se}}_{r}=\frac{r\;⁎\;{\mathrm{SE}}_{B}}{B}$$

In situations in which the *SE* was not reported, it was calculated from reported confidence intervals in order to enable the above calculation to be made. In the case of 95% confidence intervals, the *SE* was calculated as:

$$\mathrm{SE}=\frac{{\mathrm{CI}}_{\mathrm{upper}}-{\mathrm{CI}}_{\mathrm{lower}}}{1.96}$$

#### 4.3.5 Independence of effect sizes

When conducting meta‐analysis, it is important to reduce or eliminate potential dependence among effect sizes so that dependent effect sizes are not over‐weighted (Lipsey & Wilson, 2001). In this study, common issues affecting dependence were absent, since no single study (or sample within a study containing multiple samples) contributed more than a single effect size for a given factor, and each factor was examined in a separate analysis (Pinquart, 2017; Van den Noortgate et al., 2015).

A second issue relating to dependence concerns the possibility that multiple studies may be based on the same dataset and report on the same factors. We carefully scrutinized the data by searching for repeated instances of sample size, mean age, and proportion of males in the studies. Whenever it was identified that more than one study was reporting on the same factor derived from an overlapping dataset, an internal meta‐analysis was performed and the pooled estimate was used as the input for these studies (Higginson et al., 2018).

#### 4.3.6 Dealing with missing data

Whenever a study had missing data the following actions were taken in order:

1. Search for supplementary materials
2. Search for access to the original data and replicate the model
3. Search for other studies by the author(s) that use the same data
4. Search for studies by other authors that use the same data
5. Contact the author(s) with a request to provide the missing data

Missing data was successfully retrieved from online supplementary materials for 21 studies, from the re‐processing of original data for 6 studies, and directly from authors for eight studies.

#### 4.3.7 Assessment of heterogeneity

Heterogeneity was assessed using Cochran's *Q* (and its associated *χ* ^2^ *p* value) as well as the *I* ^*2*^ statistic. *I* ^*2*^ scores of >75 indicate high heterogeneity, whereas, >50 moderate, >25 low, <25 very low. When *I* ^*2*^ = 0 it indicates an absence of heterogeneity.

#### 4.3.8 Assessment of reporting biases

Given the large number of factors that are often contained in field‐wide reviews it was expected that it would be impractical to present funnel plots for each factor analysed. As such, publication bias was assessed using two methods that are based on the funnel plot, the Trim‐and‐Fill method (Duval & Tweedie, 2000a, 2000b) and Egger's regression test (Egger et al., 1997). In Egger's regression test, the standardized effect sizes are regressed on their precisions. This is the equivalent of a weighted regression of the effect sizes on their standard errors, in which weighting is on the inverse variance. In the absence of publication bias, the intercept is expected to be zero (Rothstein et al., 2005). A statistically significant intercept above zero indicates the presence of publication bias. In the trim‐and‐fill method, asymmetric funnel plots (where smaller studies are skewed to one side of the bottom of the plot) are identified as indicators of publication bias. The method employs an iterative approach to removing the studies causing the asymmetry, estimating the number of studies missing due to this bias and their effect sizes, and then re‐computes a new, adjusted effect size (Rothstein, 2008). As both methods suffer from limitations (Sterne et al., 2000), they have often been used complimentarily, including in risk factor related research (e.g., Assink et al., 2015; Vazsonyi et al., 2017).

#### 4.3.9 Data synthesis

Meta‐analysis was performed using Biostat's Comprehensive Meta‐Analysis (CMA) software (Borenstein et al., 2009). Random effects models were used in order to account for the anticipated heterogeneity of the studies, which is common when dealing with correlational data from observational studies, and also in studies dealing with violence related cognitions and behaviors. Effect sizes pertaining to risk and protective factors are known to be more heterogeneous than those for interventions, in part because they deal with heterogeneous populations and segments of the different populations. CMA's Random effects models calculate estimates based on the inverse variance, which is derived from the sample size associated with each effect size. CMA V3 utilizes the Method of Moments random effects estimator for *τ* ^2^, the between‐study variance (DerSimonian & Laird, 1986).

We categorized each factor based on a careful assessment of the measurement constructs contained in the studies and the outcome measure, and combined effect sizes that measured the same construct, or conceptually similar constructs for the same outcome. For the most part, studies were quite clear as to the nature of the factors, with detailed descriptions of the factors' name, construct, and measurement in the methodology sections. In order to provide for the most meaningful analysis, we tried to avoid combining factors that were conceptually related but distinct. For example, while Law legitimacy and Law abidance are closely related, in analysing the items used to measure these constructs it was determined that they were conceptually distinct. In other cases, such as Authoritarianism and Fundamentalism, a close inspection of the items used to measure these factors across different studies revealed that they often only differed in how they were labeled, with labeling differing between studies analysing different ideological strains (e.g., Right‐wing, Islamist, or nonspecific/mixed ideologies). The literature also indicates that these factors are nearly indistinguishable, and common scales were used in most of the studies reporting on these factors (Altemeyer & Hunsberger, 1992, 2004, 2005). Supporting Information Appendix A includes a list of the factors included in the review with examples of the common constructs used in studies.

A separate meta‐analysis was conducted for each of the identified factors. We present the results of the meta‐analyses as *r* correlations with 95% confidence intervals in a series of rank‐ordered tables, with each row representing a separate analysis for a single factor.

#### 4.3.10 Subgroup analysis and investigation of heterogeneity

Only a few studies included data that would have enabled any sort of meaningful subgroup analysis (e.g., based on gender, ethnicity, or religion). Given the limited number of these studies, such analysis would have only been possible for a small number of factors. Given that we were able to conduct an extensive array of univariate meta‐regression analyses to assess the impact of study‐level characteristics on heterogeneity, these subgroup analyses were not conducted.

The protocol predefined that meta‐regression analyses would be used to assess the effects of the following factors on pooled estimates:

- Region/country from which the sample was derived (EU, United States, and Other)
- Ideological strain examined by the study (nonspecific/mixed ideologies, right‐wing, left‐wing, Islamist, and Other (which included separatist and ethno‐nationalist).
- Year of data collection
- Mean age of study sample
- Proportion of males in study sample
- Effect size derivation (Bivariate, Standardized partial effect size)

While we had hoped to examine individual countries, few analyses provided a sufficient number of studies to enable this type of inquiry. In order to provide consistency across all analyses, we grouped the countries by region. Given that the majority of the studies were from the EU, this region was used as the reference category. With regard to ideological strain, given that the majority of the studies examined nonspecific or mixed samples, this was used as the reference category. For moderator analyses between‐group heterogeneity was assessed using Cochran's *Q*.

#### 4.3.11 Sensitivity analysis

Sensitivity analysis was performed for each factor that included 3 or more effect sizes using the “leave‐one‐out” method. This approach uses an iterative method in which the meta‐analysis is repeated *k* times (*k* = the number of effect sizes in the analysis) and a different study is excluded at each iteration. We inspected the results to identify whether a single effect size had a significant influence on heterogeneity (Viechtbauer & Cheung, 2010). This was assessed by examining whether the removal of any single study caused heterogeneity to be reduced by at least one level, with the levels being set at; very low (*I* ^*2*^ < 25), low (*I* ^*2*^ > 25), moderate (*I* ^*2*^ > 50), and high (*I* ^*2*^ > 75). We reported on factors for which the removal of a single study led to a significant reduction in heterogeneity.

## 5 DEVIATIONS FROM THE PROTOCOL

The only deviations from the protocol pertain to a small number of fields in the extraction tool, and the risk of bias items in particular (Wolfowicz et al., 2020). Two items were added and four items were dropped as a result of the nature of the nature and in order to provide a higher level of useful information concerning the included studies.

The following items were included in the review that differed slightly from the protocol:

1. Did the study report its sampling method?
2. Did the study use data that overlaps with other studies?

The following items were included in the original protocol but were omitted in the current study:

1. Prospective study: Was the study prospective (ie the sample was selected prior to the onset of radicalization or involvement in radical activity)?
  This was excluded due to the cross‐sectional nature of the overwhelming majority of studies.
2. Outcome descriptor: Was the criteria for fitting “radical”/“radicalization”/‘recruited described in replicable detail?
  This was excluded as most studies used validated or widely accepted measures of radicalization outcomes, which was coded.
3. Risk factor description: Were all factors described in replicable detail?
  This was excluded as we already coded for whether risk factors used validated measures, in which case they were described in replicable detail.
4. Risk factor timing: Were all factors either measured before the onset of radicalization or involvement in radical activity, or measured retrospectively to a time prior to radicalization or involvement in radical activity?

This item was excluded due to the cross‐sectional nature of the overwhelming majority of studies.

## 6 RESULTS

### 6.1 Description of studies

#### 6.1.1 Results of the search

**Figure 5:**
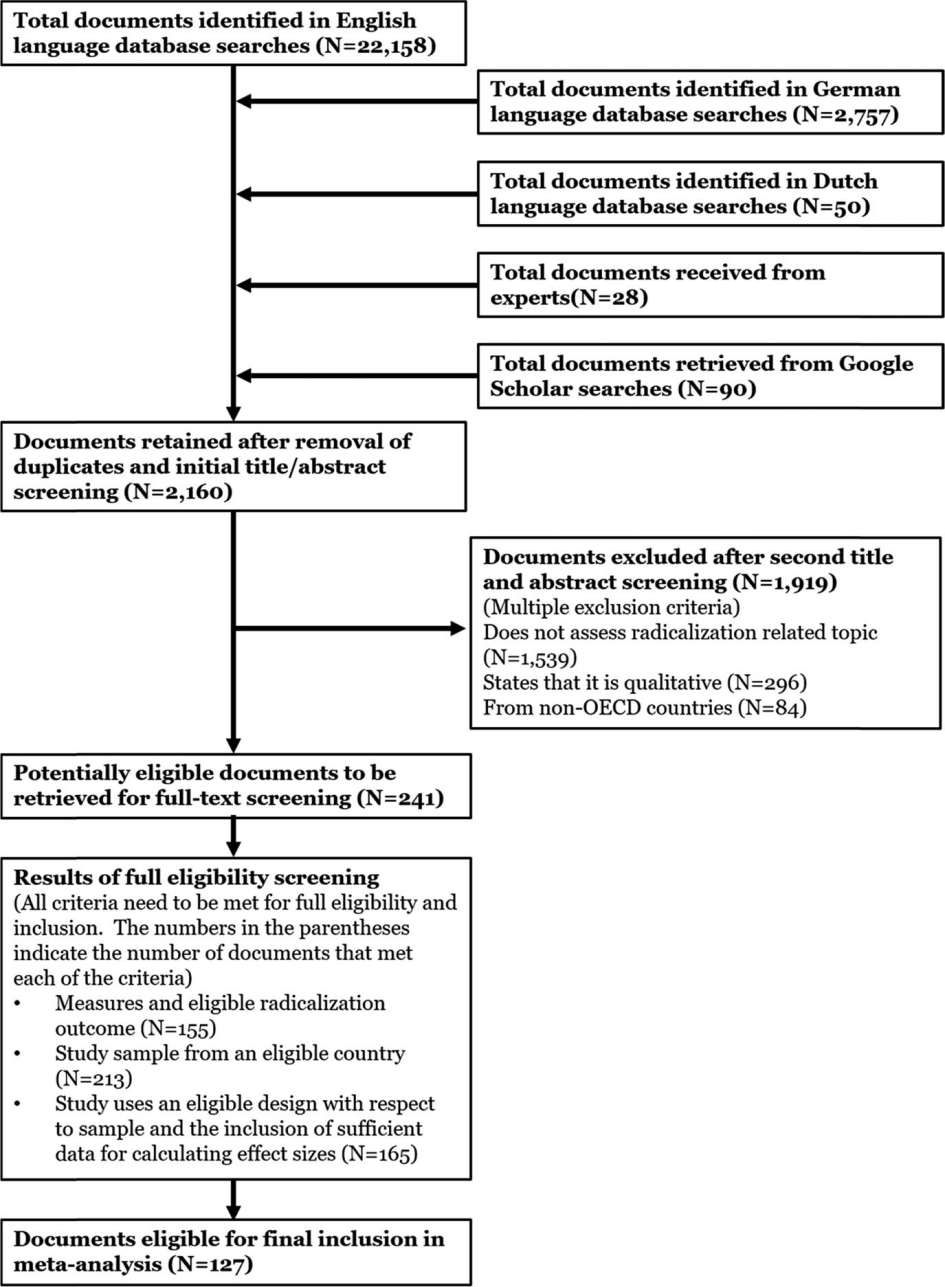
Flowchart of search and screening process

The results of the systematic searches and screening process are displayed below in Figure 5. The primary English‐language searches resulted in the identification of over 22,000 items. An additional 2925 items were retrieved from a combination of German and Dutch language databases, as well as items sent to the research team by authors. These additional items also included 90 items that were retrieved from Google Scholar by the research team and were added to the first sample screening. The 28 items received directly from authors who had been contacted were screened separately.

English studies were screened by two of the senior researchers together with trained research assistants. The German studies were screened by a native German speaking research assistant and the Dutch studies were screened by a native Dutch speaking research assistant. A single study in Spanish was provided and translated, which enabled its screening in English. Three studies in French, including one which was sent to us by one of the experts we had contacted were screened by the main author who had sufficient French language skills to assess the items.

During the initial screening it was evident based on titles and abstracts that the overwhelming majority of the items did not relate to the field or topic of interest. The removal of these studies resulted in the exclusion of 19,998 items, with 2160 items moving on to the second screening stage. During secondary screening, the titles and abstracts were more carefully scrutinized. While many of the items dealt with topics related to “terrorism,” “extremism,” or “radicalism,” it was clear that for these and many other items, they were not assessing radicalization or radicalization analogous outcomes. A large number of items clearly stated in their titles or abstracts that the study was “qualitative,” or based on “case studies,” and it was evident that they did not meet the methodological inclusion criteria. A number of other studies which appeared to meet the inclusion criteria for the topic, clearly stated in their titles or abstracts that the study was conducted in a country outside of the eligible country list.

The final screening stage involved a 'full‐text' reading of each item by the first author and the research assistants. Another senior researcher read half of the items. The methodology sections were reviewed first in order to identify the nature of the data and sample and outcome measures. While many of the studies met between 1 and 4 of the criteria for inclusion, only 127 studies met all inclusion criteria and were subsequently submitted for inclusion in the review (Figure 5).

#### 6.1.2 Included studies

A total of 127 studies were screened as being eligible for inclusion. The full references for these studies appear in the reference section. A description of the included studies can be found below (Table 3). The included studies were published between 2003 and 2020. Of these, 50% were published from 2018 to 2020. A total of 42 of the included items included more than one study, or reported on more than one individual sample. This meant that the total number of individual studies/samples derived from the included items was 206.

**Table 3:** Description of included studies

#	Name	*N*	Outcome	Males%	Age (mean)	Year of data collection	Country	Ideological strain	Language	Publication status
1	Abdi (2019)	279	A	54.7	24.36	2018	US and Canada	Islamist	English	Thesis
2	Acevedo and Chaudhary(2015)	829	A	53.3	41.12	2007	US	Islamist	"	Journal
3	Adam‐Troian et al. (2019)					2018			"	"
	Study 1	249	I	20.5	19.35	"	France	Mixed	"	"
	Study 2	110	A	42.7	29.97	"	France	Islamist	"	"
	Study 3	279	I	12.5	19.14	"	France	Mixed	"	"
4	Ahearn et al. (2020)	608	A				UK	Islamist	"	Journal
5	Allington et al. (2019)	4897	A	48	46.35	2019	UK	LWE	"	Report
6	Altunbas and Thornton (2011)	1440	B	52.1	32.49	2009	UK	Islamist	"	Journal
7	Baier (2010)	18631	A	N/A	N/A	2007	Germany	Mixed	German	Report
8	Baier et al. (2016)			50.9	N/A	2013			"	Chapter
	Right‐wing sample	4965	A/B	N/A	N/A	"	Germany	RWE	"	"
	Left‐wing sample	3258	A/B	N/A	N/A	"	Germany	LWE	"	"
	Islamist sample	383	A/B	N/A	N/A	"	Germany	Islamist	"	"
9	Becker (2019)	1757	B	90	33.59	2018	US	Mixed	English	Journal
10	Becker (2020)	503	I	39.2	23.014	2018	US	Mixed	English	Journal
11	Bélanger et al. (2014)	76	I	63.16	31		US	LWE	English	Journal
12	Bélanger, Moyano, et al. (2019)					N/R			English	Journal
	Study 1	209	I	55.02	33.01	"	US	LWE	"	"
	Study 2	220	I	54.09	37.73	"	US	LWE	"	"
	Study 3	172	I	44.19	N/R	"	US	LWE	"	"
	Study 4	115	I	40.87	34.3	"	US	LWE	"	"
	Study 5	167	I	60.48	40.23	"	US	LWE	"	"
13	Bélanger, Schumpe, et al (2019)					N/R			English	Journal
	Study 1	470	A	43.62	32.67	"	Canada	Mixed	"	"
	Study 2	233	A	70.82	34.23	"	Spain	Mixed	"	"
	Study 3	319	A	55.17	40.49	"	US	Mixed	"	"
14	Beller and Kröger (2020)								English	Journal
	Study 1	1050	A	53	41.09	2007	US	Islamist	"	"
	Study 2	1033	A	55	NA	2011	US	Islamist	"	"
15	Berger (2016)	1214				2006			English	Journal
	UK sample	275	A	52	33	"	UK	Islamist	"	"
	France sample	374	A	49	32.97	"	France	Islamist	"	"
	Germany sample	295	A	46	37.08	"	Germany	Islamist	"	"
	Spain sample	270	A	77	32.93	"	Spain	Islamist	"	"
16	Berrebi (2007)	41828	B	100	29.03	2002	Israel	Islamist	"	Journal
17	Besta et al. (2015)	179	A	57.5	20.04	2014	Poland	Mixed	"	Journal
18	Bhui et al. (2014a)	608	A	54	30	2013	UK	Islamist	"	Journal
19	Bhui et al. (2016)	608	A	54	30	2013	UK	Islamist	"	Journal
20	Bhui et al. (2019)	618	A	49.2	N/R	2018	UK	Mixed	"	Journal
21	Brettfeld and Wetzels (2007)	485	A	53.2	15.8	2005	Germany	Islamist	German	Report
22	Capelos and Demertzis (2018)	2557	A/B/I	N/R	N/R	2015	Greece	Mixed	English	Journal
23	Cardeli et al. (2020)	534	A	61	22.15	2015	US	Islamist	English	Journal
24	Charkawi et al. (2020)	187	A	50	N/R	2017	Australia	Islamist	English	Journal
25	Cherney and Murphy (2019)	800	A	50.5	34.9	2014	Australia	Islamist	English	Journal
26	Clemmow et al. (2020)	2233	B	48	30.23	N/R	Mixed	Mixed	English	Journal
27	Coid et al. (2016)	3679	A	100	25.9	2011	UK	Mixed	English	Journal
28	Dahl (2019)	1987	A	47.9	15.03	2015	Sweden	Mixed	English	Journal
29	De Waele and Pauwels (2016)	723	A	35.7	NA	2013	Belgium	Mixed	English	Chapter
30	Decker and Pyrooz (2018)	802	I	100	40.82	2016	US	Mixed	English	Journal
31	Delia Deckard and Jacobson (2015)	1200	A	48	40.39	2014	EU (Mixed)	Islamist	English	Journal
32	Doosje et al. (2012)	1086	A/I	55.6	16.64	2008	The Netherlands	RWE	English	Journal
33	Doosje et al. (2013)	131	A/I	61	17	2008	The Netherlands	Islamist	English	Journal
34	Egger and Magni‐Berton (2019)	31554	A			2008	EU (Mixed)	Islamist	English	Journal
35	Ellis et al. (2015)	79	A	100	20.76	2014	US, Canada	Islamist	English	Journal
36	Ellis et al. (2016)	374	A	62	21.3	2014	US, Canada	Islamist	English	Journal
37	Ellis et al. (2019)	213	A	100	22	2015	US	Islamist	English	Journal
38	Faragó et al. (2019)	1000	A/I	N/R	N/R	N/R	Hungary	Mixed	English	Journal
39	Feddes et al. (2015)	46	A/I	100	16.93	2014	The Netherlands	Islamist	English	Journal
40	Fodeman et al. (2020)	356	I	45.79	N/R	2016	US	Islamist	English	Journal
41	Frissen (2019)	1872	A	47.26	17.14	N/R	Belgium and Canada	Mixed	English	Thesis
42	Frissen et al. (2019)	317	A	40.69	18.14	N/R	Belgium and Canada	Mixed	English	Chapter
43	Frounfelker et al. (2019)	2037	I	51.9	18.5	2017	Belgium	Mixed	English	Journal
44	Goede et al. (2019)	6863	A		47.4	2018	Germany	Mixed	German	Report
45	Gøtzsche‐Astrup (2019a)					2017			English	Journal
	Study 1	2317	I	53	26.3	"	US	Mixed	"	"
	Study 2	2489	A	51	42.5	2016	US	Mixed	"	"
46	Gøtzsche‐Astrup (2019b)			N/R	N/R	N/R			English	Pre‐publication
	Study 1	956	I	"	"	"	US	Mixed	"	"
	Study 2	1944	I	"	"	"	US	Mixed	"	"
	Study 3	897	I	"	"	"	US	Mixed	"	"
47	Gøtzsche‐Astrup (2020)								English	Journal
	Sample 1	1188	I	48.2	43.78	2017	Denmark	Mixed	"	"
	Sample 2	1300	I	47.9	41.63	2017	US	Mixed	"	"
	Sample 3	401	I	24	37	2017	Denmark	Mixed	"	"
48	Gousse‐Lessard et al. (2013)								English	Journal
	Study 1	110	A	39.1	32.26		Canada	LWE	"	"
	Study 2	131	I	24.43	32.14		Canada	LWE	"	"
	Study 3	190	I	32.63	38.26		Canada	LWE	"	"
49	Groppi and Chin (2018)	440	I	68.86	N/R	2016	Italy	Islamist	English	Thesis
50	Harms (2017)	765	B	90	38.25	2007	US	Mixed	English	Journal
51	Jackson et al. (2013)	1017	A	N/R	23	2009	UK	Mixed	English	Journal
52	Jahnke et al. (2020)					2018			English	Journal
	Study 1	3715	A	48	14.6	"	Germany	Mixed	"	"
	Study 2	303	I	47.9	24.23	"	Germany	Mixed	"	"
53	Jasko et al. (2016)	1496	B	90	34.24	2014	US	Mixed	English	Journal
54	Jensen et al. (2016)	1473	B	90	34.18	2016	US	Mixed	English	Report
55	Jones et al. (2020)					N/R			English	Journal
	Study 1	409	A	38.88	38.46	"	US	RWE		
	Study 2	209	I	25	25.57	"	US	RWE		
56	Kalmoe (2014)					2010			English	Journal
	Study 1	412	A			"	US	Mixed		
	Study 2	512	A			"	US	Mixed		
	Study 3	384	A	27.3	49	"	US	Mixed		
57	Kerodal et al. (2016)	305	B	N/R	N/R	2010	US	RWE	English	Journal
58	Krueger (2008)	1063	B	54.6	38.11	2006	US	Islamist	English	Journal
59	Kunst et al. (2018)								English	Journal
	Study 1	201	I	56.7	34.6		US	LWE	"	"
	Study 2	215	I	40.9	24.99		Norway	LWE	"	"
	Study 3	234	I	45.7	36.13		US	LWE	"	"
60	LaFree et al. (2018)	1471	B	90	34.2	2016	US	Mixed	English	Journal
61	LaRue (2012)	1050	A	N/R	N/R	2006	US	Islamist	English	Thesis
62	Lemieux and Asal (2010)	2932	A	64	54	2008	US	Mixed	English	Journal
63	Littler (2017)	1561	A	42.5	52.07	2008	UK	Mixed	English	Journal
64	Ljujic et al. (2020)	6283	B	87.3	30.74	2017	The Netherlands	Mixed	English	Chapter
65	Lobato et al. (2018)								English	Journal
	Study 1	133	I	56.64	15.89		Spain	Islamist	"	"
	Study 2	126	I	44.44	15.89		Spain	Mixed	"	"
	Study 3	98	I	40.82	28.04		Spain	Islamist	"	"
	Study 4	167	I	41.92	28.04		Spain	Mixed	"	"
66	Lobato et al. (2020)	214	I	58.41	33.48	2017	Spain	Ethno‐nationalist	English	Journal
67	Loughery (2018)	77	I	38.96	24.12		Sweden	Islamist	English	Thesis
68	Lyons (2015)								English	Thesis
	Study 1	198	A	39.39	27.42		US	Islamist	"	"
	Study 2	204	A	36.76	25.3		Germany	Islamist	"	"
	Study 3	139	A	22.30	21.41		US	Islamist	"	"
69	Macdougall et al. (2018)	183	A	47	27.2	2015	The Netherlands	Mixed	English	Journal
70	Mahfud and Adam‐Troian (2019)					2018			English	Journal
	Study 1	776	I	28.48	32.02	"	France	Mixed	"	"
	Study 2	511	I	9.1	19.39	"	France	Mixed	"	"
71	Manzoni et al. (2019)			49.7	17	2017			German	Report
	Right‐wing sample	2932	A	N/R	N/R		Switzerland	RWE	"	"
	Left‐wing sample	5697	A	N/R	N/R		Switzerland	LWE	"	"
	Islamist sample	476	A	N/R	N/R		Switzerland	Islamist	"	"
72	McCauley (2012)	1050	A	53	42.37	2007	US	Islamist	English	Journal
73	Miconi et al. (2019)	1680	A	29	N/R	2016	Canada	Mixed	English	Journal
74	Moskalenko and McCauley (2009)								English	Journal
	Study1	140	I/B	13	19.6	2005	US	Mixed		
	Study2	429	I/B	52	46	"	US	Mixed		
75	Moyano (2011)			45.2	15.71	2009			Spanish	Thesis
	Sample 1	282	A	"	"	"	Spain	Islamist	"	"
	Sample 2	1670	A	"	"	"	Spain	Mixed	"	"
76	Moyano and Trujillo (2014)				14.7	2010			English	Journal
	Sample 1	66	I	50	N/R	"	Spain	Islamist	"	"
	Sample 2	49	I	50	N/R	"	Spain	Mixed	"	"
77	Narraina (2013)	30190	A	49.5	46.86	2008	EU (Mixed)	Mixed	English	Thesis
78	Nivette et al. (2017)	1214	A	50	17	2015	Switzerland	Mixed	English	Journal
79	Obaidi, Bergh, et al (2018)								English	Journal
	Study 1	491	A/I	32.5	N/A	N/R	Denmark	Islamist	"	"
	Study 2	243	A/I	53.5	N/A	2013	Denmark	Islamist	"	"
80	Obaidi, Kunst, et al (2018)				N/R	N/R			English	Journal
	Study 1	152	A/I	36.2	"	"	Denmark	Islamist	"	"
	Study 2	151	A/I	42.6	"	"	Sweden	Islamist	"	"
81	Obaidi, Thomsen, et al (2018)								English	Journal
	Study 1	151	I	42.6	N/R	N/R	Sweden	Islamist	"	"
	Study 2 (Sample 1)	142	I	42.9	26.7	N/R	Demark	Islamist	"	"
	Study 2 (Sample 2)	112	I	51.8	28.67	N/R	Demark	Mixed	"	"
82	Obaidi et al. (2019)				N/R	N/R			English	Journal
	Study 1	59	I	61.02	"	"	Denmark	Islamist		
	Study 2	232	I	42.24	"	"	Denmark	Islamist	"	"
	Study 3	259	I	31.66	"	"	Denmark	Islamist	"	"
	Study 4	243	I	53.50	"	"	Denmark	Islamist	"	"
	Study 5	104	I	46.15	"	"	Belgium	Islamist	"	"
	Study 6	60	I	55.00	"	"	Demark	Islamist	"	"
83	Obaidi (2020)	366	I	49.5	24.48	N/R	Mixed	Islamist	English	Unpublished
84	Obaidi et al. (2020)					N/R			English	Unpublished
	Study 1	222	I	36	N/R	"	Denmark	Islamist	"	"
	Study 2	104	I	44	30.1	"	Belgium	Islamist	"	"
	Study 3	202	I	56	22.8	"	Sweden	Islamist	"	"
85	Okan (2017)	557	A	38.96	N/R	2017	Australia	Mixed	English	Thesis
86	Oskooii and Dana (2017)	1904	A	48	39.01	2010	UK	Islamist	English	Journal
87	Ozer (2020)	223	A	28.3	22.74	N/R	Denmark	Mixed	English	Journal
88	Ozer et al. (2019)								English	Journal
	Sample 1	225	A	40.4	28.57		UK	Islam	"	"
	Sample 2	223	A	28.3	22.74		Demark	Mixed	"	"
89	Ozer and Bertelsen (2018)					N/R			English	Journal
	Sample 1	322	A	33.2	17.93	"	Denmark	Mixed	"	"
	Sample 2	364	A	33	18.54	"	USA	Mixed	"	"
90	Ozer and Bertelsen (2019)								English	Journal
	Sample 1	322	A	33.2	17.93	"	Denmark	Mixed	"	"
	Sample 2	364	A	33	18.54	"	USA	Mixed	"	"
91	Ozer and Bertelsen (2020)	686	A	34.21	18.21	N/R	Denmark and US	Mixed	English	Journal
92	Pauwels and Heylen (2017)	723	A/B	35.7	N/A	2013	Belgium	RWE	English	Journal
93	Pauwels and De Waele (2014)	2879	A/B	35.9	N/A	2012	Belgium	Mixed	English	Journal
94	Pauwels and Schils (2016)	6020	A/B	35	20	2013	Belgium	Mixed	English	Journal
95	Pauwels and Boudry (2017)	4000	A/B	35.5	N/A	2012	Belgium	Mixed	Dutch	Chapter
96	Pedersen et al. (2017)	7659	A	45	17.05	2015	Norway	Mixed	English	Journal
97	Perliger et al. (2016)	282	B	100	N/A	2005	Mixed	Islamist	English	Journal
98	Pfundmair et al. (2019)	75	A	73.33	22.22	2017	EU (Mixed)	Islamist	English	Journal
99	Rip et al. (2012)			67.6	35	2011			English	Journal
	Study 1	114	A	65.8	33	"	Canada	Separatist	"	"
	Study 2	111	A	67.6	35	"	Canada	Islamist	"	"
100	Rousseau et al. (2016)	1241	A	32	20.02	2016	Canada	Mixed	French	Report
101	Rousseau, Hassan, et al (2019)	1190	A	30	N/R	2016	Canada	Mixed	English	Journal
102	Rousseau, Oulhote, et al (2019)	1190	A	30	N/R	2016	Canada	Mixed	English	Journal
103	Rousseau et al. (2020)	854		30	30	2015			English	Journal
	Sample 1	854	A	N/R	N/R	2015	Canada	Mixed	"	"
	Sample 2	702	A	N/R	N/R	2017	Canada	Mixed	"	"
104	Schbley and McCauley (2005)	650	A	N/R	N/R	2002	EU (Mixed)	Islamist	English	Journal
105	Schmuck and Tribastone (2020)	143	A	37	23.35	2018	Austria	Islamist	English	Journal
106	Schumpe et al. (2018)					N/R			English	Journal
	Study 1	200	A	59.5	34.33	"	US	LWE	"	"
	Study 2	111	A	51.35135	34.59	"	US	LWE	"	"
107	Schumpe et al. (2020)								English	Journal
	Study 1	460	A	36.30	31.45	N/R	Spain	Mixed	"	"
	Study 2	371	A	26.77	27.89	2017	Spain	Mixed	"	"
	Study 3	121	A	47.11	31.73	N/R	Mixed	Mixed	"	"
	Study 4	305	A	51.15	34.71	"	Mixed	Mixed	"	"
	Study 5	234	A	47.44	33.56	"	Mixed	LWE	"	"
	Study 6	160	A	45.63	48.26	"	Mixed	LWE	"	"
	Study 7	245	A	45.31	36.38	"	Mixed	LWE	"	"
	Study 8	174	A	48.85	52.26	"	Mixed	LWE	"	"
	Study 9	392	A	75.77	36.24	"	Mixed	LWE	"	"
108	Simon et al. (2013)	341	A	33	24	2010	Germany	Mixed	English	Journal
109	Stankov, Higgins, et al. (2010)	452	A	52.51	18.35		Mixed	Mixed	English	Journal
110	Stankov, Saucier, et al (2010)	2424	A	35.85	21		Mixed	Mixed	English	Journal
111	Storm et al. (2020)								English	Report
	Study 1	4926	A	0.47	23.75	2017	EU (Mixed)	Mixed	"	"
	Study 2	27936	A	0.45	54.88	2017	EU (Mixed)	Mixed	"	"
	Study 3	8583	A	50	21	2013	EU (Mixed)	Mixed	"	"
	Study 4	14491	A	47	17	2015	Norway	Mixed	"	"
112	Szlachter et al. (2012)	534	A	44.9	35.19	2010	Poland	Mixed	English	Journal
113	Tahir et al. (2019)								English	Journal
	Study 1	253	I	47.8	32.49	2018	Norway	Islamist	"	"
	Study 2	194	I	48.2	37.13	2018	UK	Islamist	"	"
114	Tausch et al. (2009)	1000	A	50	37.55	2006	UK	Islamist	English	Journal
115	Tausch et al. (2011)	466	A	46.1	26.69	2010	UK	Islamist	English	Journal
116	Travaglino and Moon (2020)					N/R			English	Journal
	Study 1	601	I	59.23	38.36	"	South Korea	Mixed	"	"
	Study 2	613	I	55.95	33.39	"	US	Mixed	"	"
	Study 3	120	I	45.83	22.3	"	South Korea	Mixed	"	"
	Study 4	151	I	51.66	34.85	"	US	Mixed	"	"
	Study 5	160	I	48.13	33.2	"	US	Mixed	"	"
117	Troian et al. (2019)					N/R			English	Journal
	Study 1	341	I	14.4	20.33	"	France	Mixed	"	"
	Study 2	269	I	26.4	21.83	"	Belgium	Mixed	"	"
	Study 3	279	I	12.5	19.14	"	France	Mixed	"	"
118	Trujillo et al. (2016)								English	Journal
	Study 1	514	I	44.6	35.3	2015	Spain	Mixed	"	"
	Study 2	133	I	43.6	29.05	2015	Spain	Mixed	"	"
119	van Bergen et al. (2015)					2012			English	Journal
	Sample 1	166	A/I	47	15.7	"	The Netherlands	Islamist	"	"
	Sample 2	232	A/I	47	15.86	"	The Netherlands	Islamist	"	"
120	van Bergen et al. (2016)	133	A/I	63.8	15.58	2011	The Netherlands	Mixed	English	Journal
121	Van den Bos et al. (2009)			61	17	2008			Dutch	Report
	Islamist model	131	A/I	N/A	N/A		The Netherlands	Islamist	"	"
	Right‐wing model	1210	A/I	N/A	N/A		The Netherlands	RWE	"	"
	Left‐wing model	1341	A/I	N/A	N/A		The Netherlands	LWE	"	"
122	van der Veen (2016)					2015			English	Thesis
	Study 1	179	A	54	37.6		US	Islamist	"	"
	Study 2	183	A	47	27.2		The Netherlands	Islamist	"	"
123	Vergani et al. (2019)	148	A	37.7	21.6	2017	Australia	Mixed	English	Journal
124	Victoroff et al. (2012)								English	Journal
	EU model	1627	A	54	35	2007	EU (Mixed)	Islamist	"	"
	US model	1050	A	53	42.37	2006	US	Islamist	"	"
125	Wojcieszak (2010)	114	A	86	33	2005	US	RWE	English	Journal
126	Zaidise et al. (2007)	1002	A	48	N/A	2001	Israel	Islam/RWE	English	Journal
127	Zhirkov et al. (2014)	1627	A	58	34	2006	EU (Mixed)	Islam	English	Journal

Abbreviaations: LWE, left‐wing; N/A, not available; RWE, right‐wing.

##### Participants

The size of the samples of included studies varied significantly, ranging from 46 to 41,828, with an average sample size was 1685 (*SD* = 4896.94) and the median was 384. The total number of participants in the included studies (without accounting for overlapping samples) was 350,577. The average age of participants across the different samples ranged from 14.6 to 54.88, with an average mean age of 29.42. The gender composition of the samples ranged from samples with a proportion of males ranging from as little as 9.1% to samples made up entirely of males (100%). The average proportion of males in samples was 49.35%.

##### Outcomes

The studies primarily reported on the outcome of Radical attitudes in the form of assessing support for or justification of radical violence, terrorism, or radical groups (*N* = 108). All of these were based on self‐reports with the exception of a single clinician reported study. Radical intentions were examined in *N* = 61 of the samples by way of assessing intentions towards, or a willingness to engage in radical violence. Radical behaviors were examined in *N* = 12 studies, including *N* = 5 that compared terrorists with the general population, and *N* = 7 that compared terrorists with nonviolent radicals. A number of samples also reported on multiple outcomes, including: Radical intentions and radical attitudes (*N* = 15), Radical attitudes and self‐reported radical violence (*N* = 7), Radical intentions and self‐reported radical violence (*N* = 2), and one study that reported on all three outcomes.

##### Settings

These 206 samples were derived from 20 eligible countries, as well as samples made up from a combination of these countries, namely: Australia (4), Austria (1), Belgium (9), Belgium and Canada combined (2), Canada (12), Denmark (17), Denmark and United States combined (1), France (8), Germany (11), Greece (1), Hungary (1), Israel (2), Italy (1), Mixed European Union countries (10), Mixed OECD countries (12), the Netherlands (12), Norway (4), Poland (2), South Korea (2), Spain (15), Sweden (5), Switzerland (4), United Kingdom (15), United States (52), and United States and Canada combined (3).

##### Ideology

The 206 samples examined a spectrum of radicalizing ideologies. The majority of the samples (*N* = 96) examined no specific ideological strain or a mixture of ideological strains. A large number of the studies (*N* = 75) examined Islamist ideological strains or were otherwise based on samples made up entirely of Muslims. Another 23 samples examined Left‐wing ideological strain, and 9 samples specifically addressed Right‐wing ideological strain. Two of the samples pertained to ethno‐nationalist ideological strains, and one study to a separatist ideological strain. One sample examined both Islamist and Right‐wing ideological strains, and another examined.

##### Design

The majority of the samples were based on cross‐sectional designs (*N* = 186). However, some samples were derived from longitudinal data (*N* = 9). Additionally, 10 case‐control designs were included, 9 of which compared violent radicals with either nonviolent radicals and the general population, and 1 of which compared clinically defined nonviolent radicals with those defined as nonradicals.

##### Publication status

The majority of the included studies were published in peer‐reviewed journals (*N* = 104). However, a number of thesis papers (*N* = 10), reports (*N* = 9), book chapters (*N* = 5), and prepublication manuscripts (*N* = 3) were included. Most were published in English, however there were also publications in German (*N* = 4), Dutch (*N* = 2), French (*N* = 1), and Spanish (*N* =  1).

#### 6.1.3 Excluded studies

In the full‐text screening stage, almost half of the studies were excluded for a variety of reasons. A total of 86 of the excluded studies pertained to the topic and were conducted in eligible countries. However, 58 of them were found to have examined ineligible outcomes with measures of radicalization that fell outside of the inclusion criteria. Further, 76 of the excluded studies did not meet the inclusion criteria pertaining to methodology. This was generally due to the lack of variation on the dependent variable (e.g., samples made up exclusively of terrorists).

Additionally, as described above, some of the studies that were included reported on multiple individual studies. In a few cases, one or more of these individual studies failed to meet the inclusion criteria.

### 6.2 Risk of bias in included studies

Table 13 details the risk of bias assessment for the included studies.

#### 6.2.1 Replicability

In 42 cases a sample's mean age was not reported and we were unable to identify it from other sources. Similarly, for *N* = 18 cases, the proportion of males in a sample was not reported and we were unable to identify it from other sources. Additionally, in 24 cases, the year of data collection for a sample was not reported and we were unable to identify it from other sources.

There were 53 samples which were identifiable as overlapping with other samples from included items. There were an additional nine cases in which we suspected that a sample was overlapping with one from another study, however we were unable to confirm this due to missing descriptive data.

#### 6.2.2 Inclusion criteria and sampling method

Most of the studies provided no specific criteria for inclusion or exclusion. Rather, most studies sought to provide random or representative samples (see Table S1). In terms of sampling methods, most studies appear to have used appropriate sampling methods. While most studies reported the sampling method, 19 studies did not. However, for nine of these studies, it was possible to deduce the method based on other descriptions within the study. For 10 of the samples it was not possible to deduce the method with any level of confidence. For studies that reported the sampling method, or for which it was possible to deduce the methods, a variety of methods were found, including: Accidental (*N* = 4), Convenience (*N* = 17), Convenience/Snowball (*N* = 1), Disproportionate stratified random (*N* = 4), Probability (*N* = 2), Proportional quota (*N* = 3), Purposive nonrandom (*N* = 3), Purposive random (*N* = 59), Quota (*N* = 7), Random (*N* = 66), Representative (*N* = 22), Snowball (*N* = 7), and Stratified random (*N* = 2).

For the most part, included items and their samples were based off of original data (*N* = 168). An additional 3 studies combined original and secondary data, in the case of case‐control studies for example. This meant that 35 studies used secondary data.

#### 6.2.3 Use of validated measures

While 104 of the samples used validated measures of “radicalization,” 102 of the samples used nonvalidated measures. However, among those that did not use validated measured were a large number of studies that used single item measurements assessing support for or justification of terrorism, and suicide bombings specifically. While imperfect, such measures are considered to be strong proxies for Radical attitudes in the absence of more validated measures (Schmid, 2017). Additionally, a number of the samples with nonvalidated outcome measures examined Radical intentions, and used measures that have been widely used in the literature. With regard to independent variables, the majority of the samples used validated measured (*N* = 171) and only a minority of studies used nonvalidated measured (*N* = 35) items. However, even in cases of nonvalidated measures, single and multiple survey items were found to be close to the constructs captured by more validated measures.

Most studies described independent and dependent variables well enough to facilitate replication. Many studies included supplementary materials.

#### 6.2.4 Overlapping data

As noted above, we identified overlapping data used in 54/206 samples, and overlapping data was suspected between an additional 9 samples. While overlapping data was more common in studies using secondary data, overlapping data in studies utilizing original data was also identified. Whilst some of these studies were explicit about the use of data in prior studies, and even cited these studies, for other studies the overlaps were identified by the research team by comparing sample characteristics. For some studies we continued to suspect overlapping data, although differences in a study level characteristic (such as a sample's mean age) meant that we were unable to confirm these suspicions.

For the most part, there was little overlap in the factors analysed in these studies. For studies using secondary data, the most common overlaps were for the factors of Gender, Age and Education. For studies using original data, there were only a small number of cases of reporting of effect sizes for the same factor. In all such cases we pooled the studies in an internal meta‐analysis and used the pooled estimate as the input.

For studies with suspected overlaps, we took the same approach (carrying out an internal meta‐ analysis and used the pooled estimate as the supplementary effect sizes) and assessed whether this had any effect on the estimates. As the largest change in effect size for any of the six factors for which this approach was used was.01, and there were no significant effects on heterogeneity, we present the results from the analyses that included the independent effect sizes. In all cases there were no more than 2 effect sizes from samples suspected of overlap.

#### 6.2.5 Reporting on nonsignificant findings

A number of the included studies reported that some factors were found not to be statistically significant, but did not report any information from which an effect size could have been calculated. While a number of studies noted that they controlled for key sociodemographic variables, these were not reported in a number of studies. Missing data was successfully retrieved from online supplementary materials for 21 studies, from the re‐processing of original data for 6 studies, and directly from authors for eight studies.

For 10 factors from two studies it was still not possible to obtain the missing information needed for the calculation of effect sizes which the documents merely reported as not having been statistically significant (Moskalenko & McCauley, 2009; Pfundmair et al., 2019). In one case a minimum p value was reported and effect sizes were estimated from this. In the other case we took the standard approach in which we ran the analysis without the missing study, and then compared it with an analysis in which the study's effect size was imputed as zero. While this approach may lead to underestimation of the magnitude of the estimate (Durlak & Lipsey, 1991), it is considered to be preferable over exclusion of the effect size and the possible overestimation of the magnitude of the estimate (Rosenthal, 1995).

#### 6.2.6 Analysis of factors as described

For some studies, while sociodemographic and other factors were reported as having been included in analyses, their effects were not reported. For some studies, additional effects were identifiable in supplementary materials, whereas for others such materials were not available. In some cases, we were able to ascertain the data directly from the authors, or from accessing the publicly available data used in the studies (k = 6).

#### 6.2.7 Nature of the data

While the majority of studies provided sufficient data for the calculation of bivariate relationships between individual factors and at least one of the outcomes of interest, some studies only provided data for calculating partial effect sizes. As with observational studies more generally, it is necessary to consider the potential for confounding. While the correlations appear to operate in the theorized direction, the data does not provide the ability to derive causal inferences. Caution must therefore be taken in interpreting the results for the purposes of policy. While correlations may be useful for the purposes of risk assessment, they do not provide sufficient evidence that changes in these factors will lead to changes in radicalization outcomes, as in the case of interventions (Murray et al., 2009).

With the exception of the longitudinal studies, most studies did not examine factors before the onset of the outcome. Even in the case of longitudinal studies, which primarily used ordinal or continuous measures of Radical attitudes or intentions, the time and variation between Time 1 and Time 2 measures was small, indicating that they too were not measuring factors before the onset of the outcome. However, for some factors, survey items were constructed retrospectively, seemingly to a period that is likely to have preceded the onset of the outcome, such as in the case of abuse or exposure to violence in adolescence. However, for case‐control studies assessing radical behaviors, many of the factors were measured as having occurred or having been present prior to the onset of the outcome.

### 6.3 Synthesis of results

The results of the meta‐analysis include a range of factors. Some factors, such as gender, marital status, immigrant status, and employment status, welfare recipient, criminal history etc. represent dichotomous constructs. Other factors such as education, socioeconomic status (SES), are ordinal factors which also do not require elaboration. For the most part however, the factors analysed in this review represent experiential, psychological/personality traits, and attitudinal constructs that were measured using different discrete scales. As the estimates for these different factors are present below in a series of rank‐order tables as they pertain to each of the outcomes, Table 4 first provides a description of these factors.

In the following sections we present the results for the meta‐analysis in three separate sections corresponding to the three outcomes of radical attitudes, intentions and behaviors. Each section begins with a summary of the rank order of pooled estimates. To simplify comparisons and interpretations, we follow Hopkins (2002) extension of Cohen (1988) and categorize factors into tiers corresponding to “Very small” (*r* = .0‐.10), “Small” (*r* = .10‐30), “Moderate” (*r* = .30‐.50), and “Large” (*r* = .50‐.70) estimates.^1^ A color coded system is used to represent these divisions in order to provide a greater level of clarity. This approach is only for the sake of clarity and comparison. Small and even very small or “trivial” effect sizes can still have real world importance (Lipsey & Wilson, 2001).

#### 6.3.1 Radical attitudes

The outcome of radical attitudes included studies whose outcomes assessed justification or support for the use of violent radical behaviors, of groups engaged in such behaviors, or of specific events of radical behaviors. The analysis was based on 838 effect sizes pooled across 100 factors, made up of 29 protective factors derived from 234 effect sizes, and 71 risk factors derived from 604 effect sizes. The identified risk factors span all of the domains of 1) Sociodemographic and background factors, 2) Psychological and personality trait factors, 3) Attitudinal and subjective belief related factors, and 4) Experiential factors, and 5) “Traditional criminogenic and criminotrophic factors.” The results are arranged in a color coded rank‐order according to the size of the estimates for the different factors. The general findings indicate that the smallest estimates are related to sociodemographic and background characteristics, whereas the largest estimates tend to be associated with the domains of psychological and personality trait factors and traditional criminogenic factors (Table 5).

**Table 4:** Description of risk and protective factors analysed

Factors	Example descriptions
*Adjusted personality disorder (APD)*	For example, DSM‐IV personality disorders, Narcissistic Personality Inventory
*Aggression*	For example, Buss‐Perry Aggression Questionnaire (BPAQ‐SF)
*Agreeableness*	Big‐five construct: Cooperation/social harmony
*Alcohol use*	The extent to which the individual consumes alcohol
*Anger/hate*	Angry, resentful, furious, or displeased with a given issue or situation
*Anomia*	Social alienation
*Anti‐democratic attitudes*	Negative attitudes towards democratic norms
*Anxiety*	For example, General Anxiety Disorder Scale (GAD‐7),
*Authoritarianism/fundamentalism*	Submission to higher authority/aggression to out‐groups
*Bullied*	Victim of bullying during adolescence
*Collective relative deprivation*	In‐group is deprived or discriminated against relative to other groups
*Commitment*	Level of commitment to a cause
*Conscientiousness*	Big five construct: efficient/organized
*Criminal history*	Has a criminal record for unspecified offences
*Dark‐triad*	Narcissism/Machiavellianism/Psychopathy (Dark world view)
*Dehumanization*	The attitude that a person or group lacks good/human qualities
*Depression*	For example, Patient Health Questionnaire (PHQ‐9)
*Deviant peers*	Peers support/involved in deviance, including radicalism
*Discrimination*	Experienced personal discrimination based on identity
*Disconnectedness*	Lack of social contacts/activity
*Drug use*	The extent to which the individual uses illicit substances
*Dual identity*	Torn between more than one important group‐based identity
*Education*	Highest level of education attained
*Experienced violence*	Perpetrated/victim of violence involving strangers, bullies, or parents
*Extraversion*	Big five construct: outgoing
*Family violence*	Degree of violence occurring within the family unit
*Fear of crime*	Fear of falling victim to crime
*General trust*	Trusting of others
*Group superiority*	Believing that one's in‐group is better than other groups
*Harmonious passion*	Passion for an activity as an integrated aspect of one's life
*Immigrant status*	The individual is a first or second generation immigrant
*Individual relative deprivation*	Feeling unfairly treated compared to others
*In‐group identity*	Identity is based on a group identity (e.g., religious/ethnic/national)
*Institutional trust*	Confidence in institutions (e.g., police, parliament, courts, etc.)
*Integration*	Degree of attachment to the society in which one lives
*Job loss*	Recent loss of employment
*Juvenile delinquency*	Involvement in norm/law breaking before the age of 18
*Law abidance*	There is a duty to follow and abide by the law
*Law legitimacy*	Respect for the government/law/authorities
*Legal cynicism*	Believing that laws are made to be broken
*Life events*	Social experience or change with psychological effects
*Life satisfaction*	Evaluation of quality of life
*Machoism*	Exaggerated masculinity
*Marital status*	Single/married
*Mental health*	Aggregate measure of undefined mental‐health conditions
*Military service*	Current/past service in military
*Moral neutralizations*	Justifications of deviant behaviors (e.g., drugs, violence etc.)
*Need for closure*	Need to reach conclusion/aversion toward ambiguity
*Negative affect*	Negative emotions (Positive and Negative Affect Schedule (PANAS))
*NSM contact*	Contact with other radicals via new social media (NSM)
*Obsessive passion*	Passion for an activity that is consuming of one's life or identity
*Online posting*	Active posting of political/radical opinions/content online
*Openness*	Big five construct: Inventiveness/curiousness
*Out‐group friendships*	Friendships with members of out‐groups
*Parental abuse*	Physically abused by parents
*Parental academics*	Parental academic level achieved
*Parental control*	Degree of parental supervision exercised
*Parental involvement*	Parents show interest, praise, and are aware of whereabouts
*Past activism*	Engaged in legal, nonviolent behaviors in the name of a cause
*Perceived discrimination*	Perception of having been discriminated against
*Perceived injustice*	Feeling that the individual or group is treated unjustly
*Personal strain*	Loss of parents, loss of work, experienced traumatic event etc.
*Personal trust*	Believing that most people can be trusted
*Physical health*	General/overall health status
*Police contact*	Number of contacts with police in the previous 12‐month period
*Political efficacy*	Having influence or being represented in the political sphere
*Political extremism*	Far left/right politically
*Political grievance*	Opposition to foreign intervention in the Middle East
*Political participation*	Participation in political party, organization, activities
*Political satisfaction*	Satisfaction with current system of government
*Positive affect*	Positive emotions (Positive and Negative Affect Schedule (PANAS))
*Power Distance Orientation (PDO)*	Acceptance of inequality between classes/hierarchical structures
*Prayer frequency*	Frequency of individual prayers
*Previous incarcerations*	Number of previous incarcerations for unspecified offences
*Procedural justice*	Treated (un)fairly by legal institutions
*Psychopathy*	Dark triad construct: Impaired empathy
*PTSD*	Post‐traumatic stress disorder/trauma
*Radical attitudes*	Support for or justification of radical violence in the name of a cause
*Radical family*	Family members with cognitive or behavioral radicalization
*Radical media*	Passive exposure to mediated, radical content
*Religious upbringing*	Raised in a practicing, religious home
*Quest for significance*	Seeking to attain/regain lost or absent personal significance
*Realistic threat*	Powerful out‐group threatening to in‐group survival
*Religiosity*	Importance of religion in daily life and activities
*School bonding*	Enjoying going to school and/or studying/attachment to school
*Segregationist*	Separation of people by ethnic group
*Self‐control*	Impulsivity, quick to anger
*Self‐efficacy*	Belief/confidence in capacity to achieve objectives
*Self‐esteem (individual/public/group)*	Self/public/group value
*Self‐sacrifice*	A willingness to sacrifice on behalf of group/cause
*Similar peers*	Proportion of friends of similar background
*Social Dominance Orientation (SDO)*	Desire for social dominance over others
*Social support*	Perceived/feelings of receiving adequate support from others
*Socioeconomic status (SES)*	Level of personal/household income
*Student*	The individual is a current student
*Symbolic threat*	Out‐group's influence threatening to in‐group's position
*Teacher mistreatment*	Feelings of being mistreated by educators
*Thrill‐seeking/risk‐taking*	Taking risks just for fun of it, without thinking of consequences
*Time online*	The number of hours spent on the internet
*Uncertainty*	Anxiety prior to confronting potentially harmful events
*Unemployment*	Lack of gainful employment
*Violent media exposure*	Passive exposure to mediated violence/violent content
*West Vs. Islam*	The West is trying to attack/dominate Islam/Islamic countries
*Worship attendance*	The frequency of attendance at places of worship

**Table 5:** Risk factors for radical attitudes

	Factor	*r*	95% CI	*Q*	*I* ^*2*^	*τ* ^2^	*N* (*k*)
	*Protective factors*						
V. small	Student	0	−0.11, .11	42.74***	90.64	0.014	3484 (5)
	Neuroticism	−.02	−0.10, 0.07	30.18***	90.06	0.007	6156 (4)
	Children	−.02***	−0.03, −0.01	0.04	0	0	31,984 (2)
	SES	−.04**	−0.06, −0.01	244.65***	89.37	0.002	110,617 (27)
	Marital status	−.04***	−0.06, −0.02	12.8	14.03	0	37,105 (11)
	Political efficacy	−.05	−0.13, 0.04	130.40***	95.34	0.012	14,305 (7)
	Age	−.05**	−0.08, −0.02	1307.75***	96.56	0.009	151,045 (46)
	General trust	−.06**	−0.11, −0.02	8.21*	63.44	0.001	32,546 (4)
	Education	−.07**	−0.10, −0.03	568.90***	94.9	0.009	72,528 (30)
	Procedural justice	−.08	−0.21, 0.05	3.99*	74.9	0.007	1147 (2)
	School performance	−.09**	−0.14, −0.04	155.57***	95.5	0.005	43,740 (8)
	Outgroup friends	−.09**	−0.14, −0.04	1.081	0	0	1398 (3)
	Extraversion	−.1	−0.29, 0.10	164.78***	98.18	0.04	6156 (4)
	Parental academics	−.10***	−0.14, −0.05	28.24***	89.38	0.002	27,736 (4)
	Openness	−.10†	−0.21, 0.01	47.94***	93.74	0.011	6156 (4)
	Parent involvement	−.10***	−0.15, −0.06	121.47***	90.94	0.005	26,175 (12)
Small	Self‐esteem (public)	−.11	−0.26, 0.04	13.27**	84.93	0.016	1801 (3)
	Self‐esteem (group)	−.11	−0.27, 0.06	15.47***	87.07	0.019	1801 (3)
	Social support	−.12***	−0.15, −0.08	19.07**	68.53	0.002	12,223 (7)
	Conscientiousness	−.12***	−0.15, −0.09	3.46	13.18	0	6156 (4)
	Parental control	−.12**	−0.20, −0.04	65.28***	95.4	0.006	15,647 (4)
	School bonding	−.13***	−0.16, −0.10	24.62***	75.63	0.001	22,174 (7)
	Teacher bonding	−.13***	−0.18, −0.08	7.62*	73.75	0.001	9105 (3)
	Agreeableness	−.13*	−0.23, −0.03	40.70***	92.63	0.009	6156 (4)
	Political satisfaction	−.15**	−0.26, −0.04	349.35***	98.57	0.018	41,665 (6)
	Self‐esteem (Indiv.)	−.17**	−0.29, −0.05	8.43*	76.27	0.009	1801 (3)
	Institutional trust	−.17***	−0.27, −0.07	822.18***	98.78	0.03	47,485 (11)
	Life satisfaction	−.19***	−0.22, −0.15	1.46	0	0	2638 (3)
Lrg.	Law abidance	−.55***	−0.64, −0.45	68.48***	97.08	0.015	8606 (3)
	*Risk factors*						
V. small	Depression	.00	−0.07, 0.07	87.27***	89.69	.011	9027 (10)
	Immigrant	.01	−0.02, 0.04	183.06***	87.98	.003	63,157 (23)
	2nd Gen. immigrant	.01	−0.02, 0.04	142.02***	92.96	.002	85,719 (11)
	Political participate	.01	−0.03, 0.06	6.87†	56.31	.001	5258 (4)
	Life events	.02	−0.02, 0.06	46.08***	82.64	.003	18,928 (9)
	Fear of crime	.02	−0.05, 0.10	4.51*	77.81	.002	7146 (2)
	Physical health	.02*	0.00, 0.04	2.19	0.00	.000	9713 (4)
	Need for closure	.03	−0.05, 0.10	.247	0.00	.000	703 (2)
	Moved residence	.03	−0.01, 0.08	16.94**	82.29	.001	15,735 (4)
	Anxiety	.04†	−0.00, 0.08	23.90*	70.71	.002	10,409 (8)
	APD	.03	−0.07, 0.12	14.961*	79.95	.006	4840 (4)
	Alcohol use	.04†	−0.01, 0.09	22.13***	81.92	.002	11,916 (5)
	Religiosity	.05	−0.02, 0.11	504.99***	96.44	.018	30,978 (19)
	West vs. Islam	.05***	0.02, 0.07	.637	0.00	.000	5985 (3)
	Unemployed	.05***	0.03, 0.07	26.27*	54.31	.000	52,596 (13)
	Welfare	.05***	0.03, 0.07	17.86*	55.20	.000	26,304 (9)
	Worship attendance	.06*	0.01, 0.11	103.10***	88.36	.006	16,761 (13)
	Time online	.06*	0.00, 0.12	1.77	43.55	.001	7039 (2)
	Uncertainty	.07**	0.02, 0.11	37.76***	76.17	.003	20,960 (10)
	In‐group identity	.07***	0.038, 0.11	344.11	93.03	.006	77,618(25)
	Exp. violence	.07***	0.05, 0.10	97.78***	84.66	.002	65,566 (16)
	Perceived injustice	.08*	0.01, 0.14	46.73***	85.02	.007	7279 (8)
	Exp. discrimination	.08***	0.06, 0.10	74.80***	73.26	.001	47,670 (21)
	Teacher mistreated	.08***	0.06, 0.11	.689	0.00	.000	6803 (2)
	Aggression	.09**	0.03, 0.16	31.99***	87.50	.004	12,555 (5)
	Family violence	.10***	0.06, 0.13	12.84*	68.86	.001	15,923 (5)
	Males	.10***	0.08, 0.12	631.10***	91.29	.004	176,203 (56)
Small	Negative affect	.11†	−0.00, 0.22	.067	0.00	.000	311 (2)
	Online posting	.11***	0.05, 0.17	20.72***	85.52	.003	12,715 (4)
	Indiv. relative Dep.	.11***	0.06, 0.16	379.35***	96.31	.008	62,987 (15)
	Drug use	.12*	0.00, 0.22	146.25***	96.58	.017	11,991 (6)
	Violent media	.12***	0.07, 0.17	79.98***	93.75	.003	35,615 (6)
	Personal strain	.13***	0.08, 0.17	.408	0.00	.000	1733 (3)
	Parental abuse	.13***	0.10, 0.17	21.13**	76.33	.001	17,711 (6)
	Self‐efficacy	.13	−0.03, 0.29	306.96***	98.70	.036	12,348 (5)
	Anger	.14***	0.07, 0.20	6.69†	55.18	.002	3475 (4)
	Prayer frequency	.14**	0.05, 0.23	36.56***	89.06	.009	6159 (5)
	Significance quest	.14***	0.08, 0.21	19.60*	54.08	.006	2165 (10)
	Perc. discrimination	.15***	0.10, 0.19	73.87***	90.52	.004	20,093 (8)
	Religious convert	.15***	0.08, 0.21	.128	0.00	.000	904 (2)
	Political grievance	.15***	0.08, 0.21	52.62***	86.70	.007	7990 (8)
	Dual identity	.15***	0.09, 0.21	36.55***	83.59	.005	10,140 (7)
	Segregationist	.15†	−0.03, 0.32	27.93***	92.84	.022	2437 (3)
	Collect. Rel. Dep.	.16***	0.12, 0.19	199.80***	90.99	.006	34,041 (19)
	Disconnectedness	.16***	0.08, 0.23	13.84*	56.65	.005	2168 (7)
	Deviant peers	.17***	0.09, 0.25	542.85***	97.97	.020	38,006 (12)
	Psychopathy	.19	−0.10, 0.47	36.88***	97.29	.040	2981 (2)
	Antidemocratic	.19***	0.14, 0.23	43.05***	83.74	.003	14,054 (8)
	Anomia	.19***	0.14, 0.24	105.31***	89.56	.006	19,938 (12)
	SDO	.19†	−0.04, 0.40	126.07***	96.83	.063	4152 (5)
	Juv. delinquent	.20***	0.11, 0.28	212.03***	97.17	.014	18,827 (7)
	Low integration	.20***	0.15, 0.25	321.80***	93.79	.013	42,783 (21)
	Self‐sacrifice	.20***	0.09, 0.30	24.59***	79.66	.015	1704 (6)
	Legitimacy	.22***	0.15, 0.29	357.45***	97.48	.013	47,847 (10)
	PTSD	.23**	0.06, 0.38	6.73**	85.15	.013	932 (2)
	Positive affect	.24***	0.14, 0.35	.031	0.00	.000	311 (2)
	Low self‐control	.25***	0.20, 0.29	67.14***	89.57	.004	19,489 (8)
	Authoritarianism	.25***	0.15, 0.35	1962.24***	98.98	.057	37,313 (21)
	Radical media	.26***	0.19, 0.33	73.81***	94.58	.006	15,316 (5)
	Criminal history	.29***	0.18, 0.40	26.75***	88.78	.011	4976 (4)
Moderate	Police contact	.30***	0.20, 0.39	129.54***	96.14	.017	10,882 (6)
	Thrill‐seeking	.31***	0.24, 0.37	594.10***	97.31	.022	37,733 (17)
	Symbolic threat	.31***	0.24, 0.37	10.75†	53.47	.004	2341 (6)
	Similar peers	.31***	0.18, 0.43	17.87***	88.81	.012	7261 (3)
	Moral neutralization	.32***	0.23, 0.40	1119.20***	98.75	.033	52,498 (15)
	In‐group superior	.34***	0.25, 0.42	321.51***	96.27	.029	14,015 (13)
	Realistic threat	.35***	0.26, 0.44	7.41†	59.51	.006	1561 (4)
	Political extremism	.37***	0.22, 0.51	653.44***	99.24	.045	38,745 (6)
	Life attachment	.41**	0.12, 0.63	46.67***	95.71	.072	1134 (3)
	Machoism	.42***	0.34, 0.49	88.53***	96.61	.009	14,871 (4)
	Dehumanization	.43*	0.01, 0.72	12.46***	91.98	.098	394 (2)

*Note:* Effect sizes are *r* correlations, ***<.001, **<.01, *<.05. 95% confidence intervals are presented as lower and upper. Heterogeneity statistics include Cochran's *Q* statistic for heterogeneity with *p* value from associated *χ* ^2^ test, and *I* ^2^ and *τ* ^2^ statistics for the proportion and extent of variation across studies attributed to heterogeneity. *N* = combined sample size and *k* = number of effect sizes.

The protective factors for radical attitudes had estimates ranging from ‐0.00 to ‐0.19, with a single outlier estimate of ‐0.55. The estimates for Neuroticism, Extraversion, Student, Political Efficacy, Procedural Justice, as well as Public and Group Self‐Esteem were not statistically significant (*p* > .10). The estimate for Openness was only marginally significant (*p* < .10). Among the statistically significant factors, sociodemographic characteristics made up the bulk of the tier of very small estimates (*r* = .00‐.10), with factors such as: Children, Socioeconomic status, Marital status, Age, Education and parental education level. Other subjective attitude and experiential factors also featured in this tier, namely general trust, school performance, and out‐group friendships. The tier of factors with small estimates (*r* = .10‐.30) was made up of a combination of psychological and social factors, namely: Parental Involvement, Social Support, Conscientiousness, Parental Control, School and Teacher Bonding, Agreeableness, Political Satisfaction, Personal Self‐Esteem, Institutional Trust, and Life Satisfaction. A single outlier estimate of *r* = ‐.55 was found for Law Abidance.

The risk factors for radical attitudes had estimates ranging from *r* = .00 ‐.43. The estimates for 13 factors were statistically nonsignificant, namely: Depression, Immigrant Status, Political Participations, Fear of Crime, Need‐for‐Closure, Life Events, Moved House, Adjusted Personality Disorder/Narcissistic Personality Disorder (APD), Religiosity, Self‐Efficacy, Psychopathy, Social Dominance Orientation (SDO). Four more factors were found to be only marginally significant (*p* < .10), namely; Anxiety, Alcohol Use, Drug Use, and Negative Affect. Among factors with very small (*r* = .00‐.10) but statistically significant estimates, were a combination of sociodemographic, psychological, attitudinal, and experiential factors, namely: Physical health, belief in a battle between the West Vs. Islam, Unemployment, Welfare Recipient, Frequency of Attendance at Places of Worship, Time spent online, Uncertainty, In‐Group Identity, Perceived Injustice, Experiences of Discrimination, Teacher Mistreatment, Experiences of Violence, Aggression, and Experiences of Family Violence. The tier consisting of small estimates (*r* = .100–.300) featured factors from the entire spectrum of factor domains, namely: Gender (male), Posting of political/radical content online, Individual relative deprivation, Exposure to violent media, Personal strains, Victim of parental abuse, Anger/Hate, Quest for significance, Prayer frequency, Identity fusion, Perceived discrimination, Political grievances, Segregationist attitudes, Collective relative deprivation, Societal disconnectedness, Deviant peers, Anti‐democratic attitudes, Anomia, Juvenile Delinquency, Willingness to Self‐Sacrifice, Low Integration, Low Legitimacy, Post‐Traumatic Stress Disorder/Trauma (PTSD), Positive Affect, Authoritarianism/Fundamentalism, Low Self‐Control, Exposure to Radical Media and Criminal History. The tier consisting of moderate sized estimates (*r* = .30–.50) was made up of 11 factors, namely: Police Contact, Thrill‐Seeking/Risk‐Taking, Similar Peers, Symbolic Threat, Moral Neutralizations, In‐Group Superiority, Realistic Threat, Political Extremism (Right‐Left), Low Life‐Attachment, Machoism and Dehumanization.

#### 6.3.2 Radical intentions

As opposed to radical attitudes, the outcome of radical intentions included studies which assessed individuals' willingness or intentions to engage in violent radical behaviors, or to participate in the activities of groups already engaging in such behaviors. For this outcome, the analysis was based on 338 effect sizes that were pooled across 45 factors, made up of 8 protective factors derived from 79 effect sizes, and 37 risk factors derived from 259 effect sizes (Table 6).

**Table 6:** Risk and protective factors for radical intentions

	Factor	*r*	95% CI	*Q*	*I* ^*2*^	*τ* ^2^	*N* (*k*)
	*Protective factors*						
V. Small	Education	−.03	−0.08, 0.03	32.87**	66.54	0.005	5660 (12)
	SES^b^	−.03	−0.09, 0.02	21.46*	53.4	0.004	3147 (11)
	Age^a^	−.08**	−0.12, −0.03	165.75***	85.52	0.011	14,650 (25)
	Outgroup friendship	−.1	−0.15, −0.05	1.63	0	0	1398 (3)
Small	Agreeableness	−.12***	−0.14, −0.10	3.68	0	0	7668 (7)
	Conscientiousness	−.13***	−0.16, −0.10	9.87	39.21	0.001	7668 (7)
	Openness	−.16***	−0.23, −0.09	85.77***	89.51	0.011	8196 (10)
	Immigrant	−.22***	−0.38, −0.05	117.66***	94.05	0.055	3360 (8)
	*Risk factors*						
V. Small	Uncertainty	.05**	0.01, 0.08	4.51	11.21	0.000	4104 (5)
	Unemployment	.06	−0.02, 0.14	0.08	0.00	0.000	647 (2)
	Neuroticism	.07***	0.03, 0.10	15.99*	56.24	0.001	8308 (8)
	Exp. discrimination	.06**	0.03, 0.10	1.23	18.79	0.000	3278 (2)
	APD	.08**	0.02, 0.13	0.44	0.00	0.000	1364 (3)
	SDO	.09	−0.16, 0.33	118.41***	96.62	0.080	1909 (5)
	External efficacy	.09	−0.04, 0.22	19.99***	84.99	0.015	1630 (4)
	Males	.10***	0.06, 0.14	135.39***	80.06	0.008	14,806 (28)
Small	Student	.11**	0.03, 0.19	0.00	0.00	0.000	647 (2)
	Significance Quest.	.11***	0.06, 0.17	3.70	19.00	0.001	1603 (4)
	Extraversion	.12***	0.09, 0.15	13.33*	47.48	0.001	8308 (8)
	Indv. Rel. deprivation	.14†	−0.00, 0.28	14.08**	78.70	0.015	1558 (4)
	Convert	.15***	0.08, 0.21	0.191	0.00	0.000	888 (2)
	Positive affect	.16**	0.06, 0.24	5.76	47.87	0.005	786 (4)
	Harmonious passion	.16*	0.03, 0.29	16.13**	75.20	0.017	922 (5)
	Low integration	.18***	0.11, 0.26	20.88**	61.68	0.008	2318 (9)
	Self‐esteem^c^	.20*	0.00, 0.38	41.31***	87.90	0.051	1789 (6)
	Dark triad	.20**	0.07, 0.33	151.65***	96.70	0.029	6462 (6)
	PDO	.23**	0.10, 0.35	24.97***	83.98	0.018	1645 (5)
	In‐group Connect.	.23***	0.14, 0.32	12.54*	60.13	0.008	1118 (6)
	Anomia	.25***	0.13, 0.37	48.29***	89.65	0.023	2425 (6)
	In‐group identity	.25***	0.15, 0.34	212.59***	93.41	0.035	6359 (15)
	Realistic threat	.26***	0.14, 0.38	31.743***	84.25	0.022	1918 (6)
	Perceived injustice	.28***	0.16, 0.40	187.06***	93.59	0.049	4164 (13)
	Symbolic threat	.29***	0.14, 0.42	44.77***	88.83	0.033	1918 (6)
Moderate	Past activism	.33***	0.22, 0.43	4.27*	76.59	0.006	1231 (2)
	Coll. Rel. deprivation	.36***	0.26, 0.44	140.54***	88.62	0.040	3641 (17)
	Moral neutralizations	.36***	0.21, 0.50	60.39***	88.41	0.051	1235 (8)
	Perc. discrimination	.37	−0.09, 0.70	6.41*	84.40	0.102	115 (2)
	In‐group superiority	.37***	0.30, 0.45	9.09	55.99	0.005	1748 (5)
	Anger	.40***	0.27, 0.51	153.71***	92.84	0.055	3029 (12)
	Commitment	.43***	0.31, 0.54	97.97***	91.83	0.043	2545 (9)
	Activist intent	.44***	0.34, 0.53	336.24***	94.94	0.064	5446 (18)
	Negative affect	.47***	0.37, 0.56	8.89*	66.25	0.010	786 (4)
	Radical attitudes	.48***	0.38, 0.56	184.00	94.02	0.036	5917 (12)
**Lrg.**	Obsessive passion	.50***	0.33, 0.64	41.19***	90.29	0.052	922 (5)
	Identity fusion	.52***	0.45, 0.57	2.254	11.27	0.001	650 (3)

*Note:* Effect sizes are *r* correlations, ***<.001, **<.01, *<.05. 95% confidence intervals are presented as lower and upper. Heterogeneity statistics include Cochran's Q statistic for heterogeneity with p‐value from associated *χ* ^2^ test, and *I* ^2^, and *τ* ^2^ statistics for the proportion and extent of variation across studies attributed to heterogeneity. *N* = combined sample size, *k* = number of effect sizes.

^a^ Two effect sizes were imputed as zero. In removing these from the analysis there was no change in the pooled estimate.

^b^ Two effect sizes were imputer as zero. In removing these from the analysis the estimate increased to −0.04*^ns^* (95% CI = −0.11, 0.03). Heterogeneity remained similar (*Q* = 19.959**, *I* ^2^ = 59.92, *T* ^2^ = 0.006).

^c^ One effect size was imputed as zero. In removing the effect size from the analysis the estimate was increased to 0.24^†^ (95% CI = −0.01, 0.46). Heterogeneity remained high (*Q* = 40.626***, *I* ^2^ = 90.15, *T* ^2^ = 0.073).

The protective factors for radical intentions had estimates ranging from *r* =  ‐.03 to ‐.22. The estimates for Education, Socioeconomic status (SES) and Outgroup friendships were not statistically significant (p > .10). Age had the smallest of the statistically significant estimates, followed by three factors from the “Big Five” set of personality traits, namely Agreeableness, Conscientiousness and Openness (the other traits, Extraversion and Neuroticism were risk factors). The largest estimate was found for Immigrant status, where being an immigrant had a negative association with radical intentions.

The risk factors for radical intentions had estimates that ranged from *r* = .05‐.52. Statistically nonsignificant estimates were found for Unemployment, Social‐Dominance Orientation (SDO), External political efficacy, and Perceived discrimination. Very small (*r* = .00‐.10) but statistically significant estimates were found for: Uncertainty, Neuroticism, Experiencing discrimination, and Adjusted Personality Disorder/Narcissism. The next tier, consisting of small estimates (*r* = .10‐.30) included: Student, Gender (Males), Quest for significance, Extraversion, Individual relative deprivation, Religious convert, Positive affect, Harmonious passion, and Low integration, Dark‐triad personality traits, Power Distance Orientation (PDO), In‐group connectedness, Personal self‐esteem, Anomie, In‐group identity, Realistic threat, Perceived injustice and Symbolic threat. The tier consisting of moderate sized estimates (*r* = .30‐.50) included: Past activism, Collective relative deprivation, Moral neutralizations, Perceived discrimination, In‐group superiority, Anger, Commitment to a cause, Activist intentions, Negative affect, Radical attitudes. Large estimates (*r* > 50 >) were found for Obsessive Passion and Identity Fusion.

#### 6.3.3 Radical behaviors

The outcome of radical behaviors included studies assessing involvement in violent radical behaviors, including illegal and violent subterroristic behaviors motivated by a radical ideology, and behaviors that can be classified as terrorism. The analysis was based on 137 effect sizes pooled across 33 risk and protective factors, made up of 7 protective factors derived from 39 effect sizes, and 26 risk factors derived from 98 effect sizes (Table 7).

**Table 7:** Risk and protective factors for radical behaviors

	Factor	*r*	95% CI	Q	*I* ^*2*^	*τ* ^2^	*N* (*k*)
	*Radical attitudes*						
V. Small	Marital status	−.03	−0.07, 0.01	25.27***	80.21	.002	48,138 (6)
	Education	−.04	−0.12, 0.04	812.51***	98.52	.021	66,247 (13)
	Parent involvement	−.06***	−0.08, −0.03	6.23	51.82	.000	13,069 (4)
	Age^a^	−.10*	−0.21, 0.00	365.92***	98.09	.023	50,738 (8)
Small	School bonding	−.11***	−0.12,−0.09	0.95	0.00	.000	13,069 (4)
	Law legitimacy	−.17***	−0.20, −0.13	2.52	60.29	.001	7313 (2)
	Law abidance	−.22***	−0.26, −0.18	5.92	66.24	.001	8618 (3)
	*Risk factors*						
Small	Raised religious	.01	−0.04,0.05	0.50	0.00	.000	2387 (2)
	Religious convert	.01	−0.03, 0.06	1.54	35.03	.000	3344 (2)
	Bullied	.04***	0.02, 0.05	1.05	0.00	.000	10,851 (4)
	Immigrant	.05	−0.07, 0.17	145.91***	97.26	.016	13,290 (5)
	Welfare	.06*	0.01, 0.10	30.59***	83.65	.002	15,082 (6)
	Abused	.07***	0.05, 0.09	3.56	0.00	.000	10,975 (5)
	Relation problems	.08	−0.07, 0.23	21.67***	95.39	.012	3706 (2)
V. Small	Low integration	.11***	0.08, 0.13	0.17	0.00	.000	7293 (2)
	Exp. violence	.11***	0.07, 0.15	16.95**	76.40	.002	11,435 (5)
	Personal injustice	.15***	0.11, 0.19	5.05†	60.40	.001	7328 (3)
	Mental health	.16***	0.11, 0.22	1.37	26.82	.001	2517 (2)
	Radical family	.18*	0.02, 0.32	10.96**	90.88	.012	1698 (2)
	Authoritarian	.18***	0.16, 0.21	0.9	0.00	.000	7277 (2)
	Unemployed	.19***	0.06, 0.31	0647.22***	99.07	.030	54,620 (7)
	Thrill‐seeking	.19***	0.16, 0.22	19.25**	74.02	.001	18,143 (6)
	Anger	.20	−0.06, 0.43	179.63***	98.89	.051	5460 (3)
	Low self‐control	.28***	0.15, 0.39	81.98***	97.56	.013	9525 (3)
Moderate	Deviant peers	.30**	0.13, 0.46	228.43***	98.69	.035	9627 (4)
	Radical attitudes	.30***	0.19, 0.41	868.49***	98.49	.039	25,576 (11)
	Online contact	.31***	0.26, 0.35	2.24	55.40	.001	5258 (2)
	Past mlitary	.33†	−0.01, 0.60	208.62***	99.04	.093	3854 (3)
	Criminal history	.35**	0.10, 0.56	345.41***	99.13	.072	9346 (4)
	Current military	.35	−0.23, 0.75	233.36***	99.57	.188	3089 (2)
	Job loss	.37***	0.29, 0.45	13.67***	92.69	.004	8516 (2)
	Gender	.39**	0.10, 0.61	1887.18***	99.68	.171	13,641 (7)
Lrg.	Prior incarcerations	.63***	0.57, 0.67	5.54*	81.94	.003	7110 (2)

*Note:* Effect sizes are *r* correlations, ***<.001, **<.01, *<.05. 95% confidence intervals are presented as lower and upper. Heterogeneity statistics include Cochran's *Q* statistic for heterogeneity with *p* value from associated *χ* ^2^ test, and *I* ^2^ and *τ* ^2^ statistics for the proportion and extent of variation across studies attributed to heterogeneity. *N* = combined sample size and *k* = number of effect sizes.

^a^ The analysis included an effect size imputed as zero. In removing the imputed effect size the pooled estimate was ‐.12* (95% CI =  ‐.23, ‐.00), although there were no differences in heterogeneity (Q = 365.078***, I2 = 98.357).

The protective factors had estimates ranging from *r* =  ‐.03 to ‐.22. Statistically nonsignificant estimates were found for Marital status and Education. Statistically significant but very small estimates were found for Parental involvement, Age and School Bonding, and small estimates for Law Legitimacy and Law Abidance.

The estimates for the risk factors for radical behaviors ranged from *r* = .01‐.63 and covered all of the primary factor domains. Statistically nonsignificant estimates were found for Religious Upbringing, Religious Convert, Immigrant Status, Relationship Problems, Anger, and Current Military Service. Only three of the very small (*r* = .00‐.10) factors had statistically significant estimates, namely Bullying Victim, Welfare Recipient, and Parental Abuse. The tier consisting of small estimates *(r* = .10‐.30) included: Low Integration, Experiencing Violence, Personal Injustice, Mental Health, Radical Family, Authoritarianism/Fundamentalism, Unemployment, Thrill‐Seeking/Risk‐Taking, Anger and Low Self Control. The tier consisting of moderate estimates (*r* = .30‐.50) included: Deviant/Radical Peers, Radical Attitudes, Online Contact with Extremists, Past Military Service, Criminal History, Recent Job Loss, and Gender (Male). A large estimate (r > .50) was found Previous Incarcerations.

#### 6.3.4 Heterogeneity

As per the results displayed in Tables 5, 6, 7, there was a wide range of heterogeneity across the factors analysed. For radical attitudes, heterogeneity was high (*I* ^*2*^ > 75) for 67 factors, moderate (*I* ^*2*^ > 50) for 16 factors, low (*I* ^*2*^ > 25) for 1 factor, very low (*I* ^*2*^ < 25) for 2 factors, and absent (*I* ^*2*^ = 0) for 11 factors. For radical intentions, heterogeneity was found to be high (*I* ^*2*^ > 75) for 25 factors, moderate (*I* ^*2*^ > 50) for 6 factors, low (*I* ^*2*^ > 25) for 3 factors, very low (*I* ^*2*^ < 25) for 4 factors and absent (*I* ^*2*^ = 0) for 6 factors. For radical behaviors, heterogeneity was high (*I* ^*2*^ > 75) for 19 factors, moderate (*I* ^*2*^ > 50) for 6 factors, low (*I* ^*2*^ > 25) for 2 factors and absent for 6 factors.

While high heterogeneity is common in meta‐analyses of observational studies, and studies examining deviant outcomes, results for factors displaying high heterogeneity must be interpreted with a degree of caution. Estimates in the presence of high heterogeneity may not be accurate reflections of the true estimate. We therefore set out to investigate potential sources of heterogeneity using two methods, meta‐regression and moderator analysis.

Meta‐regression analyses were conducted for three key study‐level characteristics, namely the year of data collection, the average age of studies' samples, and the proportion of males in studies' samples. For each risk factor, these study level variables were regressed individually (univariate analysis). The analysis was only conducted on risk factors which had a minimum of 6 effect sizes for which information on the study level variable was available. For this reason, the number of effect sizes (k) in Table 8 may be different for each variable as they pertain to a specific risk factor. As the results demonstrate, one or more of the study‐level characteristics analysed was found to have a significant impact on the results for a number of factors.

**Table 8:** Meta‐regressions for year of data collection, mean sample age, and proportion of males in sample

Factor	*k*	*B* (*SE*)	95% CI	*p*
**Radical attitudes**				
*2nd gen. immigrant*				
Year of data collection	11	−0.001 (0.006)	−0.013, 0.011	.853
Mean sample age	8	−0.000 (0.002)	−0.004, 0.003	.820
% Males in sample	10	0.000 (0.003)	−0.005, 0.006	.898
*Age*				
Year of data collection	43	0.0038 (0.0040)	−0.005, 0.009	.618
Mean sample age	40	−0.004 (0.001)	−0.006, −0.002	.001
% Males in sample	43	−0.000 (0.001)	−0.003, 0.003	.990
*Anomia*				
Year of data collection	9	−0.000 (0.017)	−0.034, 0.033	.997
Mean sample age	12	0.001 (0.004)	−0.007, 0.010	.729
% Males in sample	12	0.005 (0.003)	−0.001, 0.011	.095
*Anti‐democratic*				
Year of data collection	8	0.010 (0.003)	0.004, 0.016	.001
Mean sample age	7	−0.010 (0.002)	−0.013, −0.007	.000
% Males in sample	7	−0.018 (0.003)	−0.025, −0.012	.000
*Anxiety*				
Year of data collection	7	0.017 (0.009)	−0.002, 0.035	.076
Mean sample age	7	−0.000 (0.001)	−0.003, 0.002	.809
*Authoritarian*				
Year of data collection	19	0.017 (0.013)	−0.009, 0.044	.201
Mean sample age	16	−0.002 (0.005)	−0.012, 0.008	.687
% Males in sample	18	−0.007 (0.007)	−0.020, 0.026	.294
*Coll. Rel. Dep*.				
Year of data collection	18	0.004 (0.005)	−0.007 (0.014)	.483
Mean sample age	18	−0.001 (0.004)	−0.009, 0.008	.884
% Males in sample	19	−0.002 (0.001)	−0.004, −0.000	.040
*Depression*				
Year of data collection	10	0.018 (0.012)	0.004, 0.052	.022
Mean sample age	7	−0.004 (0.007)	−0.017, 0.009	.549
% Males in sample	10	0.000 (0.002)	−0.004, 0.004	.938
*Deviant Peers*				
Year of data collection	12	−0.015 (0.008)	−0.031, 0.001	.074
Mean sample age	10	0.004 (0.007)	−0.010, 0.017	.584
% Males in sample	11	−0.000 (0.003)	−0.005, 0.005	.877
*Disconnected*				
Year of data collection	7	0.024 (0.014)	−0.002, 0.051	.073
Mean sample age	7	0.007 (0.023)	−0.038, 0.052	.764
% Males in sample	7	−0.001 (0.003)	−0.008, 0.006	.736
*Education*				
Year of data collection	30	−0.001 (0.004)	−0.009, 0.007	.864
Mean sample age	21	0.005 (0.002)	0.001, 0.009	.018
% Males in sample	28	0.000 (0.002)	−0.004, 0.004	.923
*Exp. Discrimination*				
Year of data collection	21	−0.006 (0.002)	−0.010, −0.002	.004
Mean sample age	18	−0.000 (0.001)	−0.003, 0.002	.752
% Males in sample	21	0.000 (0.001)	−0.002, 0.003	.675
*Experienced violence*				
Year of data collection	16	−0.004 (0.003)	−0.011, 0.003	.227
Mean sample age	13	−0.004 (0.002)	−0.008, 0.000	.060
% Males in sample	15	0.001 (0.001)	−0.001, 0.002	.303
*Gender*				
Year of data collection	46	0.007 (0.002)	0.002, 0.012	.003
Mean sample age	46	−0.003 (0.001)	−0.004, −0.001	.001
% Males in sample	54	−0.000 (0.001)	−0.003, 0.002	.709
*Identity fusion*				
Year of data collection	7	0.011 (0.014)	−0.017, 0.039	.441
% Males in sample	7	0.005 (0.002)	0.000, 0.009	.040
*Immigrant*				
Year of data collection	22	−0.004 (0.004)	−0.012, 0.004	.354
Mean sample age	18	−0.001 (0.001)	−0.003, 0.002	.539
% Males in sample	22	0.001 (0.001)	−0.001, 0.003	.183
*Indiv. Rel. Deprivation*				
Year of data collection	15	−0.003 (0.008)	−0.018, 0.012	.719
Mean sample age	10	−0.002 (0.003)	−0.007, 0.003	.391
% Males in sample	15	−0.003 (0.002)	−0.008, 0.002	.199
*In‐Group identity*				
Year of data collection	24	−0.007 (0.005)	−0.017, 0.003	.193
Mean sample age	20	−0.002 (0.003)	−0.008, 0.005	.609
% Males in sample	23	0.002 (0.002)	−0.001, 0.005	.147
*Institutional trust*				
Year of data collection	11	−0.023 (0.013)	−0.047, 0.002	.074
Mean sample age	10	0.002 (0.004)	−0.006, 0.011	.590
% Males in sample	10	−0.002 (0.004)	−0.009, 0.005	.613
*Juvenile delinquency*				
Mean sample age	7	0.062 (0.041)	−0.018, 0.143	.130
% Males in sample	7	−0.045 (0.016)	−0.077, −0.013	.006
*Legitimacy*				
Year of data collection	10	0.018 (0.012)	−0.005, 0.042	.128
Mean sample age	8	−0.001 (0.005)	−0.011, 0.009	.840
% Males in sample	9	0.009 (0.006)	−0.002, 0.021	.118
*Life events*				
Year of data collection	8	0.010 (0.006)	−0.001, 0.002	.079
% Males in sample	8	0.004 (0.008)	−0.013, 0.021	.639
*Low integration*				
Year of data collection	21	0.002 (0.007)	−0.011, 0.016	.742
Mean sample age	19	−0.000 (0.003)	−0.007, 0.006	.962
% Males in sample	20	−0.000 (0.002)	−0.003, 0.003	.982
*Marital status*				
Year of data collection	11	−0.004 (0.003)	−0.010, 0.001	.108
Mean sample age	10	0.001 (0.001)	−0.001, 0.004	.379
% Males in sample	11	−0.002 (0.002)	−0.005, 0.002	.352
*Moral neutralizations*				
Year of data collection	15	0.035 (0.013)	0.009, 0.061	.009
Mean sample age	14	0.001 (0.006)	−0.011, 0.012	.904
% Males in sample	15	−0.007 (0.004)	−0.015, 0.002	.129
*Parental involvement*				
Year of data collection	12	0.020 (0.013)	−0.005, 0.044	.122
% Males in sample	12	−0.006 (0.002)	−0.010, −0.002	.006
*Perceived discriminate*				
Year of data collection	8	0.000 (0.008)	−0.015, 0.016	.978
Mean sample age	6	0.000 (0.004)	−0.007, 0.008	.908
% Males in sample	8	0.000 (0.005)	−0.009, 0.010	.938
*Perceived injustice*				
Year of data collection	8	−0.003 (0.009)	−0.021, 0.015	.742
Mean sample age	6	0.009 (0.009)	−0.009, 0.028	.318
% Males in sample	8	0.001 (0.002)	−0.003, 0.005	.478
*Police contact*				
Year of data collection	6	0.035 (0.016)	0.003, 0.066	.031
*Political efficacy*				
Year of data collection	7	−0.015 (0.011)	−0.036, 0.006	.164
Mean sample age	7	0.006 (0.003)	−0.000, 0.012	.072
% Males in sample	7	−0.006 (0.008)	−0.021, 0.009	.441
*Political extremism*				
Year of data collection	6	0.051 (0.024)	0.004, 0.097	.032
Mean sample age	6	−0.032 (0.006)	−0.045, −0.020	.000
% Males in sample	6	−0.021 (0.004)	−0.030, −0.013	.000
*Political grievances*				
Year of data collection	8	0.008 (0.010)	−0.012, 0.029	.436
Mean sample age	7	0.000 (0.003)	−0.006, 0.007	.948
% Males in sample	8	−0.003 (0.002)	−0.007, 0.001	.201
*Political satisfaction*				
Year of data collection	6	−0.023 (0.005)	−0.033, −0.013	.000
% Males in sample	6	−0.014 (0.015)	−0.044, 0.015	.337
*Religiosity*				
Year of data collection	19	−0.000 (0.006)	−0.013, 0.012	.990
Mean sample age	14	0.006 (0.004)	−0.002, 0.015	.118
% Males in sample	19	0.004 (0.003)	−0.002, 0.010	.215
*School bonding*				
Year of data collection	7	−0.014 (0.004)	−0.022, −0.006	.000
% Males in sample	7	−0.000 (0.003)	−0.006, 0.005	.880
*School performance*				
Year of data collection	8	0.009 (0.010)	−0.010, 0.028	.346
% Males in sample	7	−0.015 (0.018	−0.050, 0.020	.400
*Self‐control*				
Year of data collection	8	0.013 (0.008)	0.002, 0.029	.096
Mean sample age	7	0.024 (0.015)	−0.004, 0.053	.097
% Males in sample	8	−0.000 (0.003)	−0.005, 0.005	.997
*Self‐sacrifice*				
Mean sample age	6	0.006 (0.013)	−0.019, 0.031	.630
% Males in sample	6	−0.001 (0.004)	−0.010, 0.007	.745
*SES*				
Year of data collection	25	−0.006 (0.003)	−0.011, −0.001	.024
Mean sample age	21	0.000 (0.001)	−0.002, 0.002	.803
% Males in sample	25	0.000 (0.001)	−0.002, 0.003	.781
*Significance quest*				
Mean sample age	10	−0.000 (0.005)	−0.010, 0.009	.932
% Males in sample	10	0.003 (0.003)	−0.002, 0.008	.215
*Social support*				
Year of data collection	7	0.001 (0.006)	−0.011, 0.013	.929
% Males in sample	7	0.009 (0.007)	−0.005, 0.024	.186
*Superiority*				
Year of data collection	13	−0.010 (0.014)	−0.037, 0.017	.476
Mean sample age	9	−0.006 (0.012)	−0.030, 0.017	.609
% Males in sample	13	0.010 (0.005)	0.000, 0.020	.047
*Thrill‐seeking*				
Year of data collection	17	0.014 (0.011)	−0.008, 0.036	.201
Mean sample age	12	0.005 (0.004)	−0.003, 0.012	.194
% Males in sample	17	0.010 (0.004)	.003, .017	.007
*Uncertainty*				
Year of data collection	10	0.001 (0.005)	−0.009, 0.010	.909
Mean sample age	9	−0.002 (0.003)	−.008, .004	.520
% Males in sample	10	0.002 (0.002)	−0.002, 0.006	.269
*Unemployed*				
Year of data collection	13	−0.002 (0.003)	−0.007, 0.004	.550
Mean sample age	7	−0.001 (0.001)	−0.004, 0.001	.266
% Males in sample	9	0.005 (0.002)	0.002, 0.009	.004
*Violent media*				
Year of data collection	6	0.007 (0.004)	−0.002, 0.016	.116
*Welfare*				
Year of data collection	9	−0.000 (0.004)	−.007, .007	.934
% Males in sample	9	0.005 (0.005)	−0.004, 0.014	.276
*Worship attend*.				
Year of data collection	13	0.012 (0.006)	−0.000, 0.023	.052
Mean sample age	9	−0.001 (0.003)	−0.008, .005	.683
% Males in sample	11	−0.001 (0.002)	−.004, .002	.547
**Radical intentions**				
Activist intentions				
Year of data collection	18	0.017 (0.019)	−0.021, 0.055	.375
Mean sample age	17	−0.009 (0.008)	−0.024, 0.007	.271
% Males in sample	18	−0.004 (0.003)	−0.009, 0.002	.200
Age				
Year of data collection	25	−0.001 (0.007)	−0.014, 0.012	.848
Mean sample age	16	−0.004 (0.004)	−0.012, 0.005	.394
% Males in sample	23	−0.002 (0.001)	−0.005, 0.000	.057
Anger				
Year of data collection	12	−0.003 (0.055)	−0.112, 0.105	.950
Mean sample age	6	−0.023 (0.013)	−0.048, 0.002	.070
% Males in sample	12	0.004 (0.008)	−0.012, 0.020	.631
Anomie				
Mean sample age	6	0.024 (0.004)	0.017, 0.030	.000
% Males in sample	6	0.012 (0.007)	−0.002, 0.025	.085
Coll. Relative Dep.				
Year of data collection	17	0.033 (0.013)	0.007, 0.059	.013
Mean sample age	11	0.007 (0.013)	−0.018, 0.032	.598
% Males in sample	17	−0.004 (0.004)	−0.012, 0.004	.363
Commitment				
Year of data collection	9	0.035 (0.028)	−0.019, 0.089	.209
Mean sample age	8	−0.008 (0.014)	−0.036, 0.019	.562
% Males in sample	9	−0.005 (0.004)	−0.013, 0.003	.198
Education				
Year of data collection	12	0.001 (0.010)	−0.018, 0.020	.932
% Males in sample	12	0.000 (0.002)	−0.003, 0.003	.933
Gender				
Year of data collection	28	0.008 (0.006)	−0.005, 0.020	.218
Mean sample age	18	−0.006 (0.004)	−0.013, 0.001	.096
% Males in sample	25	−0.000 (0.002)	−0.004, 0.003	.812
Immigrant				
% Males in sample	8	−0.002 (0.012)	−0.025, 0.021	.852
In‐Group Connected				
Year of data collection	6	−0.011 (0.013)	−0.036, 0.014	.387
Mean sample age	6	−0.004 (0.006)	−0.015, 0.007	.490
% Males in sample	6	0.001 (0.004)	−0.006, 0.008	.783
In‐Group Identity				
Year of data collection	15	0.029 (0.009)	0.011, 0.047	.002
Mean sample age	7	0.004 (0.007)	−0.010, 0.018	.571
% Males in sample	14	−0.003 (0.003)	−0.009, 0.003	.323
Low integration				
Year of data collection	9	0.016 (0.008)	−0.000, 0.032	.050
Mean sample age	9	0.006 (0.005)	−.004, .015	.226
% Males in sample	9	−0.004 (0.003)	−0.010, 0.002	.242
Moral neutralizations				
Year of data collection	8	0.145 (0.171)	−0.191, 0.480	.398
Mean sample age	8	0.005 (0.010)	−0.015, 0.026	.605
% Males in sample	8	0.017 (0.012)	−0.006, 0.040	.138
Openness				
% Males in sample	7	0.003 (0.010)	−0.018, 0.023	.806
Perceived injustice				
Year of data collection	13	0.024 (0.019)	−0.014, 0.062	.213
% Males in sample	13	0.000 (0.017)	−0.013, 0.014	.953
Radical attitudes				
Year of data collection	11	0.000 (0.012)	−0.022, 0.023	.980
Mean sample age	11	−0.013 (0.006)	−0.025, −0.001	.039
% Males in sample	11	−0.003 (0.002)	−0.008, 0.001	.122
Realistic threat				
Year of data collection	6	0.005 (0.019)	−0.032, 0.042	.793
% Males in sample	6	−0.013 (0.012)	−0.036, 0.011	.288
Self‐esteem				
Year of data collection	6	0.022 (0.036)	−0.049, 0.093	.548
Mean sample age	6	−0.066 (0.032)	−0.129, −0.003	.040
% Males in sample	6	0.005 (0.003)	−0.000, 0.011	.072
SES				
Mean sample age	7	−0.000 (0.004)	−0.008, 0.008	.911
% Males in sample	11	0.001 (0.002)	−0.003, 0.004	.686
Symbolic threat				
Year of data collection	6	0.031 (0.016)	−0.001, 0.062	.054
% Males in sample	6	−0.028 (0.007)	−0.041, −0.015	.000
**Radical behaviors**				
*Gender*				
Year of data collection	6	−0.060 (0.026)	−0.111, −0.009	.022
*Employment*				
Year of data collection	6	0.014 (0.008)	−0.002, 0.031	.093
Mean sample age	6	−0.023 (0.027)	−0.076, 0.030	.391
% Males in sample	7	−0.002 (0.003)	−0.008, 0.003	.359
*Age*				
Year of data collection	7	−0.003 (0.012)	−0.026, 0.020	.787
Mean sample age	6	−0.008 (0.012)	−0.032, 0.015	.486
% Males in sample	7	−0.001 (0.004)	−0.008, 0.007	.886

*Note: k*  = number of effect sizes; *B* = regression coefficient with *SE* in parentheses and 95% confidence intervals, based on a univariate meta‐regression of the linear relationship between the study‐level characteristic and *r* correlation. *p* = *p* value for z test.

Moderator analysis assessed the effects of three key categorical variables, namely region in which a study was carried out (EU, US and Other), type of ideology examined (right‐wing, left‐wing, Islamist, nonspecific/mixed, and Other), and effect size derivation (Bivariate or standardized partial effect size).

e adopted a minimalist approach in which at least two effect sizes from at least two categories were needed in order to perform an analysis for any of these three variables. Tables 9, 10, 11 detail the results from the moderator analyses and demonstrate that significant between‐group heterogeneity exists for both categorical variables across a number of factors.

**Table 9:** Moderator analysis for region

Factor	Region	k	*r*	95% CI	*Q*_Between_	p
**Radical attitudes**						
2nd gen. immigrant	EU	8	.01	−0.03, 0.05	.000	.987
	Canada	3	.01	−0.02, 0.04		
Age	EU	38	−.04*	−0.08, −0.01	.439	.803
	US	5	−.08	−0.19, 0.03		
	Other (mixed)	3	−.04	−0.19, 0.12		
Anomia	EU	8	.20***	0.14, 0.26	.616	.433
	US	4	.15**	0.05, 0.25		
Anxiety	EU	4	.04	−0.02, 0.10	.000	.983
	Other (mixed)	4	.04	−0.03, 0.10		
Authoritarianism	EU	15	.27***	0.17, 0.36	2.577	.276
	US	4	.12	−0.10, 0.33		
	Other (Australia)	2	.40**	0.11, 0.63		
Depression	EU	5	−.01	−0.12, 0.11	.067	.967
	US	2	.01	−0.18, 0.20		
	Canada	3	.02	−.13, .16		
Education	EU	24	−.06**	−0.10, −0.02	.521	.771
	US	4	−.07	−0.18, 0.03		
	Other (mixed)	2	−.12	−0.26, 0.03		
Exp. discrimination	EU	15	.07***	0.05, 0.09	4.657	.097
	US	3	.11**	0.05, 0.17		
	Other (mixed)	2	.02	−.05, .10		
Exp. violence	EU	9	.09***	0.06, 0.11	10.961	.004
	US	4	.07*	0.01, 0.14		
	Other (Canada)	3	.02	−0.03, 0.08		
Gender (male)	EU	41	.11***	0.09, 0.13	7.360	.025
	US	4	.02	−.04, .08		
	Other (mixed)	11	.09***	0.05, 0.14		
Immigrant	EU	17	.01	−0.02, 0.05	.306	.858
	US	4	−.01	−.07, .06		
	Other (Australia)	2	−.01	−0.12, 0.10		
In‐group identity	EU	18	.08***	0.03, 0.12	.027	.870
	Other (Mixed)	7	.07†	−0.00, 0.14		
In‐group superiority	EU	11	.35***	0.25, 0.44	.281	.596
	Other (Mixed)	2	.28*	0.04, 0.50		
Institutional trust	EU	9	−.12*	−0.21, −0.02	8.435	.002
	Other (Mixed)	2	−.44***	−0.60, −0.25		
Low integration	EU	16	.16***	0.11, 0.21	13.206	.001
	US	3	.22***	0.11, 0.33		
	Other (Mixed)	2	.42***	0.29, 0.53		
Marital status	EU	10	−.05***	−0.07, 0.02	.573	.449
	Other (Mixed)	2	−.02	−0.08, 0.03		
Moral neutralizations	EU	11	.26***	0.16, 0.35	6.313	.045
	US	2	.47***	0.26, 0.63		
	Other (Mixed)	2	.48***	0.27, 0.64		
Parental involvement	EU	9	−.12***	−0.17, −0.07	2.957	.086
	Other (Canada)	3	−.04	−0.12, 0.05		
Perceived discriminate	EU	6	.16***	.11, .21	.929	.335
	US	2	.11*	0.01, 0.20		
Political extremism	EU	4	.38**	0.17, 0.57	.020	.888
	US	2	.36*	0.05, 0.61		
Political grievances	EU	6	.14***	0.06, 0.22	.039	.843
	US	2	.16*	0.03, 0.28		
Religiosity	EU	10	−.01	−0.09, 0.08	3.494	.174
	US	4	.14*	−0.00, 0.28		
	Other (Mixed)	5	.09	−0.05, 0.22		
Self‐sacrifice	EU	3	.17***	0.11, 0.23	.550	.458
	US	2	.12*	0.02, 0.23		
Socioeconomic status	EU	23	−.05***	−0.07, −0.02	26.403	.000
	US	2	−.13**	−0.22, −0.04		
	Other (Mixed)	2	.14***	0.07, 0.21		
Significance quest	EU	4	.10*	0.01, 0.19	1.649	.438
	US	2	.17*	0.03, 0.30		
	Other (Mixed)	4	.18***	0.09, 0.27		
Worship attendance	EU	8	.07*	0.00, 0.13	.087	.767
	US	4	.09†	−0.01, 0.18		
**Radical Intentions**						
Activism intentions	EU	6	.55***	0.41, 0.66	4.177	.124
	US	10	.29†	−0.02, 0.54		
	Other (Canada)	2	.40***	.28, .51		
Age	EU	15	−.07*	−0.13, −0.00	.144	.930
	US	7	−.08†	−0.17, 0.01		
	Other (Mixed)	3	−.10	−0.24, 0.05		
Anger	EU	7	.42***	0.27, 0.56	.616	.735
	US	3	.31*	0.05, 0.54		
	Other (Mixed)	2	.42*	0.10, 0.66		
Commitment	EU	3	.54***	0.37, 0.68	6.117	.047
	US	4	.41***	0.25, 0.54		
	Other (Canada)	2	.30***	.19, .39		
Education	EU	7	−.04	−0.14, 0.05	.321	.571
	US	5	−.01	−.08, .06		
Gender (male)	EU	16	.09**	0.02, 0.16	.530	.767
	US	9	.12***	.07, .18		
	Other (Mixed)	3	.11	−0.03, 0.24		
In‐group identity	EU	13	.27***	0.14, 0.38	2.681	.102
	Other (Mixed)	2	.16***	0.11, 0.20		
Moral neutralization	EU	4	.29†	−0.04, 0.57	.662	.416
	US	4	.42***	0.33, 0.51		
Openness	EU	5	−.22**	−0.36, −0.07	1.172	.279
	US	5	−.12**	−0.21, −0.03		
Power distance orientation	US	3	.17***	0.10, 0.23	1.178	.278
	Other (Mixed)	2	.30*	.06, .51		
Radical attitudes	EU	8	.49***	0.37, 0.60	.180	.671
	Other (Mixed)	4	.45***	0.29, 0.59		
Self‐esteem	EU	4	.32**	0.11, 0.49	8.015	.005
	Other (Mixed)	2	.01	−.04, .06		
SocioECONOMIC Status	EU	9	−.04	−0.11, 0.03	.541	.462
	US	2	.00	−0.08, 0.08		
Uncertainty	EU	2	.05	−.05, .14	.013	.908
	US	3	.05**	.01, .09		
**Radical behaviors**						
Age	EU	2	−.19***	−0.27, −0.10	3.869	.144
	US	3	−.16	−0.38, 0.08		
	Other (Mixed)	3	.02	−0.17, 0.20		
Marital status	US	2	−.04	−0.11,0.03	.095	.758
	Other (Mixed)	3	−.03	−0.08, 0.03		
Unemployment	EU	2	.29***	0.27, 0.31	15.737	.000
	US	2	.06	−.06, .18		
	Other (Mixed)	3	.19	−0.07, 0.42		

*Note*: k = number of effect sizes; *Q*_Between_ = between‐group heterogeneity test of the difference among/between the pooled effect sizes, with associated significance value (p). All estimates are *r* correlations.

*** <.001.

** <.01.

* <.05.

^†^ <.10.

**Table 10:** Moderator analysis for ideological strain

Factor	Ideology	k	*r*	95% CI	*Q*_Between_	p
**Radical attitudes**						
2nd gen. immigrant	Nonspecific	7	.02	−0.02, 0.06	.621	.431
	Islamist	4	−.02	−0.09, 0.06		
Age	Nonspecific	19	−.04†	−.09, .00	4.471	.215
	Islamist	20	−.07*	−0.11, −0.02		
	Left‐wing	3	−.07	−0.24, 0.11		
	Right‐wing	4	.04	−0.05, 0.12		
Anger	Nonspecific	2	.11***	0.07, 0.14	.836	.360
	Islamist	2	.21†	−0.02, 0.42		
Anomia	Nonspecific	2	.24***	0.22, 0.26	7.618	.022
	Islamist	7	.13†	−.00, .26		
	Right‐wing	2	.27***	0.25, 0.30		
Antidemocratic attitude	Nonspecific	3	.19***	0.13, 0.25	.263	.608
	Islamist	3	.16**	0.04, 0.27		
Anxiety	Nonspecific	6	.03	−.01, .07	.262	.609
	Islamist	2	.08	−0.09, 0.24		
Authoritarianism	Nonspecific	7	.19†	−0.00, 0.38	5.134	.162
	Islamist	9	.28**	0.08, 0.45		
	Left‐wing	2	.11	−0.18, 0.38		
	Right‐wing	3	.40***	0.26, 0.52		
Coll. relative deprivation	Nonspecific	5	.14**	0.06, 0.22	4.418	.110
	Islamist	10	.14***	0.07, 0.20		
	Right‐wing	3	.21***	0.17, 0.25		
Depression	Nonspecific	4	.04	−0.07, 0.16	1.161	.281
	Islamist	4	−.04	−.014, 0.06		
Deviant peers	Nonspecific	4	.21***	0.14, 0.28	2.818	.244
	Islamist	2	.13***	0.06, 0.19		
	Right‐wing	5	.16†	−0.03, 0.34		
Education	Nonspecific	6	−.07*	−0.13. −0.01	.245	.970
	Islamist	18	−.05***	−0.08, −0.03		
	Left‐wing	2	−.07	−0.21, −0.07		
	Right‐wing	4	−.07	−0.32, 0.18		
Exp. discrimination	Nonspecific	6	.07***	0.03, 0.10	.082	.775
	Islamist	12	.07**	0.03, 0.11		
Exp. violence	Nonspecific	6	.07***	0.04, 0.10	.883	.659
	Islamist	7	.10*	0.01, 0.18		
	Right‐wing	2	.08***	0.04, 0.12		
Gender (male)	Nonspecific	24	.09***	0.07, 0.12	25.183	.000
	Islamist	20	.06***	0.04, 0.09		
	Left‐wing	8	.16***	0.12, 0.20		
	Right‐wing	4	.17***	0.13, 0.21		
Identity fusion	Nonspecific	3	.16†	−0.01, 0.33	.085	.771
	Islamist	2	.19***	0.11, 0.27		
Immigrant	Nonspecific	11	−.01	−0.05, 0.03	.736	.382
	Islamist	11	.02	−0.03, 0.07		
Indv. Rel. deprivation	Nonspecific	4	.11**	0.04, 0.17	1.866	.601
	Islamist	5	.07**	0.02, 0.12		
	Left‐wing	2	.14**	0.05, 0.22		
	Right‐wing	4	.13†	−0.01, 0.27		
In−group identity	Nonspecific	10	.01	−0.02, 0.05	5.300	.021
	Islamist	13	.08**	0.03, 0.13		
In−group superiority	Nonspecific	3	.25†	−0.01, 0.48	.911	.634
	Islamist	6	.37***	0.22, 0.50		
	Right‐wing	3	.39***	0.20, 0.56		
Institutional trust	Nonspecific	4	−.03***	−0.04, 0−.01	4.424	.035
	Islamist	5	−.26*	−0.45, −0.04		
Law legitimacy	Nonspecific	6	.16***	0.10, 0.22	5.255	.072
	Islamist	2	.49**	0.22, 0.70		
	Right‐wing	2	.16***	0.13, 0.20		
Life events	Nonspecific	3	.04	−0.04, 0.12	1.494	.222
	Islamist	4	−.02	−0.08, 0.04		
Low integration	Nonspecific	7	.20***	0.11, 0.29	.017	.991
	Islamist	12	.20***	0.16, 0.25		
	Right‐wing	2	.19*	0.03, 0.34		
Marital status	Nonspecific	3	−.04*	−0.08, 0.00	.054	.817
	Islamist	9	−.05**	−0.08, −0.02		
Moral neutralizations	Nonspecific	8	.38***	0.23, 0.51	2.733	.255
	Islamist	4	.25***	0.19, 0.30		
	Right‐wing	2	.24**	0.10, 0.38		
Parental abuse	Islamist	2	.17***	0.10, 0.23	13.020	.001
	Left‐wing	2	.16***	0.13, 0.19		
	Right‐wing	2	.10***	0.07, 0.12		
Parental involvement	Nonspecific	6	−.07*	−0.13, −0.01	3.960	.266
	Islamist	2	−.13†	0.28, 0.02		
	Left‐wing	2	−.17***	−0.25, −0.09		
	Right‐wing	2	−.09	−0.19, 0.02		
Perceived discrimination	Nonspecific	2	.23***	0.21, 0.25	32.277	.000
	Islamist	4	.11***	0.07, 0.15		
Perceived injustice	Islamist	4	.10*	0.01, 0.19	.007	.932
	Right‐wing	3	.10***	0.06, 0.14		
Political efficacy	Nonspecific	2	−.00	−0.04, 0.03	.091	.763
	Islamist	3	−.02	−0.13,0.09		
Political grievances	Nonspecific	3	.17***	0.13, 0.20	.247	.619
	Islamist	5	.14**	0.03, 0.25		
Political participation	Nonspecific	2	.04	−0.07, 0.15	.661	.416
	Islamist	2	−.01	−0.05, 0.03		
Prayer frequency	Islamist	3	.06*	0.01, 0.11	27.503	.000
	Right‐wing	2	.30***	0.23, 0.37		
Religiosity	Nonspecific	7	−.08	0−.20, 0.06	6.262	.044
	Islamist	8	.10**	0.04, 0.17		
	Right‐wing	3	.15	−0.07, 0.36		
School bonding	Islamist	2	−.14***	−0.21, −0.07	.831	.660
	Left‐wing	2	−.14**	−0.22, −0.06		
	Right‐wing	2	−.11***	−0.14, −0.08		
School performance	Islamist	2	−.07	−0.17, 0.03	.409	.815
	Left‐wing	2	−.13†	−0.27, 0.04		
	Right‐wing	3	−.08	−0.18, 0.02		
Self−control	Nonspecific	3	.22***	0.12, 0.32	1.213	.545
	Islamist	2	.20*	0.03, 0.36		
	Right‐wing	2	.28***	0.20, 0.36		
Self−sacrifice	Nonspecific	3	.23*	0.05, 0.40	.492	.483
	Islamist	3	.16***	0.08, 0.24		
Socioeconomic status	Nonspecific	13	−.04*	−0.06, −0.01	.000	.990
	Islamist	13	−.04	−0.08, 0.01		
Significance quest	Nonspecific	5	.13*	0.01, 0.25	.676	.713
	Islamist	3	.18***	0.10, 0.26		
	Left‐wing	2	.14**	0.04, .23		
Social support	Nonspecific	3	−.11*	−0.18, −0.03	.403	.525
	Islamist	2	−.06	−0.17, 0.04		
Student	Nonspecific	2	.07*	0.01, 0.12	1.417	.234
	Islamist	3	−.05	−0.24, 0.14		
Symbolic threat	Nonspecific	2	.28***	.17, .39	.184	.912
	Islamist	2	.33**	0.01, 0.60		
	Right‐wing	2	.31***	0.26, 0.35		
Thrill−seeking	Nonspecific	6	.30***	0.19, 0.40	2.658	.265
	Left‐wing	6	.36***	0.25, 0.46		
	Right‐wing	4	.23***	0.10, 0.34		
Uncertainty	Nonspecific	4	.07**	0.03, 0.11	.386	.534
	Islamist	5	.10†	−0.01, 0.22		
Unemployment	Nonspecific	6	.04***	0.02, 0.06	1.978	.160
	Islamist	6	.07**	0.03, 0.12		
Violent media	Nonspecific	2	.11	−0.03, 0.25	.007	.933
	Right‐wing	2	.12**	0.04, 0.19		
Welfare recipient	Nonspecific	2	.03**	0.01, 0.06	15.112	.002
	Islamist	3	.04	−0.02, 0.09		
	Left‐wing	2	.09***	0.07, 0.11		
	Right‐wing	2	.04**	0.02, 0.06		
Worship attendance	Nonspecific	3	.08	−.06, .21	1.489	.475
	Islamist	8	.03	−0.02, 0.08		
	Right‐wing	2	.16	−0.06, 0.36		
**Radical intentions**						
Age	Nonspecific	14	−.08**	−0.14, −0.02	1.291	.524
	Islamist	9	−.06†	−0.12, 0.00		
	Left‐wing	2	−.13*	−0.23, −0.02		
Anger	Nonspecific	2	.11*	−0.01, 0.23	15.832	.000
	Islamist	6	.46***	0.33, 0.57		
	Left‐wing	3	.31***	0.16, 0.44		
Coll. Rel. deprivation	Nonspecific	3	.22***	0.13, 0.31	3.640	.056
	Islamist	12	.37***	0.25, 0.49		
Commitment to cause	Nonspecific	2	.62***	0.52, 0.70	13.721	.000
	Left‐wing	7	.37***	0.27, 0.46		
Education	Nonspecific	4	−.05†	−0.10, 0.00	.418	.518
	Islamist	7	−.01	−0.11, 0.09		
Gender	Nonspecific	14	.10***	0.05, 0.14	.501	.778
	Islamist	11	.11*	0.02, 0.21		
	Left‐wing	2	.17	−0.04, 0.35		
In−group connectedness	Nonspecific	2	.30***	0.19, 0.40	1.458	.227
	Islamist	4	.20**	0.09, 0.31		
In−group identity	Nonspecific	3	.14***	0.10, 0.18	3.075	.080
	Islamist	10	.30**	0.13, 0.46		
Moral neutralizations	Nonspecific	2	.04	−0.17, 0.24	13.699	.001
	Islamist	2	.52***	0.28, 0.70		
	Left‐wing	4	.42***	0.33, 0.51		
Openness	Nonspecific	7	−.10**	−0.18, −0.03	6.819	.009
	Islamist	3	−.33***	−0.47, −0.18		
Perceived injustice	Nonspecific	2	.07	−0.32, 0.44	1.764	.184
	Islamist	10	.35***	0.21, 0.47		
Radical attitudes	Nonspecific	6	.46***	0.28, 0.60	.179	.915
	Islamist	4	.49***	0.42, 0.55		
	Right‐wing	2	.48***	0.44, 0.52		
Self−esteem	Nonspecific	4	.20	−0.07, 0.44	.001	.971
	Islamist	2	.19*	0.01, 0.37		
Socioeconomic status	Nonspecific	4	−.06	−0.14, 0.03	1.392	.238
	Islamist	6	.01	−0.05, 0.06		
Symbolic threat	Nonspecific	2	.26***	0.18, 0.35	.179	.672
	Islamist	3	.35†	−0.04, 0.64		
**Radical behaviors**						
Age	Nonspecific	4	−.03	−0.24, 0.19	1.171	.279
	Islamist	4	−.18*	−0.34, −0.01		
Gender	Nonspecific	2	.24	−0.10, 0.53	5.181	.023
	Islamist	2	.67***	0.43, 0.83		
Marital status	Nonspecific	3	−.06**	−0.11, −0.02	7.000	.008
	Islamist	3	.00	−0.01, 0.01		
Unemployment	Nonspecific	3	.26***	0.15, 0.36	1.941	.164
	Islamist	4	.13†	−0.02, 0.27		

*Note: k*  = number of effect sizes; *Q*_Between_ = between−group heterogeneity test of the difference among/between the pooled effect sizes, with associated significance value (*p*). All estimates are *r* correlations.

*** <.001.

** <.01.

* <.05.

^†^ <.10.

**Table 11:** Moderator analysis for effect size derivation

Factor	ES type	k	*r*	95% CI	*Q*_Between_	p
**Radical attitudes**						
2nd gen. immigrant	Bivariate	5	−.00	−.07, .07	.048	.827
	Partial	6	.01	−.01, .02		
Age	Bivariate	24	−.04†	−.09, .00	.101	.750
	Partial	21	−.05**	−.09, −.01		
Antidemocratic attitude	Bivariate	5	.22***	.21, .24	1.864	.172
	Partial	3	.15**	.04, .26		
Anxiety	Bivariate	6	.05*	.01, .10	4.184	.041
	Partial	2	−.01	−.04, .02		
Authoritarianism	Bivariate	18	.24***	.12, .35	.929	.335
	Partial	3	.33***	.17, .48		
Coll. Rel. deprivation	Bivariate	16	.17***	.13, .20	1.589	.208
	Partial	3	.10†	.00, .20		
Depression	Bivariate	7	.01	−.08, .09	.010	.920
	Partial	3	−.01	−.19, .18		
Education	Bivariate	14	−.08*	−.16, −.01	1.047	.306
	Partial	16	−.04**	−.07, −.01		
Exp. discrimination	Bivariate	12	.09***	.07, .11	11.425	.001
	Partial	8	.02	−.01, .06		
Exp. violence	Bivariate	10	.07***	.04, .10	.118	.731
	Partial	6	.08***	.04, .12		
Gender	Bivariate	37	.11***	.08, .13	1.106	.293
	Partial	19	.09***	.05, .12		
Immigrant	Bivariate	14	.01	−.03, .04	.001	.975
	Partial	9	.01	−.05, .06		
In‐group identity	Bivariate	17	.08**	.02, .14	.100	.752
	Partial	8	.07**	.02, .12		
Institutional trust	Bivariate	7	−.23**	−.38, −.08	3.191	.074
	Partial	4	−.07†	−.15, .01		
Marital status	Bivariate	3	−.04***	−.05, −.03	.018	.894
	Partial	9	−.04**	−.07, .01		
Police contact	Bivariate	4	.32***	.23, .41	.147	.702
	Partial	2	.25	−.11, .55		
Political grievances	Bivariate	5	.18***	.10, .25	1.535	.215
	Partial	3	.10*	.02, .19		
Religiosity	Bivariate	13	.08*	.01, .14	1.488	.223
	Partial	6	−.02	−.15, .12		
School performance	Bivariate	6	−.10*	−.18, −.01	.556	.456
	Partial	2	−.06***	−.08, −.05		
Socioeconomic status	Bivariate	16	−.04**	−.07, −.02	.596	.440
	Partial	10	−.02	−.07, .03		
Uncertainty	Bivariate	4	.09*	−.00, .18	.343	.558
	Partial	6	.06*	−.00, .12		
Unemployment	Bivariate	5	.05**	.02, .08	.002	.966
	Partial	8	.05**	.02, .08		
Worship attendance	Bivariate	8	.06†	−.01, .03	.028	.868
	Partial	5	.07	−.02, .16		
**Radical behaviors**						
Thrill‐seeking	Bivariate	3	.19***	.14, .25	.002	.965
	Partial	3	.19***	.16, .22		
Unemployment	Bivariate	5	.16*	.01, .31	1.069	.301
	Partial	2	.26***	.14, .37		

*Note: k*  = number of effect sizes; *Q*_Between_ = between−group heterogeneity test of the difference among/between the pooled effect sizes, with the associated significance value (*p*). All estimates are *r* correlations.

*** <.001.

** <.01.

* <.05.

^†^ <.10.

In addition, we performed a one‐leave‐out analysis which assessed whether any single study was a significant contributor to heterogeneity. As the results in Table 12 demonstrate, the removal of a single study led to significant reductions in heterogeneity for a number of factors.

**Table 12:** Leave‐one‐out analysis

Factor	*r*	*95% CI*	*Q*	*I* ^*2*^	*N(k)*	^*Adjusted*^ *r*	95% CI	Q	*I* ^*2*^	N (k)
*Attitudes*										
Conscientiousness	−.12***	−0.15, −0.009	3.46	13.18	6156 (4)	−.13***	−.15, −.10	.55	0.00	5599 (3)
Extraversion	−.10	−0.29, 0.10	164.78***	98.18	6156 (4)	−.01	−.08, .06	6.54*	69.43	3732 (3)
General trust	−.06**	−0.11, −0.02	8.21*	63.44	32,546 (4)	−.09***	−.13, −.04	1.65	0.00	2356 (3)
Parental educ.	−.10***	−0.14, −0.05	28.24***	89.38	27,736 (4)	−.12***	−.14, .10	2.40	16.50	22,039 (3)
Parental control	−.12**	−0.20, −.04	65.28***	95.40	15647 (4)	−.09***	−.11, −.07	.64	0.00	9950 (3)
Political participation	.01	−.03, .06	6.87†	56.31	5258 (4)	−.01	−.04, .02	.01	0.00	4640 (3)
School bonding	−.13***	−.16, −.10	24.62***	75.63	22,174 (7)	−.11***	−.13, −.10	4.05	0.00	16,477 (6)
Self‐esteem (Indv.)	−.17**	−.29, −.05	8.43*	76.27	1801 (3)	−.11***	−.15, −.06	.30	0.00	1576 (2)
Self‐esteem (Public)	−.11	−.26, .04	13.27**	84.93	1801 (3)	−.18***	−.27, −.09	.53	0.00	448 (2)
Self‐esteem (Group)	−.11	−.27, .06	15.47***	87.07	1801 (3)	−.19***	−.28, −.10	.69	0.00	448 (2)
Social support	−.12***	−.15, −.08	19.07**	68.53	12,223 (7)	−.13***	−.16, −.10	9.63†	48.08	11,615 (6)
Teacher bonding	−.13***	−.18, −.08	7.62*	73.75	9105 (3)	−.15***	−.18, −.13	.044	0.00	6173 (2)
Anti‐democratic	.19***	.14, .23	43.05***	83.74	14,054 (8)	.22***	.20, .24	7.34	18.24	12,600 (7)
Alcohol	.04†	−.01, .09	22.13***	81.92	11,916 (5)	.02	−.02, .06	11.50**	73.92	11,298 (4)
Anger	.14***	.07, .20	6.69†	55.18	3475 (4)	.11***	.07, .14	.26	0.00	3364 (3)
Anxiety	.04†	−.00, .08	23.90*	70.71	10,409 (8)	.05*	.00, .09	17.95**	66.57	6730 (7)
APD	.03	−.07, .12	14.96*	79.95	4840 (4)	−.01	−.07, .05	5.52†	63.76	4794 (3)
Criminal history	.29***	.18, .40	26.75***	88.78	4976 (4)	.22***	.19, .25	1.72	0.00	4368 (3)
Disconnected	.16***	.08, .23	13.84*	56.65	2168 (7)	.14***	.07, .20	8.22	39.18	2002 (6)
Exp. Discrimination	.08***	.06, .10	74.80***	73.26	47,670 (21)	.07***	.05, 09	58.32***	67.42	46,760 (20)
Family violence	.10***	.06, .13	12.84*	68.86	15,923 (5)	.08***	.06, .10	.43	0.00	10,226 (4)
Dual identity	.15***	.09, .21	36.55***	83.59	10,140 (7)	.17***	.11, .22	10.95†	54.33	7208 (6)
Moved	.03	−.01, .08	16.94**	82.29	15735 (4)	.05*	.01, .09	7.93*	74.77	12,803 (3)
Neuroticism	−.02	−.10, .07	30.18***	90.059	6156 (4)	.02	−.04, .07	4.46	55.19	3732 (3)
Online posting	.11***	.05, .17	20.72***	85.52	12,715 (4)	.08**	.03, .14	3.51	42.95	8335 (3)
Parental abuse	.13***	.10, .17	21.13**	76.33	17711 (6)	.12***	.09, .15	8.39†	52.30	12,014 (5)
Perceived injustice	.08*	.01, .14	46.73***	85.02	7279 (8)	.10***	.06, .14	9.25	35.16	5613 (7)
Realistic threat	.35***	.26, .44	7.41†	59.51	1561 (4)	.31***	.22, .39	.71	0.00	475 (3)
Segregation	.15†	−.03, .32	27.926***	92.84	2437 (3)	.22***	.16, .29	.62	0.00	767 (2)
Self‐sacrifice	.20***	.09, .30	24.587***	79.66	1704 (6)	.16***	.10, .21	3.12	0.00	1234 (5)
Significance quest	.14***	.08, .21	19.598*	54.08	2165 (10)	.17***	.12, .22	7.43	5.82	1630 (8)
Similar peers	.31***	.18, .43	17.87***	88.81	7261 (3)	.22***	.19, .24	.40	0.00	7147 (2)
Student	−.00	−.11, .11	42.738***	90.641	3484(5)	.06**	.02, .10	1.30	0.00	3044 (4)
Symbolic threat	.31***	.24, .37	10.75†	53.47	2341 (6)	.30***	.26, .35	3.76	0.00	2180 (5)
Uncertainty	.07**	.02, .11	37.76***	76.17	20,960 (10)	.08***	.04, .12	26.84**	70.20	19,711 (9)
Unemployed	.05***	.03, .07	26.27*	54.31	52,596 (13)	.05***	.03, .07	20.85*	47.23	44,013 (12)
Welfare	.05***	.03, .07	17.86*	55.20	26,304 (9)	.04***	.03, .06	7.93	11.73	20,607 (8)
2nd gen immigrant	.01	−.02, .04	142.02***	92.96	85,719 (11)	−.00	−.01, .01	11.41	21.14	71,228 (10)
*Intentions*										
Conscientious	−.13***	−.16, −.10	9.87	39.21	7668 (7)	−.12***	−.14, −.10	3.01	0.00	6712 (6)
Outgroup friends	−.10	−.15, −.05	1.63	0.00	1398 (3)	−.12***	−.17, −.06	.24	0.00	1166 (2)
SDO	.09	−.16, .33	118.407***	96.622	1909 (5)	−.03	−.08, .03	1.12	0.00	1308 (4)
SES	−.03	−.09, .02	21.46*	53.40	3147 (11)	−.01	−.05, .03	8.02	0.00	2371 (10)
Anomia	.25***	.13, .37	48.286***	89.645	2425 (6)	.20***	.15, .26	2.72	0.00	1649 (5)
Harmonious passion	.16*	.03, .29	16.128**	75.199	922 (5)	.12***	−.01, .25	9.91*	69.73	750 (4)
Identity fusion	.52***	.45, .57	2.254	11.265	650 (3)	.48***	.41, .56	.16	0.00	416 (2)
In‐group connect.	.23***	.14, .32	12.542*	60.134	1118 (6)	.26***	.19, .33	5.12	21.90	865 (5)
In‐group superiority	.37***	.30, .45	9.089	55.989	1748 (5)	.40***	.34, .47	4.36	31.25	1516 (4)
Negative affect	.47***	.37, .56	8.890*	66.253	786 (4)	.43***	.33, .52	3.65	45.17	577 (3)
Neuroticism	.07***	.03, .10	15.996*	56.240	8308 (8)	.08***	.04, .11	10.37	42.15	7352 (7)
Positive affect	.16**	.06, .25	5.755	47.868	786 (4)	.11**	.03, .19	.211	0.00	577 (3)
Political efficacy (ext.)	.09	−.04, .22	19.989***	84.992	1630 (4)	.15*	.03, .26	6.01*	66.72	1017 (3)
PDO	.23**	.10, .35	24.968***	83.980	1645 (5)	.17***	.11, .23	.24	0.00	1044 (4)
Indv. Relative dep.	.14†	−.00, .28	14.082**	78.697	1558 (4)	.07	−.02, .16	.08	0.00	472 (3)
Self‐esteem (Indv.)	.20*	.00.38	41.312***	87.897	1789 (6)	.07	−.03, .17	6.51	38.57	1656 (5)
Significance quest	.11***	.06, .17	3.704	19.000	1603 (4)	.12**	.04, .21	2.87	30.37	827 (3)
*Behaviors*										
Marital	−.03	−.07, .01	25.270***	80.214	48,138 (6)	−.02	−.05, −.02	8.35†	52.11	45,905 (5)
Parental involvement	−.06***	−.08, −.02	6.226	51.818	13,069 (4)	−.04***	−.06, −.02	.06	0.000	8618 (3)
Law abidance	−.22***	−.26, −.18	5.924	66.242	8618 (3)	−.24***	−.27, −.21	0.00	0.00	3701 (2)
Anger	.20	−.06, .43	179.627***	98.887	5460 (3)	.08*	.02, .14	2.75†	63.66	3227 (2)
Deviant peers	.30**	.13, .46	228.430***	98.687	9627 (4)	.18***	.15, .21	3.60	44.46	8796 (3)
Exposure to violence	.11***	.07, .15	16.951**	76.402	11,435 (5)	.13***	.10, .16	5.71	47.44	9202 (4)

##### Year of data collection

For radical attitudes, the meta‐regressions found that year of data collection had a statistically significant negative impact on the estimates for Political Satisfaction, School Bonding, School Performance, Experiencing Discrimination and Police Contact, and a positive impact on the estimates for Perceived Injustice, Anti‐Democratic Attitudes, Moral Neutralizations, Low Self‐control, and Anxiety. A marginally significant (p < .10) negative impact was found for the estimate for Parental Involvement, and a positive impact on Political Efficacy, Societal Disconnectedness and Political Extremism. For radical intentions, year of data collection had a statistically significant positive impact on the pooled estimates for Radical Attitudes and Self‐Esteem, and a marginally significant impact on the estimates for Low Integration and In‐Group Identity. For the outcome of radical behaviors, there was a statistically significant negative impact on the estimate for Gender. Given that the included studies span a nearly two‐decade period, although are mostly from the 2018‐2020 period, the results indicate relative stability in the relationships between the factors and radicalization outcomes across time. However, temporality may be important for some factors, which may become more or less important over time (Table 8).

##### Mean age of sample

With regard to the average age of a sample, the meta‐regressions found that for the outcome of radical attitudes there was a statistically significant negative effect on the estimate for Age, and positive effects on the estimates for Political Efficacy, Education, Anti‐Democratic Attitudes and Political Extremism. A marginally significant positive effect was found for Low Self‐control. For radical intentions, the average age of a sample had a statistically significant positive impact on the pooled estimates for Anomie and Radical Attitudes and a negative impact for Self‐Esteem. For radical behaviors, a marginally significant, positive impact was found for the factor Welfare Recipient. The findings indicate that for the most part, the relationships between the analysed factors and radicalization outcomes are stable across age demographics, at least the level of studies' samples. However, for some factors, age can and does impact the magnitude of the relationship, a finding that needs to be taken into consideration when assessing the relevance of these factors in applied contexts (Table 8).

##### Proportion of males in a sample

With regard to the proportion of males in a sample, for radical attitudes the meta‐regressions found statistically significant negative effects on the estimates for Parental Involvement, Juvenile Delinquency, Collective Relative Deprivation, Political Extremism, Individual Relative Depravation, and Anti‐Democratic Attitudes. Statistically significant positive effects were found for Religiosity, Thrill‐Seeking/Risk Taking, Identity Fusion, Uncertainty and Unemployment. Marginally significant positive effects were found for Perceived Injustice. For the outcome of radical intentions, the proportion of males in studies' samples was found to have a statistically significant negative impact on the estimate for Symbolic threat, and a marginally significant impact on the estimates for Age and Anomie, which had a positive impact, and a negative impact on Self‐esteem. For the outcome of radical behaviors, the proportion of males in studies' samples had a statistically significant negative impact on the estimate for Radical Attitudes. The findings show that the gender‐composition of a sample can impact the estimated magnitude of the effects between a number of factors and radicalization outcomes. This needs to be taken into consideration when applying weights to risk factors, especially in the context of risk assessment, and when designing counter‐radicalization initiatives that seek to target these same factors (Table 7).

##### Region

Moderator analyses were carried out for the region in which a study was conducted for all factors which had at least two effect sizes from two or more different regions. Regions were categorized as European (EU), American (US) and other. The analysis was possible for 14 of the factors pertaining to the outcome of radical intentions, and 3 of the factors pertaining to the outcome of radical behaviors. The results of the analysis are displayed below in Table 9, with the factors arranged in alphabetical order. While between‐region heterogeneity was not statistically significant for the majority of the factors, it was for a small number of them. The findings indicate that for some factors, the regional context in which they are being assessed, such as in the context of risk assessment or counter‐radicalization interventions, needs to be considered.

For the outcome of radical attitudes, there were six factors with statistically significant between‐group heterogeneity and two factors which were marginally significant. With respect to Experiences of Discrimination, the estimate for EU studies (k = 15) was *r* = .07, for the US (k = 3) was *r* = .11 and for other democratic countries (k = 2) was a not statistically significant *r* = .02 (*Q*_Between_ = 4.657, p = .097). Similarly, for Experiences of Violence, the estimate for EU studies (k = 9) was *r* = .09, for the US (k = 4) was *r* = .07 and for other democratic countries (k = 2) was a not statistically significant *r* = .02 (*Q*_Between_ = 10.961, p = .004). For Gender, the estimate for EU studies (k = 41) was *r* = .11, for other democratic countries (k = 11) was *r* = .09, and for the US (k = 4) was a not statistically significant *r* = .02 (*Q*_Between_ = 4.657, p = .097). For both Institutional Trust and Low Integration, the estimates for other democratic countries were larger than for the EU. For Institutional Trust, the estimate for the EU (k = 9) was *r* = ‐.12 and for other democratic countries (k = 2) was *r* = ‐.44 (*Q*_Between_ = 8.435, p.002). For Low Integration, the estimate for the EU (k = 16) was *r* = .16, for the US (k = 3) *r* = .22 and for other democratic countries *r* = .42 (*Q*_Between_ = 13.206, p = .001). For Moral Neutralizations, the estimates for the US (k = 2) and other democratic countries (k = 2) were *r* = .47 and *r* = .48 respectively, larger than the estimate for the EU (k = 11) of *r* = .26 (*Q*_Between_ = 6.313, p = .045). While only marginally significant (*Q*_Between_ = 2.957, p = .086), with respect to Parental Involvement, the estimate for the EU (k = 9) of *r* = ‐.12 was larger than the estimate for other countries (which in this case were all Canadian studies, k = 3), which was a statistically nonsignificant *r* = ‐.04. Lastly, with respect to SocioEconomic Status, the estimate for the EU (k = 23) of *r* = ‐.05 was smaller than the estimate for the US (k = 2) of *r* = ‐.13, both of which were quite different from the positive estimate for other democratic countries (k = 2) of *r* = .14 (*Q*_Between_ = 26.403, p = .000).

With respect to the outcome of radical intentions, statistically significant between group heterogeneity was found for only two factors, namely Commitment to a Cause and Self‐Esteem. In both cases the estimates for the EU studies were larger than the US and other democratic countries. For Commitment to a Cause, the estimate for the EU (k = 3) was *r* = .54, for the US (k = 4) was *r* = .41 and for other democratic countries (k = 2) was *r* = .30 (*Q*_Between_ = 6.117, p = .047). For Self‐Esteem, the estimate for the EU (k = 4) was *r* = .32, whereas for other democratic countries (k = 2) was a nonsignificant *r* = .01 (*Q*_Between_ = 8.015, p = .005).

With respect to the outcome of radical behaviors, statistically significant between group heterogeneity was found for only for Unemployment. Here, the estimate for the EU group of studies (k = 2) of *r* = .29 was larger than for the studies from the US (k = 2) and other democratic countries (k = 2) which had estimates of *r* = .06 and *r* = .19 respectively, neither of which were statistically significant (*Q*_Between_ = 15.737, p = .000).

##### Ideology

Moderator analysis was used to assess heterogeneity between the ideological strain(s) to which effect sizes pertained. Studies were classified as examining nonspecific or mixed ideologies, Islamist, Left‐wing or Right‐wing ideologies based on the studies' descriptions. The between group analysis was performed for any factor which included at least two effect sizes from two or more of the categories. As such, the analysis was conducted on 49 factors pertaining to radical attitudes, 15 for radical intentions, and four for radical behaviors. For the factors pertaining to radical attitudes, statistically significant between‐group heterogeneity (p < .05) was found for 9 factors, and marginally significant between‐group heterogeneity (p < .10) for one additional factor. For the factors pertaining to radical intentions statistically significant between‐group heterogeneity (p < .05) was found for 5 factors, and marginally significant between‐group heterogeneity (p < .10) for two additional factors. For the factors pertaining to radical behaviors, two were found to have statistically significant between‐group heterogeneity. The results of the analysis are arranged below in Table 10 in alphabetical order. The findings indicate that while many factors may have 'universal' relationships with radicalization irrespective of ideology, some factors may have more unique relationships with different factors. These findings therefore need to be considered when risk and protective factors are applied in the context of risk assessment or counter‐radicalization interventions.

With respect to the outcome of radical attitudes, for Anomia, the estimate for nonspecific/mixed ideology (k = 2) was r = .24 and *r* = .27 for Right‐wing ideology (k‐2), with both presenting as larger than the estimate for Islamist ideology (k = 7), which was a marginally significant estimate of *r* = .13 (*Q*_Between_ = 7.618, p = .022). With respect to Gender (Male), the estimates for Left (k = 8) and Right‐wing ideologies (k = 4) were *r* = .16 and *r* = .17 respectively, presenting as larger than the estimates for nonspecific (k = 24) and Islamist ideologies (k = 20) which were *r* = .09 and *r* = .06 respectively (*Q*_Between_ = 25.183, p = .000). With respect to In‐Group Identity, the estimate for Islamist ideology (k = 13) of *r* = .08 presented as larger than the nonstatistically significant estimate of *r* = .01 for studies examining nonspecific (k = 10) ideologies (*Q*_Between_ = 5.300, p = .021). For Institutional Trust, the estimate for Islamist ideology (k = 5) of *r* = ‐.26 was significantly larger than the estimate for nonspecific ideologies (k = 4) of *r* = ‐.03 (*Q*_Between_ = 4.424, p = .035). For Law Legitimacy, the estimate for Islamist ideology (k = 6) of *r* = .49 was significantly larger than the estimates of Right‐wing ideology (k = 2) and nonspecific ideologies (k = 6) which were both *r* = .16 (*Q*_Between_ = 5.255, p = .072). With regard to Parental Abuse, the estimates for Islamist (k = 2) and Left‐wing (k = 2) ideologies of *r* = .17 and *r* = .16 respectively, were slightly larger than the estimate for Right‐wing ideology (k = 2) of *r* = .10 (*Q*_Between_ = 13.020, p = .001). For Perceived Discrimination, the estimate for nonspecific ideologies (k = 2) was *r* = .23, more than double the size of the estimate for Islamist ideology (k = 4) of *r* = .11 (*Q*_Between_ = 32.277, p = .000). With respect to Prayer Frequency, the estimate for Right‐wing ideology (k = 2) of *r* = .30 was significantly larger than the estimate for Islamist ideology (k = 3) of *r* = .06 (*Q*_Between_ = 27.503, p = .000). For Religiosity, the estimate for nonspecific ideologies (k = 7) was a statistically nonsignificant *r* = ‐.08, whereas for Islamist ideology (k = 8) it was a statistically significant *r* = .10. The estimate for Right‐wing ideology (k = 3) was even larger at *r* = .15, and although the estimate itself was not statistically significant (*Q*_Between_ = 6.262, p = .044). Lastly, with respect to being a Welfare Recipient, the estimate for nonspecific ideology (k = 2) was *r* = .03 and for both Islamist (k = 3) and Right‐wing (k = 2) ideologies was *r* = .04, although the estimate for the former was not statistically significant. The estimate for Left‐wing ideology (k = 2) was slightly larger at *r* = .09 (*Q*_Between_ = 15.112, p = .002).

With respect to the outcome of radical intentions, for Anger, the estimate for nonspecific ideologies (k = 2) was *r* = .11, larger than the estimates for both Islamist (k = 6) and Left‐wing (k = 3) ideologies of *r* = .46 and *r* = .31 respectively (*Q*_Between_ = 15.832, p = .000). For Collective Relative Deprivation, while between group heterogeneity was only marginally significant (*Q*_Between_ = 3.640, p = .056), the estimate for Islamist ideology (k = 12) of *r* = .37 was larger than the estimate for nonspecific ideologies (k = 3) of *r* = .22. For Commitment to a Cause, the estimate for nonspecific ideologies (k = 2) of *r* = .62 was larger than the estimate for Left‐wing ideology (k = 7) of *r* = .37 (*Q*_Between_ = 13.721, p = .000). With regard to In‐Group Identity, while between group heterogeneity was only marginally significant (*Q*_Between_ = 3.075, p = .080) the estimate for Islamist ideology (k=10) of *r* = .30 was larger than the estimate for nonspecific ideologies (k = 3) of *r* = .14. With regard to Moral Neutralizations, the estimates for Islamist ideology (k = 2) and Left‐wing ideology (k = 4) of *r* = .52 and *r* = .42 respectively, were significantly different than the nonsignificant estimate for nonspecific ideologies (k = 2) of *r* = .04 (*Q*_Between_ = 13.699, p = .001). Lastly, with respect to Openness, the estimate for Islamist ideology (k = 3) of *r* = ‐.33 was larger than the estimate for nonspecific ideologies (k = 7) of *r* = ‐.10 (*Q*_Between_ = 6.819, p = .009).

With respect to the outcome of radical behaviors, for Gender, the estimate for Islamist ideology (k = 2) of *r* = .67 was larger than the estimate for mixed ideologies (k = 2) of *r* = .24 (*Q*_Between_ = 5.181, p = .023). With respect to Marital Status, the estimate for Islamist ideology (k = 3) was *r* = .00, whereas for mixed ideologies (k = 3) it was *r* = ‐.06 (*Q*_Between_ = 7.000, p = .008).

##### Effect size derivation

Moderator analysis was used to assess the impact of the inclusion of supplementary effect sizes from the standardization of partial effect sizes. For the outcome of radical attitudes there were 23 factors that were analysed which included at least two effect sizes derived from such sources, in addition to having at least two effect sizes derived from bivariate sources. In addition, there were two such factors pertaining to the outcome of radical behaviors. There were no factors for the outcome of radical intentions. Table 11 displays the results with the factors arranged in alphabetical order. The findings indicate that in some instances, combining effect sizes from bivariate and multivariate sources can have an impact on heterogeneity and the results.

Between‐group heterogeneity was not statistically significant (p > .100) for all of the factors with the exception of two factors pertaining to radical attitudes. An additional factor was found to have marginally significant between‐group heterogeneity. For the factor of Anxiety, the estimate for bivariate effect sizes (k = 6) was *r* = .05, while for the standardized partial effect sizes (k = 2) was a statistically nonsignificant *r* = ‐.01 (*Q*_between_ = 4.184, p = .041). For Experiencing Discrimination, the estimate for the bivariate effect sizes (k = 12) was *r* = .09, while for the standardized partial effect sizes (k = 8) was a statistically nonsignificant *r* = .02 (*Q*_between_ = 11.425, p = .001). With regard to Institutional Trust, the estimate for bivariate effect sizes (k = 7) was *r* = ‐.23, while for the standardized partial effect sizes (k = 4) was a marginally significant *r* = ‐.07 (*Q*_between_ = 3.191, p = .074).

#### 6.3.5 Leave‐one‐out analysis

##### Radical attitudes

The leave‐one‐out sensitivity analysis found that a single study significantly contributed to heterogeneity for 13/27 protective factors and 28/61 risk factors for which it was possible to carry out the analysis (k > 3). For protective factors, heterogeneity was reduced to a moderate level (*I* ^*2*^ > 50) for Extraversion, to a low level (*I* ^*2*^ > 25) for Social Support, and to a very low level (*I* ^*2*^ < 25) for Parental Education Attainment. Heterogeneity was reduced to *I* ^*2*^ = 0 for 9 factors, namely Conscientiousness, General Trust, Parental Control, Political Participation, School Bonding, Teacher Bonding, and all three measures of Self‐Esteem (Personal, Public, and Group). Across all of these factors, the change in the size of the estimates ranged from .01‐.08. With regard to the estimate for Student Status, the direction of the estimate flipped from being negative (protective factor) to positive (risk factor). While the original estimate was a nonstatistically significant *r* = ‐.00, the adjusted estimate was .06 (p = .001) and heterogeneity was reduced to a large to low degree (Q = 1.299, p = .729, *I* ^*2*^ = 0). While Neuroticism also switched from negative to positive, both estimates were nonstatistically significant. For risk factors, heterogeneity was reduced to a moderate level (*I* ^*2*^ > 50) for 9 factors, a low level (*I* ^*2*^ > 25) for 4 factors, and a very low level (*I* ^*2*^ < 25) for 5 factors, and to *I* ^*2*^ = 0 for 10 factors. The largest change in an estimate was for Similar Peers, with a reduction equal to *r* = .10 in the size of the estimate.

One notable result is that for Anxiety, removing the study that contributed most to heterogeneity changed the direction of the estimate from a positive to a negative relationship of a similar order and magnitude. The estimate for APD also changed from positive to negative but remained statistically nonsignificant. Second generation immigrant also switched from positive to negative but both original and adjusted estimates were nonstatistically significant.

##### Intentions

For the outcome of radical intentions, the removal of a single study was found to contribute to a significant reduction in heterogeneity for 16 factors, made up of 3 protective and 13 risk factors. Heterogeneity was reduced to a moderate level (*I* ^*2*^ > 50) for Harmonious passion and External political efficacy, to a low level (*I* ^*2*^ > 25) for Neuroticism, Quest for Significance, Negative Affect, Self‐Esteem and In‐Group Superiority, to very low (*I* ^*2*^ < 25) for In‐Group Connectedness, and to *I* ^*2*^ = 0 for Conscientiousness, Outgroup Friendships, and Socioeconomic Status, Anomie, Individual Relative Deprivation, Identity Fusion, Positive Affect, and Power Distance Orientation. There were no notable changes in the interpretations of the estimates, with changes in the size of the estimates remaining small (*r* < .07).

##### Behaviors

For the outcome of radical behaviors, the removal of a single study significantly reduced heterogeneity for 8 factors, made up of 3 protective and 5 risk factors. Heterogeneity was reduced to a moderate level (*I* ^*2*^ > 50) for Marital Status and Anger, to a low level (*I* ^*2*^ > 25) for Thrill‐Seeking, Deviant/Radical Peers and Exposure to Violence, and to *I* ^*2*^ = 0 for Parental Involvement, Law Abidance, and Low Self‐Control. For Anger, there was a reduction in the size of the estimate equal to *r* = .12. However, unlike the original estimate, the adjusted estimate was found to be statistically significant (p < .05). Additionally, for the factor of Deviant/Radical Peers, there was a reduction in the size of the estimate equal to *r* = .16. The adjusted estimate of *r* = .18 would significantly reduce the relative position of this factor in the rank order of risk factors for radical behaviors, and downgrade it from a moderate sized relationship to a small one.

#### 6.3.6 Publication bias

Publication bias was assessed using two methods, the Trim‐and‐Fill method, and Egger's regression method. Table 13 displays the results of the analyses, with the factors being arranged in rank‐order according to the size of their pooled estimates. For the Trim‐and‐Fill method, the number of imputed studies is indicated by the column labeled “T&F” and the adjusted estimate and 95% confidence intervals are presented in the adjacent columns, followed by the *Q* statistic to assess heterogeneity. For Egger's regression method, the regression coefficient is displayed with the associated p‐value in parentheses.

**Table 13:** Publication bias analysis

Factor	*r*	k	T&F	*r*_*adjusted*_	95% CI	Q	Egger's test
							β1 (*p* value)
***Radical attitudes***							
*Protective factors*							
Student	−.00	5	1	−0.03	−0.13, 0.08	56.95***	−11.85 (.131)
Neuroticism	−.02	4	1	−0.05	−0.13, 0.03	40.94***	5.15 (.203)
SES	−.04**	27	2	−0.03**	−0.05, −0.01	256.44***	0.333 (.353)
Marital status	−.04***	11	2	−0.04†	−0.06, −0.02	17.41	−0.286 (.255)
Political efficacy	−.05	7	0	−	−	−	4.84 (.133)
Age	−.05**	46	5	−0.06*	−0.09, −0.03	1428.10***	1.22 (.169)
General trust	−.06**	4	2	−0.03†	−0.07, 0.01	16.82**	−1.84 (.048)
Education	−.07**	30	6	−0.09***	−0.13, −0.05	1022.53***	−2.02 (.058)
School performance	−.09**	8	0	−	−	−	−1.21 (.379)
Outgroup friends	−.09**	3	2	−0.11**	−0.15, −0.06	2.94	1.54 (.034)
Extraversion	−.10	4	1	−0.14	−0.30, 0.03	237.67***	4.03 (.401)
Parental academics	−.10***	4	2	−0.07	−0.13, −0.02	125.99***	2.33 (.280)
Openness	−.10†	4	0	−	−	−	2.81 (.372)
Parental involvement	−.10***	12	0	−	−	−	.462 (.420)
Self‐esteem (public)	−.11	3	0	−	−	−	−4.88 (.065)
Self‐esteem (group)	−.11	3	0	−	−	−	−5.26 (.069)
Social support	−.12***	7	0	−	−	−	1.92 (.098)
Conscientiousness	−.12***	4	1	−0.11***	−0.14, −0.09	4.67	1.15 (.305)
Parental control	−.12**	4	1	−0.13*	−0.20, −0.06	66.82***	3.07 (.359)
School bonding	−.13***	7	0	−	−	−	0.453 (.423)
Teacher bonding	−.13***	3	0	−	−	−	0.394 (.468)
Agreeableness	−.13*	4	0	−	−	−	−2.23 (.390)
Political satisfaction	−.15**	6	0	−	−	−	−7.26 (.107)
Self‐esteem (personal)	−.17**	3	0	−	−	−	−3.12 (.212)
Institutional trust	−.17***	11	3	−0.23***	−0.34, −0.11	3235.45***	−6.00 (.083)
Life satisfaction	−.19***	3	0	−	−	−	0.010 (.499)
Law abidance	−.55***	3	0	−	−	−	−3.47 (.405)
*Risk factors*							
Depression	.00	10	0	−	−	−	0.712(.394)
Immigrant	.01	23	0	−	−	−	−0.313 (.367)
2nd gen immigrant	.01	11	3	0.02	−0.01, 0.05	157.092***	−0.091 (.481)
Political participation	.01	4	1	0.03	−0.02, 0.07	11.782*	3.12 (.167)
Life events	.02	9	0				−0.63(.350)
Physical health	.02*	4	2	0.03**	0.01, 0.05	4.60	−1.34 (.094)
Moved residence	.03	4	1	0.02	−0.02, 0.06	22.19***	1.25 (.387)
Anxiety	.04†	8	0	−	−	−	1.84(.156)
APD	.03	4	0	−	−	−	1.371 (.298)
Alcohol use	.04†	5	0	−	−	−	−0.515 (.437)
Religiosity	.05	19	5	−0.02	−0.08, 0.04	626.34***	1.235 (.306)
West Vs. Islam	.05***	3	2	0.06***	0.04, 0.08	3.08	−3.61 (.209)
Unemployed	.05***	13	5	0.04***	0.02, 0.05	37.93***	1.139 (.028)
Welfare	.05***	9	0	−	−	−	−.624 (.328)
Worship attendance	.06*	13	0	−	−	−	2.05 (.136)
Uncertainty	.07**	10	1	0.05*	0.01, 0.10	46.75***	0.232 (.411)
In‐group identity	.07***	25	0	−	−	−	2.283 (.008)
Perceived injustice	.08*	8	2	0.04	−0.02, 0.11	62.54***	0.828 (.352)
Discrimination	.08***	21	1	0.08***	0.06, 0.10	78.24***	−0.865 (.106)
Experienced violence	.07***	16	0	−	−	−	−1.03 (.182)
Aggression	.09**	5	0	−	−	−	2.33 (.191)
Family violence	.10***	5	1	0.10***	0.07, 0.13	13.14†	−1.31 (.261)
Males	.10***	56	1	0.10***	0.08, 0.12	635.61***	.458(.703)
Online posting	.11***	4	0	−	−	−	1.43 (.328)
Indiv. Relative dep.	.11***	15	1	0.12***	0.07, 0.16	381.416	3.64(.043)
Drug use	.12*	6	0				−0.582 (.455)
Violent media	.12***	6	1	0.13***	0.08, 0.17	84.53***	1.08 (.393)
Personal strain	.13***	3	1	0.12***	0.08, 0.17	.58	0.171 (.434)
Parental abuse	.13***	6	1	0.13***	0.09, 0.16	23.48**	0.511 (.418)
Self‐efficacy	.13	5	0	−	−	−	5.43 (.327)
Anger	.14***	4	1	0.17***	0.09, 0.25	30.65***	2.01 (.099)
Prayer frequency	.14**	5	0	−	−	−	3.32 (.163)
Significance quest	.14***	10	4	0.09**	0.02, 0.16	43.04***	2.54 (.107)
Perceive discriminate	.15***	8	1	0.16***	0.11, 0.20	78.10***	−3.23 (.138)
Political grievance	.15***	8	0	−	−	−	0.505 (.436)
Dual identity	.15***	7	2	0.12***	0.05, 0.18	50.82***	0.414 (.408)
Segregationist	.15†	3	0	−	−	−	8.27 (.050)
Collect. Relative dep.	.16***	19	3	0.14***	0.10, 0.18	223.82***	0.245 (.434)
Disconnectedness	.16***	7	1	0.14***	0.06, 0.21	19.46**	0.959 (.251)
Deviant peers	.17***	12	0	−	−	−	−6.00 (.033)
Antidemocratic	.19***	8	2	0.17***	0.12. 0.21	101.87***	−1.90 (.179)
Anomia	.19***	12	0	−	−	−	−0.908 (.274)
SDO	.19†	5	0	−	−	−	5.906 (.116)
Juv. delinquent	.20***	7	0	−	−	−	4.02 (.258)
Low integration	.20***	21	7	0.26***	0.17, 0.35	3546.51***	3.31 (.000)
Self‐sacrifice	.20***	6	2	0.25***	0.15, 0.34	36.20***	−5.44 (.099)
Legitimacy	.22***	10	3	0.29***	0.18, 0.39	2379.61***	5.08 (.051)
Self‐control	.25***	8	0	−	−	−	−4.59 (.050)
Authoritarianism	.25***	21	3	0.31***	0.20, 0.40	3047.83***	2.71 (.287)
Radical media	.26***	5	0	−	−	−	6.43 (.161)
Criminal history	.29***	4	0	−	−	−	2.57 (.230)
Police contact	.30***	6	0	−	−	−	−4.55 (.197)
Thrill‐seeking	.31***	17	2	0.33***	0.24, 0.41	1761.33***	3.56 (.046)
Symbolic threat	.31***	6	2	0.34***	0.27, 0.40	17.14*	−0.24 (.438)
Similar peers	.31***	3	0	−	−	−	3.43 (.166)
Moral neutralizations	.32***	15	1	0.33***	0.24, 0.42	1470.35***	7.73 (.008)
In‐group superiority	.34***	13	1	0.32***	0.23, 0.40	404.40***	1.51 (.287)
Realistic threat	.35***	4	2	0.41***	0.33, 0.48	15.27*	−0.29 (.018)
Political extremism	.37***	6	0	−	−	−	13.68(.007)
Life attachment	.41**	3	0	−	−	−	1.86 (.459)
Machoism	.42***	4	0	−	−	−	.296 (.489)
***Radical intentions***							
*Protective factors*							
Education	−.03	12	1	−0.03	−0.08, 0.02	34.43***	0.11 (.459)
SES	−.03	11	2	−0.06	−0.11, 0.00	30.66***	1.18 (.176)
Age	−.08**	25	0	−	−	−	−0.79 (.244)
Outgroup friendship	−.10	3	2	−0.12**	−0.17, −0.07	4.34	1.59 (.182)
Agreeableness	−.12***	7	3	−0.13***	−0.15, −0.11	8.14	1.87 (.029)
Conscientiousness	−.13***	7	0	−	−	−	0.25 (.449)
Openness	−.16***	10	0	−	−	−	0.553 (.416)
Immigrant	−.22***	8	1	−0.23	−0.41, −0.09	130.62***	−4.56 (.037)
*Risk factors*							
Uncertainty	.05**	5	2	0.03†	−0.00, 0.07	8.94	4.06 (.010)
Neuroticism	.07***	8	2	0.05**	0.02. 0.09	23.29**	3.49 (.028)
APD	.08**	3	2	0.07**	0.02, 0.12	.949	0.612 (.019)
SDO	.09	5	1	0.13	−0.08, 0.32	129.81***	−8.65 (.234)
External efficacy	.09	4	0	−	−	−	7.57 (.068)
Males	.10***	28	3	0.09***	0.04, 0.13	165.20***	−1.55 (.045)
Significance quest.	.11***	4	1	0.12***	0.07, 0.17	4.55	−0.964 (.332)
Extraversion	.12***	8	0	−	−	−	−0.363 (.426)
Indv. Relative dep.	.14†	4	2	0.20***	0.09, 0.30	18.58***	−2.90 (.110)
Positive affect	.16**	4	1	0.18***	0.09, 0.27	8.13	−7.93 (.326)
Harmonious passion	.16*	5	0	−	−	−	−5.53 (.327)
Low integration	.18***	9	0	−	−	−	1.70 (.085)
Self‐esteem	.20*	6	0	−	−	−	2.65 (.094)
Dark triad	.20**	6	2	0.11	−0.03, 0.25	258.19***	9.46 (.132)
PDO	.23**	5	1	0.25***	0.15, 0.34	27.78***	−2.48 (.261)
In‐group connect.	.23***	6	1	0.21***	0.12, 0.30	15.59*	10.16 (.015)
Anomia	.25***	6	2	0.29***	0.19, 0.39	58.81***	−10.45 (.029)
In‐group identity	.25***	15	1	0.27***	0.17, 0.36	235.62***	3.56 (.081)
Realistic threat	.26***	6	0	−	−	−	.359 (.449)
Perceived injustice	.28***	13	0	−	−	−	5.49 (.020)
Symbolic threat	.29***	6	2	0.38***	0.20, 0.54	188.75***	3.23 (.148)
Coll. Relative dep.	.36***	17	2	0.39***	0.30, 0.47	167.54***	1.57 (.197)
Moral neutralizations	.36***	8	0	−	−	−	−4.53 (.297)
In‐group superiority	.37***	5	1	0.39***	0.32, 0.46	11.70†	−1.81 (.147)
Anger	.40***	12	0	−	−	−	6.36 (.014)
Commitment	.43***	9	0	−	−	−	−9.63 (.000)
Activist intent	.44***	18	1	0.46***	0.36, 0.54	349.71***	−4.22 (.111)
Negative affect	.47***	4	0	−	−	−	−21.39 (.123)
Radical attitudes	.48***	12	3	0.43***	0.33, 0.52	318.75***	−0.470 (.430)
Obsessive passion	.50***	5	1	0.45***	0.26, 0.61	66.08***	−7.45 (.354)
Identity fusion	.52***	3	0	−	−	−	−20.58 (.230)
***Radical behaviors***							
*Protective factors*							
Marital status	−.03	6	0	−	−	−	−1.64 (.113)
Education	−.04	13	0	−	−	−	−4.42 (.102)
Parental involvement	−.06***	4	1	−0.06***	−0.09, −0.04	−	0.252 (.464)
Age	−.10*	8	0	−	−	−	−2.92 (.226)
School bonding	−.11***	4	1	−0.11***	−0.12, −0.09	.97	−0.220 (.420)
Law abidance	−.22***	3	0	−	−	−	−1.50 (.358)
*Risk factors*							
Bullied	.04***	4	1	0.04***	0.02, 0.05	1.30	−1.03 (.137)
Immigrant	.05	5	2	0.12*	0.02, 0.21	162.51***	−8.45 (.081)
Welfare	.06*	6	0	−	−	−	0.643 (.418)
Abused	.07***	5	2	0.06***	0.05, 0.08	5.30	1.38 (.057)
Experienced violence	.11***	5	1	0.10***	0.06, 0.14	20.93***	2.32 (.232)
Personal injustice	.15***	3	0	−	−	−	−2.84 (.240)
Unemployed	.19***	7	0	−	−	−	8.29 (.083)
Thrill‐seeking	.19***	6	1	0.20***	0.17, 0.23	25.52**	2.17 (.237)
Anger	.20	3	0	−	−	−	−28.12 (.309)
Self‐control	.28***	3	0	−	−	−	−27.84 (.228)
Deviant peers	.30**	4	1	0.35***	0.17, 0.51	395.75***	17.75 (.153)
Radical attitudes	.30***	11	1	0.32***	0.21, 0.42	988.67***	2.23 (.379)
Past military	.33†	3	0	−	−	−	−26.91 (.204)
Criminal history	.35**	4	1	0.42***	0.17, 0.62	658.46***	6.41 (.350)
Gender	.39**	7	0	−	−	−	14.54 (.306)

Regarding the outcome of radical attitudes, publication bias analysis was possible for 88 factors (*k* > 3). The Trim−and‐Fill method found between 1 and 7 imputable effect sizes for half of the factors, namely 12 protective and 32 risk factors. Of these factors, 7 of the protective factors and 9 of the risk factors were also found to have statistically significant (*p* < .10) evidence of publication bias according to Egger's regression method. Among the 14 protective and 29 risk factors for which there were no imputable effect sizes, Egger's regression coefficient was found to be statistically significant for 5 of the risk factors. The analysis indicates the possible presence of publication bias in as much as half of the factors included in the analysis. However, the largest change in an estimate using the Trim‐and‐Fill method was *r* = .07, and the relative magnitude of the estimates were not affected.

Regarding the outcome of radical intentions, publication bias analysis was possible for 39 factors (*k* > 3). The Trim‐and‐Fill method found between 1 and 3 imputable effect sizes for 5 protective and 19 risk factors. Of these factors, 2 of the protective and 7 of the risk factors were also found to have statistically significant (*p* < .10) Egger's regression test intercepts. In addition, for six risk factors for which the Trim‐and‐Fill method did not identify missing studies, Egger's regression test was found to be statistically significant. The analysis indicates the possible presence of publication bias in a large proportion of the factors analysed. However, evidence of publication bias having a significant impact on the estimates was found for only a small number of factors. Even in these cases the overall influence over the pooled estimates appears to be relatively small. The most significant impact was found for Symbolic threat, with which two imputed studies led to an estimate of *r* = .38, up from an original estimate of *r* = .29.

Regarding the outcome of radical behaviors, publication bias analysis was possible for 21 factors (*k* > 3). The Trim‐and‐Fill method found between 1 and 2 imputable effect sizes for 2 protective and 8 risk factors. Of these factors, statistically significant (*p* < .10) evidence of publication bias according to Egger's regression method was found for only 2 of the risk factors. In addition, a statistically significant regression coefficient was found for 1 additional factor for which the trim and fill method did not identify missing studies. The analysis indicates the possible presence of publication bias in about half of the analysed factors. However, the largest impacts for the Trim‐and‐Fill were only of the order of a *r* = .07 change in the estimate.

## 7 DISCUSSION

### 7.1 Summary of main results

#### 7.1.1 Overview

This review had two primary objectives. First, to identify what the putative risk and protective factors for radicalization are, without making any pre‐determinations, and second, to identify the relative magnitude of the estimates for the different factors. In doing so, the review also sought to identify consistencies in the clustering of estimates for factors that are conceptually or theoretically related, as well as consistencies across the different outcomes. Given the large number of factors analysed and the nature of these objectives, it is not possible to provide an in depth discussion on the relevance of each of them. However, the way in which certain factors tend to cluster together within the rank orders, and which tiers they tend to fall in, provide for some important findings in and of themselves. Below we highlight the key findings and also discuss some of the consistencies and differences that were identified across outcomes, as well as between regions and ideologies.

##### Radical attitudes

**Figure 6:**
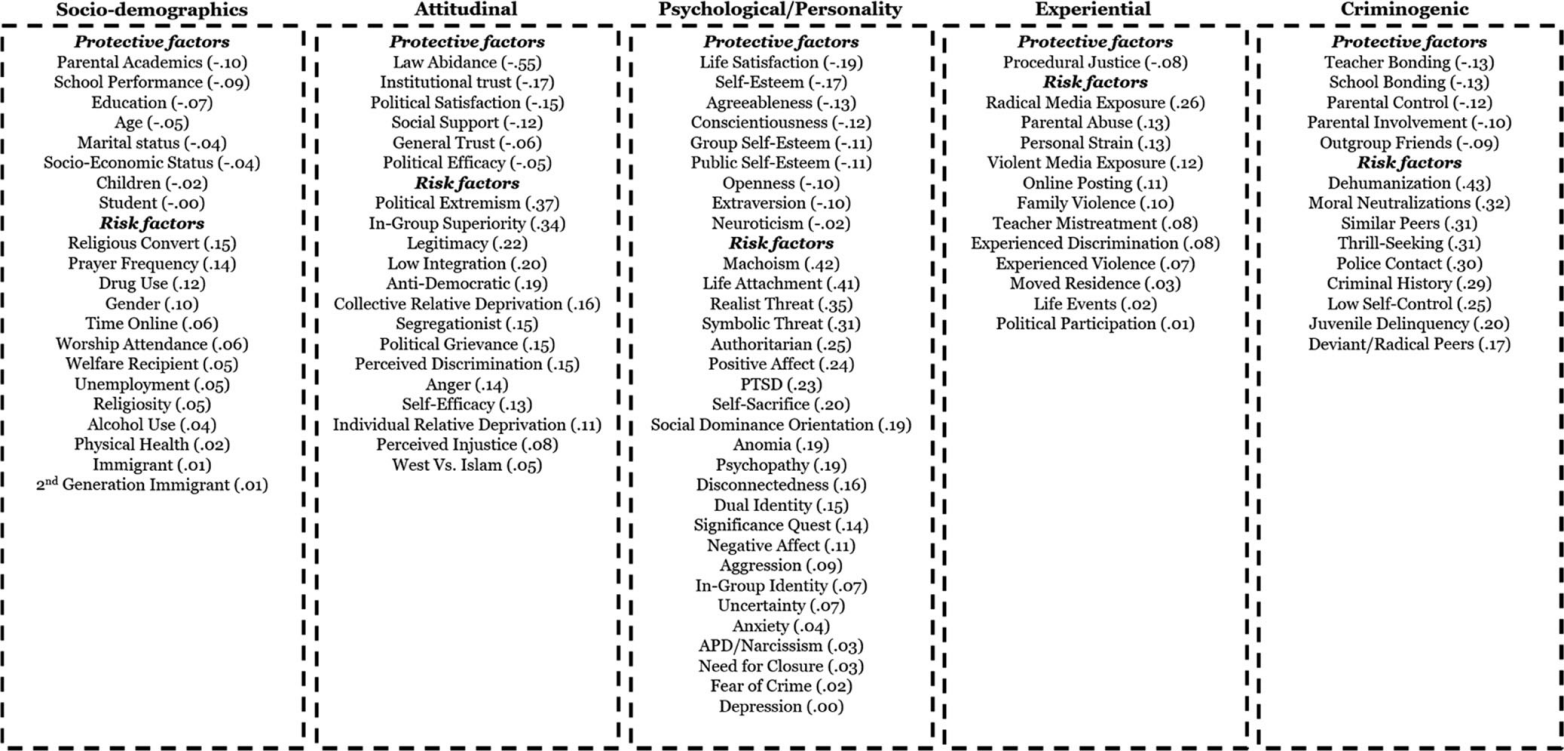
Distribution of factors associated with radical attitudes across five domains with mean effect size (*r*) in parentheses

For the outcome of radical attitudes, 100 risk and protective factors were identified. The factors can broadly be categorized into five domains, namely: (1) Individual background and sociodemographic factors, (2) attitudinal factors, (3) psychological and personality related factors, (4) experiential factors, and (5) Traditional criminogenic factors. While factors from each of these categories spanned all of the tiers, some general trends were observed. Individual background and socio‐demographic factors consisted entirely of very small‐small estimates. Experiential factors too had estimates that fell within the range of very small‐small estimates. With but a few exceptions, attitudinal factors were also associated with very small‐small estimates. On the other hand, psychological and personality related factors, as well as criminogenic factors, included estimates that spanned all tiers. All of the moderate sized factors, with the exception of three attitudinal factors, came from these two domains (Figure 6).

In terms of traditional criminogenic factors, the literature has previously pointed to significant overlaps between the risk and protective factors for radicalization and ordinary crime and delinquency (e.g., Lösel et al., 2018). The results highlight that factors known to be predictive of criminal attitudes (e.g., Walters, 2015, 2017) also have relatively large relationships with radical attitudes. The criminogenic factors also point to the utility and relevance of criminological perspectives for understanding and explaining radicalization (LaFree et al., 2020). For example, Juvenile Delinquency, Criminal History, Contact with the Police, Low Self‐Control, Thrill‐Seeking/Risk‐Taking, Similar Peers, Deviant/Radical Peers Moral Neutralizations (and Dehumanization) are factors associated with Self‐Control Theory, Social‐Control Theory and Social Learning Theory, each of which have been suggested as useful frameworks for understanding radicalization (Figure 6).

**Figure 7:**
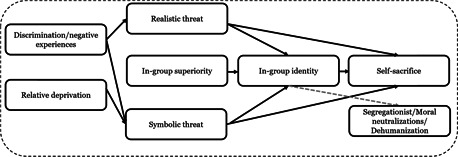
Conceptual relationship between Integrated Threat Theory and associated factors

With respect to psychological and personality related factors, factors with the largest relationships with radicalization can be found to relate to a small number of key perspectives, between which there is also a significant degree of overlap. For example, Self‐Sacrifice, In‐Group Superiority, and both Symbolic and Realistic Threat are all derived from Integrated Threat Theory (ITT; Stephan & Stephan, 2000). According to this perspective, in‐group members hold certain views of out‐group members that are anticipatory of the latter behaving in ways that are detrimental to the former. They also hold that their group have a superior system of beliefs, morals, and standards. That the out‐group provides an alternative system which also claims superiority is a source of symbolic threat to the group's superiority. When in‐group members, or the group as a whole, suffer from some form of deprivation or discrimination, they may view it as a realistic attempt by the outgroup to threaten the in‐group's existence. In line with these views, the in‐group members are likely to hold highly segregationist views, and to have a strong sense of attachment and identity with the in‐group. These factors therefore have a cumulative and interactive effect in developing attitudes that are supportive of the willingness to self‐sacrifice for the in‐group, including the use of violence. Moreover, collective relative deprivation and the emotional uncertainty it can lead to—two other risk factors identified—can increase perceived in‐group superiority (Trip et al., 2019). These factors are also conceptually linked to Dehumanization and Moral Neutralizations, in which an individual views another individual or group as lacking human qualities, or inherently being evil and therefore deserving of aggression towards them (Bandura, 1999) (Figure 7).

**Figure 8:**
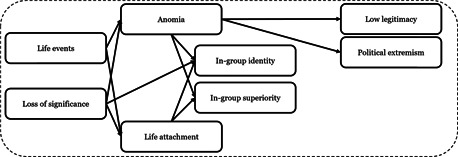
Conceptual relationship between “negative worldview” related factors

A diverse literature has described a range of overlaps, inter‐correlations and relationships between other psychological and personality related factors identified in the review. For example, secure “Life attachment” measures the degree to which an individual feels safe, certain, included, fairly treated, and having basic needs met. When these domains are negatively impacted, weakened, or even threatened, an individual may turn to nonnormative courses of behavior to restore them (Ozer & Bertelsen, 2019). The lack of a secure life overlaps with Anomia, which at the individual level represents a sense of social and political powerlessness, deprivation, social isolation and disconnectedness, and a failure to find meaning in institutionalized norms; or lack thereof (Smith & Bohm,^2008^). As a psychological state, Anomia can be induced by a Loss of Significance or other Life Events (Adam‐Troijan et al., 2019). As a result, an individual in a State of Anomia may seek out to re‐assert their identity and connectedness with the in‐group (Scheepers et al.,^1992^). McDill (1961) previously proposed that anomia, authoritarianism, and ethnocentrism (in‐group identity and superiority) were all dimensions of a negative worldview. There are many other possible inter‐correlations and interactions between these factors and others found in this tier. For example, negative worldview has been found to mediate the relationship between violations of beliefs or goals (as in the case of the factors associated with threat theory) and PTSD symptoms (Park et al., 2012), another factor in this tier of our results. Additionally, anomia and ethnocentrism are known to negatively impact factors like legitimacy. In turn, anomia and legitimacy are also known to increase the likelihood of political extremism. Van Damme and Pauwels (2012) find that these factors all predict support for vigilantism (Figure 8).

**Figure 9:**
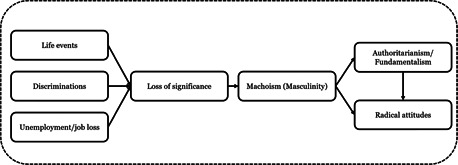
Conceptual relationship between masculinity related factors

Similar relationships exist between such factors and Machoism, or norms of exaggerated masculinity, which includes elements of pride, honor, and toughness. Masculinity, and masculine subculture values consider violence as a legitimate and even valued mode of behavior. Masculinity has previously been found to have a robust relationship with both criminal attitudes and behaviors (Walters, 2001). When masculinity is seen as being threatened, especially in situations that lead to a loss of significance, such as discrimination, job loss, or other life events, the individual may seek to reaffirm their masculinity (Bhui, Dinos, et al., 2012; Leander et al., 2020). This could promote the development of more authoritarian and fundamentalist views—another risk factor featured in the top tier— as well as radical attitudes (Bhui et al., 2012), and radical behaviors (Leander et al., 2020). There is therefore a potentially strong inter‐correlation between Machoism and other key risk factors (Figure 9).

These findings highlight that while a loss of significance and, or the resulting quest for significance, may not have the largest relationship with radicalization in and of itself, it does play a mediating role for other key factors (Kruglanski et al., 2014, 2015). The role of loss of significance and the quest for significance in radicalization has been demonstrated extensively in studies from a variety of contexts excluded from this review (such as Sri Lanka) and using a variety of proxies for radicalization that fall outside the scope of this review (Such as a willingness to self‐sacrifice, see Webber et al., 2018). In short, the theory holds that individuals who experience a loss of significance, which can occur for a variety of reasons, as discussed here, will seek to restore that significance, and that violence may be legitimized as a possible means to restoring significance (Kruglanski et al., 2014, 2015). This perspective demonstrates significant overlap with perspective from criminology concerning criminogenic needs of status, significance, or thrills (Clarke & Newman, 2006; Lloyd & Kleinot, 2017).

Another consistency among these factors can be found by contrasting them with the findings on the protective factors. For example, while low legitimacy has a robust relationship with radical attitudes, the negative relationship identified for Law abidance is even larger. Similarly, while Anomia has a strong relationship with radical attitudes, small but significant negative effects were found for factors such as General trust, Social support, Political satisfaction and Self‐esteem (individual), and Institutional trust. Additionally, while considerably smaller in the size of their estimates, some of the most important protective factors for radical attitudes included factors associated with Social‐control/Social bonding theory, namely: School performance, out‐group friendships, Parental involvement and control, and Teacher and school bonding.

##### Radical intentions

**Figure 10:**
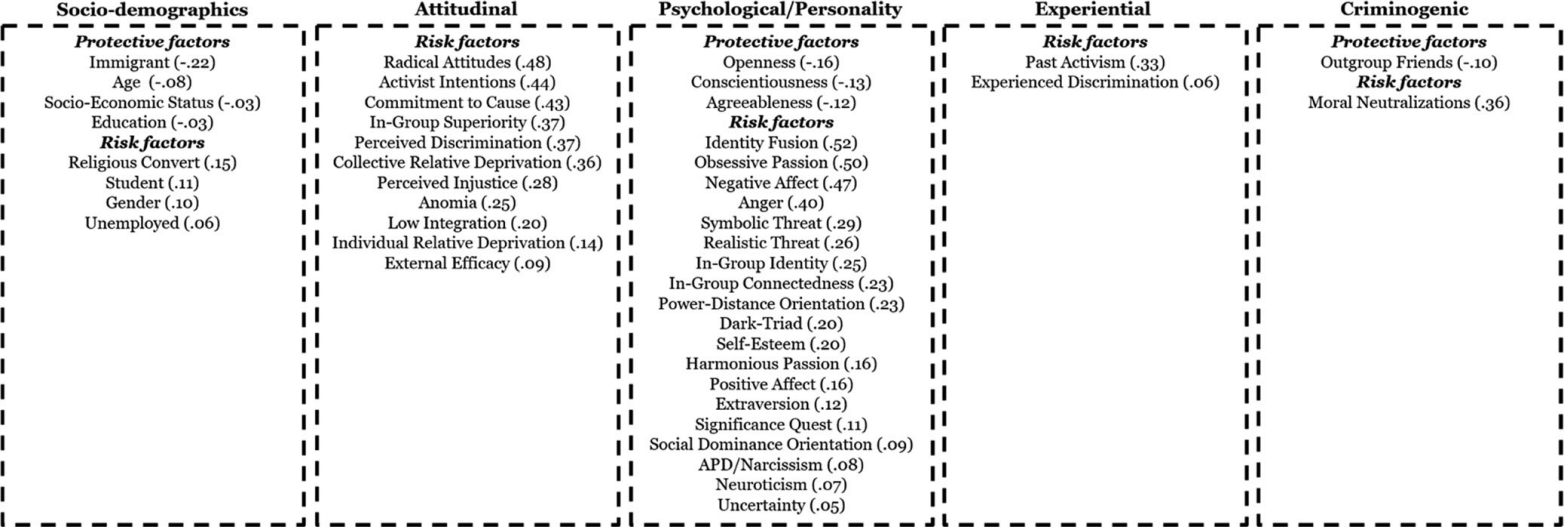
Distribution of factors associated with radical intentions across five domains with mean effect size (*r*) in parentheses

While there is considerable conceptual overlap between radical attitudes and intentions, the literature has focused on different factors in the study of these outcomes. For example, very few criminogenic factors were identified for the outcome of radical intentions. While there is some consistency between the findings for radical attitudes and intentions, there are also some key differences. One area in which there is a degree of consistency between the findings for radical attitudes and intentions is with respect to individual background and sociodemographic characteristics, which continued to be represented by very small‐small estimates. Additionally, psychological and personality related factors were dominant among the factors with the largest estimates. One key difference pertains to attitudinal factors, which overall had much stronger relationships with radical intentions than with radical attitudes. Additionally, the analysis for radical attitudes identified a number of personality factors which were not found for radical attitudes (Figure 10).

For example, the analysis of radical intentions highlights the relative importance of the *Dark‐Triad* set of personality factors, made up of Narcissism, Machiavellianism, and Psychopathy (Paulhus & Williams, 2002), and Power‐Distance orientation. While the analysis for radical attitudes included separate measures for Narcissism (APD) and Psychopathy, the estimates were in the lower tiers and were also found to not be statistically significant. The Dark‐Triad set of personality traits are known to be risk factors for a range of criminal and criminal analogous outcomes (Stellwagen, 2011). Specifically, Narcissism is known to be a key dynamic factor that affects criminal and violent attitudes. Narcissism has a strong positive correlation with Extraversion and Openness, and a strong negative correlation with Agreeableness. Similarly, Psychopathy has a strong correlation with Extraversion and Openness, and a strong negative correlation with Agreeableness, Conscientiousness and Neuroticism. Machiavellianism has a strong negative correlation with Agreeableness and Conscientiousness (Paulhus & Williams, 2002).

The review identifies consistencies in these findings as it relates to radicalization, with small but statistically significant negative (protective) estimates for Agreeableness, Conscientiousness and openness on radical intentions.

There is also considerable overlap between the Dark‐Triad and a negative worldview. It is said that individuals high in Machiavellianism have a strong tendency towards a negative worldview, are distrusting and suspicious of others' intentions, and expect others to pose a danger to them and generally have expectations that others always expecting the worst from other people (Christie & Geis, 1970). As noted above, such views and perceptions are highly correlated with other factors such as in‐group superiority. Machiavellianism also refers to the need to develop and defend one's position of power. This is especially the case when it comes to politically and socially oriented orders, which ties in to the other unique factor found in this tier, Power distance orientation. PDO relates to the structure that exists between those in power and those who are subordinate to them. In the social structure, the differential reactions of those of lower rankings toward those in authority is dependent on the degree of the power distance that characterizes the specific culture or society. Greater power distance orientations indicate a greater belief that relevant authorities should make decisions and lower standing individuals should follow those decisions (Travaglino & Moon, 2020). As such, there is considerable overlap between PDO and Authoritarianism.

With regard to negative affect, or negative emotions, the literature highlights that this emotional state is generally brought on by various forms of strain, as emphasized in strain theory (Agnew, 2006). Experiences and perceptions of discrimination and injustice can be a source of strain that increases negative emotions. In line with the Theory of Planned behavior, Ajzen and Fishbein (2005) suggest that emotions and even anticipated emotions contribute significantly to attitudes and intentions towards a given behavior. It has previously been suggested that when individuals suffer from strains, they anticipate the ensuing negative affect. In turn, they may turn to deviant outlets in order to avoid the negative affect (Brezina, 1996). This finding lends support to recent calls to better integrate the role of emotions in the study of radicalization (Rice & Agnew, 2013; Rice, 2009).

The top tier of factors also included a number of factors that directly pertain to attitudes towards a cause for which one may express intentions or willingness to defend using violence, namely: Commitment to the cause, Activism intentions, Radical attitudes and Obsessive passion. According to the dualistic model of passion there are key differences between the harmonious and obsessive variants of passion (Vallerand et al., 2003). Harmonious passion refers to a situation in which the individual places great value on the activity but is able to integrate the activity or cause into their lives in a way in which is strikes a balance with other important activities and things. In such a situation, while the activity may form an important part of the individual's identity, it is not necessarily the defining feature it. On the other hand, obsessive passion refers to a situation in which an individual attaches the activity (or cause) to their identity. The degree of fusion can be to the point when an individual's self‐worth is entirely tied up with the activity or cause (Vallerand et al., 2003). In such situations, participation in the activity is needed in order to full‐fill the individual's psychological and emotional needs. As such, Obsessive Passion could have a strong relationship with In‐group identity, Identity fusion, and In‐group superiority. The estimate for Obsessive passion was more than three times the size of the estimate for Harmonious passion, and was positioned side‐by‐side with identity fusion in the rank order. Having a strong identify as an “activist” may be indicative of obsessive passion or identity fusion. However, it can also indicate the extent to which an individual is committed to a particular cause or to engaging in actions on behalf of a cause.

One of the most commonly employed measures for radical intentions is Moskalenko and McCauley's (2009) ARIS. Already when the instrument was first validated, it was identified that there were significant differences between Activism and Radicalism in terms of the types of behaviors to which they refer, and the types of factors that tend to predict them. These findings, which have since been repeated in many studies, underpin the distinctions between Radicalism and Activism behaviors in McCauley and Moskalenko (2017) Two‐Pyramid model. However, there is still a high correlation between activism and radicalism. In their original study, Moskalenko and McCauley (2009) found that previous activism was a better predictor of radical intentions than previous radicalism was. Some perspectives on radicalization support the idea of a progression model, in which individuals and groups progress from legal, nonviolent activism to violent, radical forms of behavior. While we don't suggest that our findings stemming from correlational data support such a position, it is clear that there remains a strong correlation between activism and radicalism intentions and it may be necessary for future research to identify which risk and protective factors differentiate between those displaying these differential outcomes.

Lastly, the findings highlight the strong correlation between radical attitudes and intentions, demonstrating the close relationship between these two cognitive outcomes of radicalization.

##### Radical behaviors

Compared to the outcomes of radical attitudes and intentions, a relatively small number of studies and factors were identified for radical behaviors. Nevertheless, following the above discussions concerning radical attitudes and intentions, a considerable degree of consistency can be found in the factors identified as having the strongest relationship with this outcome. Factors such as Thrill‐Seeking/Risk‐Taking, Low Self‐Control, Radical Attitudes, Deviant/Radical peers, Radical Media (active engagement), Criminal History, and Prior Incarcerations, were among the factors with the largest relationships with radical behaviors. The findings demonstrate a degree of consistency with those for the outcome of radical attitudes, in which traditional criminogenic factors present as among the most salient risk factors.

One area in which the findings diverge from those for the cognitive outcomes of radical attitudes and intentions is with respect to individual background characteristics and experiential factors. Here, Gender (Male), Unemployment, Recent Job Loss were found to be salient risk factors for radical behaviors. Additionally, Current and Previous Military Experience, although statistically not significant, had large estimates. The findings regarding Gender appear to indicate that while it may be less important for predicting who may hold radical cognitions, Males are far more likely to engage in radical behaviors. Additionally, as per the above discussion concerning factors for radical attitudes, Recent Job Loss as well as Unemployment more generally can have an effect on a large range of other factors, such as engendering a loss of significance (Jasko et al., 2016). It is also important to note that the size of the estimate for Unemployment was almost four times the size as the estimate for radical attitudes and intentions. These findings indicate that Unemployment can pose a significant risk for those who already hold radical attitudes.

**Figure 11:**
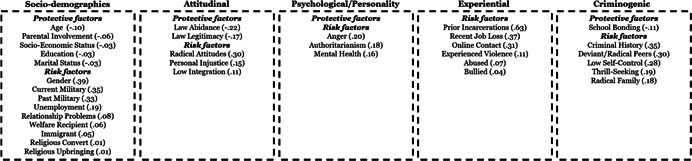
Distribution of factors associated with radical behaviors across five domains with mean effect size (*r*) in parentheses

Lastly, the finding that radical attitudes figures among the largest estimates for radical behaviors, as it did for radical intentions. As discussed above, while few justifiers and supporters of radical violence will ever actually engage in it, most of those who do engage in radical behaviors hold radical attitudes. While this highlights the relevance of the attitude‐intentions‐behavior consistency in the context of radicalization, the lack of an estimate concerning the role of intentions on behaviors—which are considered more proximal—represents a key gap in the knowledge. Nevertheless, it is worthwhile to highlight that Clemmnow et al (2020) found that 64% of the 125 lone‐actor terrorists in their dataset had been known to have expressed intentions to hurt others. This is compared to 12.7% of the general population surveyed, of which only 7.4% had made such expressions in the previous year. This would be equivalent to an effect size of *r* = .57, more than double the size of the effect size for attitudes (Figure 11).

##### Consistencies across outcomes

In general, when analysing the cognitive and behavioral outcomes of a given phenomenon, effect sizes for the same factors tend to be larger for the attitudinal outcomes, and smaller for the behavioral. This may be due to the fact that there is a higher prevalence of the attitudinal outcome than there is of the behavioral outcome (Bosco et al., 2015). This is certainly the case for radicalization. Large scale surveys such as the PEW Global Values Survey and the European Values Survey—which both figure in the data of studies included in this review—have found that approximately 5% of respondents may justify terrorism. However, as noted above, <1% of cognitively radicalized individuals will ever engage in radical behaviors. One implication therefore of comparing estimates across outcomes is that when an estimate goes against the above noted trend, and is found to be larger for the behavioral outcome, it should be given attention as a potential factor that could explain the move from cognitive to behavioral radicalization (Bosco et al., 2015). Three factors that stand out in this regard are Unemployment, Gender, and Deviant/Radical peers, which were found to be considerably larger for radical behaviors than for the two cognitive outcomes. These factors should therefore be given special attention by policy makers.

There were also some key differences in the estimates for factors between the outcomes of radical attitudes and intentions. As noted above, radical intentions are considered to be more proximal to radical behaviors than attitudes are. Estimates for the following factors were found to be considerably larger for radical intentions than attitudes: Anger (almost 3 times larger), Perceived injustice (3.8 times larger), Collective relative deprivation (more than double), Negative affect (almost 5 times larger), In‐group identity (3.5 times larger).

##### Consistencies across contexts and ideologies

With the exception of a few factors, the review found that the different ideological strain studies examined, as well as the region from which samples were derived, had no impact on the pooled estimates. This suggests that many of these factors may have global relevance for different types of radicalization in different types of OECD countries. However, some notable differences were identified.

With respect to radical attitudes the only significant difference by region was for moral neutralizations, which had a larger estimate for US based studies. Between ideologies, the estimates for the following factors were largest for studies measuring right‐wing ideological strains: Parental academic achievement (Protective), Anomia, Gender, Prayer frequency, Religiosity. The largest estimates Welfare recipient was for studies measuring Left‐wing ideological strains. The largest estimates for Low legitimacy and immigrant status (Protective) were for studies measuring Islamist ideological strains. The largest estimate for Perceived discrimination was for studies measuring no specific/mixed ideologies.

With respect to radical intentions, larger estimates for Anger and Moral Neutralizations were found for studies examining both Left‐wing and Islamist ideological strains. The largest estimate for Openness (Protective) was found for studies examining Islamist ideological strain. The largest estimates for Commitment to a Cause, In‐Group Superiority, and Socio‐Economic Status (Protective) were found for studies measuring no specific/mixed ideologies.

With regard to geographic region, for radical attitudes the largest estimate for Moral Neutralizations was for studies based in the US. For radical intentions, the largest estimates for Commitment to the cause and Self‐esteem were for studies based in the EU. As noted above, for radical behaviors, the estimate for Unemployment was largest for studies from the EU.

Additionally, our analysis found that for a small number of factors, the estimates were effected by study‐level characteristics, namely year of data collection, the average age of samples, and the proportion of males in a sample.

Taken collectively, the results indicate that many risk factors may have universal relationships with radicalization outcomes in democratic countries and across ideologies, and be equally applicable across certain demographics. However, for those factors for which significant heterogeneity was found across such factors, these differences need to be taken into consideration when such factors are used in applied contexts, such as risk assessment.

##### Protective factors

A degree of consistency is found between risk and protective factors that capture opposing dimensions or similar or related constructs. For example, with respect to radical attitudes, a robust risk estimate was found for Low Life satisfaction, whereas a small but salient protective estimate was found for Life attachment. Similarly, whereas Low legitimacy presented a salient risk estimate, Law abidance had a large protective effect. Additionally, whereas positive parental bonds and out‐group friendships had salient protective estimates, factors pointing to familial instability, such as family violence and abuse, as well as Similar peers had salient risk estimates. Similar consistencies were found across a number of other factors as well.

However, we must keep in mind that due to the nature of the data and the specific analyses conducted in this review when interpreting the results concerning protective factors. According to Lösel and Farrington (2012), whereas direct protective factors are associated with a decreased risk of the deviant outcome, buffering factors predict a decreased risk in the presence of a particular risk factor. As such, the protective factors examined in this review only provide evidence concerning direct effects. Nevertheless, the identification of a relatively large number of protective factors in this review supports the integration of protective factors into risk assessment in particular, and interventions as well (Lösel & Farrington, 2012; Lösel et al., 2018).

##### Effect sizes

Overall, our results are in line with Lösel and Bender's observation (2006) that correlations for risk factors pertaining to deviant outcomes typically range between 0.1 and 0.2, except for antecedent externalizing behaviors,. However, as has been noted previously, even these small correlations may not be trivial and could have important policy implications (Lipsey & Wilson, 2001; Lipsey & Derzon, 1998). Nevertheless, it is likely that radicalization is similar to other deviant outcomes, in which risk factors operate both cumulatively and interactively. As discussed above, some risk factors may increase the likelihood of other risk factors developing. As such, even risk factors with relatively small relationships could compound the risk for radicalization.

##### Implications for policy

An important component of the counter‐radicalization toolkit is risk assessment. Unfortunately, the selection of factors upon which assessment is made may not be evidence‐based (Scarcella et al., 2016), and current tools are relegated to nominal scaling approaches (Klausen et al., 2016; Lösel et al., 2018). The findings from this review provide for the possibility for developing the evidence needed to move towards more robust, evidence‐based risk assessment tools that assign weights to the items they include (Silke, 2014). For example, the VERA‐2R risk assessment tool includes a domain of attitudinal factors that includes: rejection of democratic society and values, feelings of hate, frustration, persecution, alienation, and being a victim of injustices, in the same domain (Hart et al., 2017). The ERG22+ risk assessment tool includes a number of factors in its “engagement” domain, including: Identity & belonging, Need for status, and Excitement/adventure. While, the results of this review find all of these factors to feature among the risk factors analyzed, the magnitude of their effects vary considerably, indicating that they should be assigned relative weights.

The results of this review may also serve for informing the development of tailored approaches based on the type of radical ideology that is being dealt with (e.g., Right‐wing, Islamist, etc.). For example, with significant differences found in effect sizes across ideological strains, it may be that interventions targeting anomic conditions may be better suited for combatting right‐wing radicalization, whereas interventions seeking to improve legitimacy may be more effective in combatting Islamist radicalization. Similarly, risk assessment tools that include factors such as these may benefit from adjusting the weights assigned to the factors depending on the specific ideology of the subject being assessed. Taking such an approach would enable a move towards a *risk‐needs‐responsivity* model, in which the individual should be assessed based on the specific risk factors they present, their individual needs with respect to those factors, and as such, what types of treatments would be best suited for them in order to reduce their risk (Dean, 2016; Mullins, 2010).

Lastly, the review highlights that there are few differences in the relationships between identified risk factors and radicalization across geographic region (for democratic countries). This means that there is a strong potential for countries to learn from each other's approaches and experiences. However, where differences do exist, countries should consider tailoring their approaches to tackle risk factors that may have context‐specific effects. For example, the effect for unemployment on behavioral radicalization is considerably larger in the EU than in the United States. While we cannot discount the possibility of confounding, the finding provides an indication that employment‐oriented interventions may be especially useful in the EU context, and as such experimental evaluations would be warranted.

In terms of interventions, for some of the risk factors identified by the review, ideas can be drawn from other fields, including criminology, psychology, and education. For example, self‐control improvement programs in crime and justice have been found to be effective in improving self‐control and reducing the likelihood of a range of deviant outcomes (Piquero et al., 2016). Similarly, factors associated with moral disengagement were found to rank among the most important risk factors. Such factors may be improved with pedagogical interventions that employ *critical thinking* as an active ingredient (e.g., Bustamante & Chaux, 2014), an approach has already been implemented to combat radicalization in Indonesia (Taylor et al., 2017). Critical thinking interventions are best carried out when couched within *transformative learning* programs. Transformative learning is a method in which the learner is presented with a moral dilemma that “forces him/her to reconsider his/her taken‐for‐granted values and assumptions” (Taylor et al., 2017, p. 199). One example of a critical thinking activity includes having students comment and reflect on a scenario to identify instances and processes of moral disengagement, identifying moral disengagement in themselves, and coming up with alternative solutions (e.g., Bustamante & Chaux, 2014).

Sports based interventions have also been found to reduce moral disengagement. They can also serve to reduce the effects of deviant peers (Spruit et al., 2018), which this review found to be another important risk factor for radicalization outcomes. While sports programs are already being used as counter‐radicalization interventions, they have a tendency to target the less relevant factors of integration and identity. They also may promote parental bonds, which this review found to be a protective factor (e.g., Johns et al., 2014).

As discussed below, caution must be taken in extrapolating the results of this review to interventions, as the studies included in this review were observational in nature. Additionally, we do not intend any of the above to represent any specific policy recommendations. Rather, given the overwhelming focus on integration‐oriented interventions, and a lack of evaluation studies, we believe that exploring ways to target important risk factors identified in this review, including through evidence‐based methods with demonstrable effectiveness, offer promise.

### 7.2 Overall completeness and applicability of evidence

The searches for this review were conducted across a broad range of databases, with extensive supplementary searches conducted on Google scholar, and through cross‐referencing of the bibliographies from our 123 included studies. In order to ensure completeness, we also contacted leading researches, whom, as mentioned above, did provide a number of studies that our searches had not originally captured.

The studies included in this review span a broad range of factors. Samples were quite heterogeneous in terms of their outcome measures, ideological strain examined, and country of origin. The review included studies that utilized both validated instrument for measuring radicalization, as well as originally developed measures. The studies included in the review examined radicalization from across the spectrum of radicalizing ideologies, including Right‐wing, Left‐wing, Islamist, Ethno‐nationalist, and nonspecific ideologies/mixed ideologies. The review's inclusion criteria limited study eligibility to OECD countries, and the majority of these countries are represented in the included studies.

Arguably, interest in “radicalization” only began in 2005, and the popularity of the radicalization paradigm has grown since this time (Neumann & Kleinmann, 2013). Similarly, terrorism research as a broad field only began to really take off from this period as well (Schuurman, 2020). The studies included in this review span the breadth of this period and are consistent with the findings from overviews of the literature with respect to the uptick in research in recent years.

Given the specific substantive and methodological inclusion/exclusion criteria of this review, we believe that the included studies represent the complete body of work that meets these criteria. Despite this, there are some important acknowledgments that need to be made. First, a number of additional factors were identified for which we were only able to identify a single effect size, thereby precluding their inclusion in the meta‐analysis. This means that there are still additional factors that will need to be synthesized in future studies. Second, we also acknowledge that there are measures of radicalization/extremism that are used by researchers which fall outside of this review's inclusion criteria. While these studies may provide important contributions to the broader body of knowledge, in a review of this scope it was important to ensure a high degree of similarity in the outcome measures that would be included. Lastly, the lack of longitudinal data means that it is difficult to refer to the factors examined in this study as predictors, or risk and protective factors. For this reason, we have chosen to use the more accurate classification of putative factors (see below Section 7.1.1).

### 7.3 Quality of the evidence

#### 7.3.1 Putative risk or protective factors

In order for a factor to be classified as a “risk factor,” in addition to demonstrating a predictive quality of the outcome of interest, it also must be shown to have temporally preceded the displaying of the outcome (Kraemer et al., 1997). Similarly, in order for a factor to be classified as a risk based “protective factor,” it must predict a lower probability of the outcome of interest and temporally precede the time at which the outcome is measured (Lösel & Farrington, 2012). It therefore seems to be the case that only single‐sample longitudinal studies could establish the temporal ordering of factors that is needed to confirm their status as risk or protective factors (Murray et al., 2009).

As such, when dealing with cross‐sectional data, it may only be possible to categorize factors as 'putative risk/protective factors', which are factors that have been found to correlate with the outcome of interest in the theorized direction but for which temporal ordering cannot be established (Kraemer et al., 1997, 2001). Such a classification is prevalent in psychology (e.g., May & Klonsky, 2016), criminology (Assink et al., 2019), and radicalization research (Bhui, Hicks, et al., 2012; Lloyd & Dean, 2015; Monahan, 2012). This classification is quite useful since empirically supported putative factors can be used to inform evidence‐based policy in the absence of stronger evidence.

Given the above, the factors described in this review would best be classified as “putative risk/protective factors.” However, temporal ordering can still sometimes be assumed for some factors derived from cross‐sectional and case‐control research, enabling them to meet the standards for classification as risk or protective factors (Jacobi et al., 2004). For example, this review included experiential factors that occur during adolescence, such as parental abuse or being bullied. The ages at which these self‐reported events occurred are highly likely to have preceded the development of radical attitudes or intentions. And they certainly preceded the engagement in radical behaviors, especially terrorism offending. Additionally, certain psychological and personality related factors can still be classified as risk or protective factors since they are innate characteristics. For example, while a debate exists as to the extent to which levels of self‐control are innate, most agree that at a minimum, individuals have an innate predisposition to differential levels of self‐control. Similarly, while traits such as authoritarianism and fundamentalism, and (anti) democratic beliefs can certainly be changed through learning, to a large degree they are deeply rooted in the individual's innate personality (Adorno et al., 1950). Similarly, other personality characteristics such as those associated with the Big Five, are to a large degree innate.

Beyond this, when dealing with risk factors derived from these types of observational studies, arguably the most important quality criteria pertain to measures of the dependent and independent variables, appropriate sampling, and appropriate comparisons made statistically (Murray et al., 2009). The studies included in this review generally employed validated or otherwise appropriate measures for both independent and outcome variables. They also, for the most part, employed acceptable sampling procedures. Studies also employed appropriate statistical techniques for identifying the strength of the relationship between independent and outcome variables (Table S1). In this regard, another important consideration is whether the factors have a theoretically plausible relationship with the outcome (Murray et al., 2009). Indeed, the majority of factors analysed in this study, especially those with the largest estimates (as per the above discussion), are theoretically derived factors which have plausible relationships with radicalization outcomes.

### 7.4 Limitations and potential biases in the review process

#### 7.4.1 Limitations of the results

Correlations are in and of themselves insufficient for drawing causal inferences (e.g., Einhorn & Hogarth, 1986; White, 1990). In this context, cross‐sectional studies, made up the majority of studies included in this review, and they can rarely make claims to temporal ordering. In addition, correlations from such studies are open to alternative explanations and may be sensitive to changes in other factors (confounding) (Murray et al., 2009). For these reasons, an analysis such as the one presented in the current review must be taken with some degree of caution. Whereas correlational evidence may be quite sufficient for applications to risk assessment, its ability to inform the development of interventions is more limited (Murray et al., 2009). Nevertheless, given the dearth of evaluation studies in counter‐radicalization research (Gielen, 2017; Koehler, 2019), the evidence provided by this review can still serve as a first step in the identification of prospective risk factors and causal mechanisms, and can still serve to inform the selection of factors for targeting by interventions (Derzon, 2010; Kraemer et al., 2005, Murray et al., 2009). By arranging risk and protective factors according to their relative magnitude in rank‐order, and employing a tier based system for their categorization as having small, moderate and large relationships (Cohen, 1988), the review has identified the degree to which the factors differ in their relationship with radicalization outcomes.

#### 7.4.2 Review process

We acknowledge that there are studies that may provide important evidence concerning the risk and protective factors for radicalization that were not included in this review on account of their outcome measures failing to meet the review's inclusion criteria. For example, some studies are known to assess 'willingness to die for a cause/group'. However, as described above, such studies were excluded since a willingness to die does not necessarily indicate a willingness to use violence against others (e.g., Bélanger et al., 2014). Nevertheless, we do not believe that the review's results are biased as a result of having excluded such studies. Rather it means that our results are based on a more homogenous set of outcomes and should therefore be considered to be more robust from a meta‐analysis perspective.

Moreover, while we encourage replication, we acknowledge that the authors' knowledge of the existing literature may have impacted the number of studies that passed through the different screening stages. That is, in the initial screening stages, studies whose titles and abstracts may have appeared to research assistants to be suitable for progressing to the subsequent stage may have been familiar to the main authors and known to them to not meet eligibility criteria. As such, while we are confident that a replication of this study will reach the same or similar results, differences in the number of studies included or excluded at different stages would be expected.

### 7.5 Agreements and disagreements with other studies or reviews

#### 7.5.1 Comparing with the initial review

Our preliminary review included 57 studies published between 2006 and 2018 (Wolfowicz et al., 2019). The current review identified five new studies that were published during this period that had not been captured by the earlier review. Overall there is general consistency of the results.

Some of the main differences between the earlier review and the current review can be found in the makeup of the included studies. Among the studies published between 2018 and 2020 are a large number of studies examining outcomes related to Radical intentions, and a number of studies examining Left‐wing ideological strains of radicalism. Based primarily from these new studies, the current review was able to examine 20 new factors that did not appear in the previous review, including: Factors such as those derived from the Big Five (Agreeableness, Conscientiousness, Neuroticism, Openness, Extraversion), the Dark Triad set of personality traits, Harmonious and Obsessive Passion, and Machoism.

**Figure 12:**
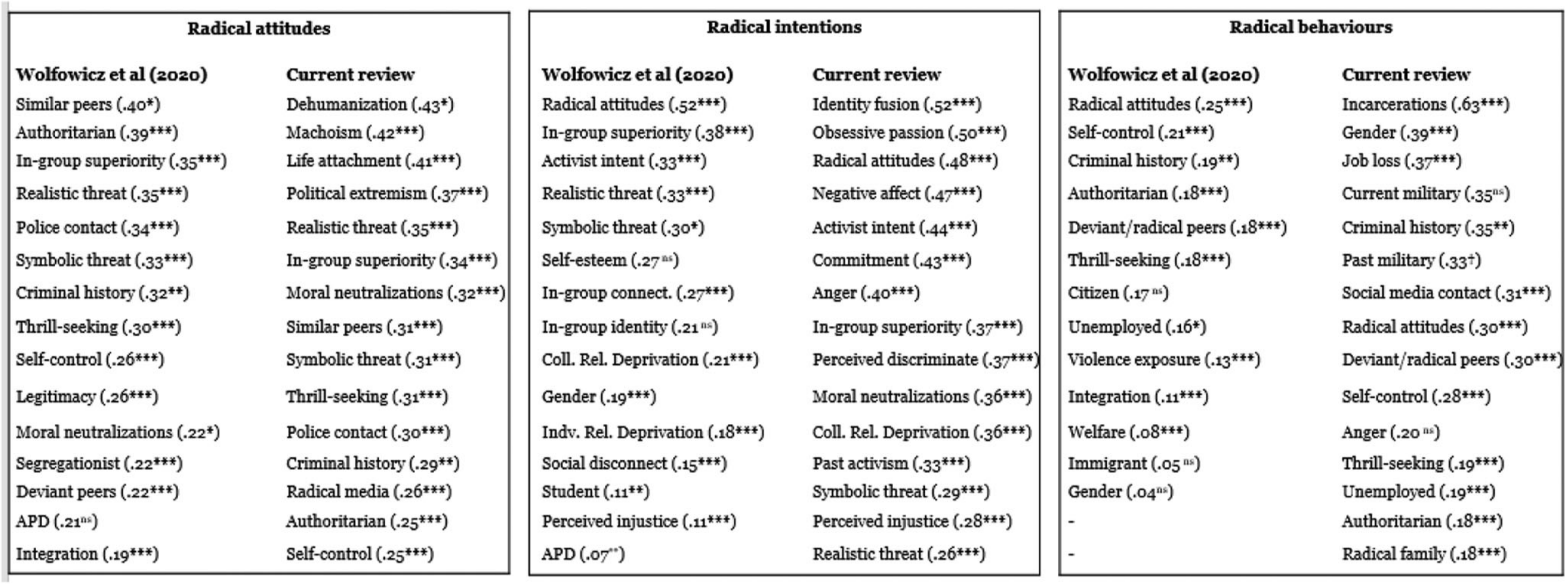
Juxtaposition of the top 15 risk factors in the preliminary and current reviews

The preliminary review found that traditional criminogenic and criminotrophic factors consistently ranked as the factors with the largest effect sizes. The results of the current review are generally consistent with these earlier findings. However, there are some important differences in the rank orders as a result of the addition of new factors, and some differences in the size of the estimates as a result of new effect sizes being added to the analyses. The below figure (Figure 4) juxtaposes the top 15 risk factors for each of the three outcomes between the preliminary review and the current one. The differences and overlap highlighted here are representative of the degree of divergence and convergence across the full rank orders. Of note, the preliminary review only included 13 risk factors for radical behaviors, whereas the current review included 26 (Figure 12).

## 8 AUTHORS' CONCLUSIONS

### 8.1 Implications for practice and policy

#### 8.1.1 Risk assessment

A key tool in the counter‐radicalization strategies of democratic countries is risk assessment. Risk assessment is currently being carried out by a range of actors, including education, health care, and law enforcement professionals. Unfortunately, many of the current risk assessment tools in use are not evidence‐based (Scarcella et al., 2016). Many tools have been adapted from risk assessment tools for other forms of violent cognitions and behaviors. And while there may be significant overlap in the risk and protective factors for radicalization, and other deviant outcomes—as demonstrated by this review—there may also be important differences. With the identification of a broad set of risk and protective factors for radicalization, this review can help to contribute to the development of radicalization‐specific risk assessment tools (King et al., 2018).

Another issue that current risk assessment tools suffer from is that they overwhelmingly use a nominal scaling approach (Klausen et al., 2016; Silke, 2014). There are two issues with this approach. First, a nominal approach hinders the ability to differentiate between different levels of risk. Second, as a result of this failure, low‐risk individuals may be grouped together with high‐risk individuals, which could lead to infringements of rights and increase stigmatization, both of which can have unwanted backlash effects (Scarcella et al., 2016).

Third, when used in the context of targeting dynamic factors in order to reduce the risk of future radical behavior, as in the context of the Risk‐Needs‐Responsivity approach, a poor identification of specific needs is likely to lead to unsuccessful intervention outcomes (Dean, 2016; Mullins, 2009). The results of this review may provide a first‐step in the move towards more sophisticated approaches to risk assessment.

#### 8.1.2 Counter‐radicalization initiatives

Counter‐radicalization initiatives in democratic countries generally seek to target underlying risk and protective factors that are believed to reduce the likelihood of radical attitudes (Dandurand, 2014). However, the lack of a solid evidentiary base upon which to select factors for targeting “has made way for programming that has either been overly broad or inappropriately targeted, resulting in ineffectiveness, or an exacerbation of existing tensions” (Harper, 2018, p. 23). Given the type of correlational data analysed in this study, this review serves as but a first step to the identification of those factors which, if targeted in the context of interventions, may lead to better intervention outcomes (Derzon, 2010; Murray et al., 2009).

Whether explicit or not, the basic assumption underpinning many counter‐radicalization strategies is that in reducing the prevalence of “radicalization” in the population, there will be a spill‐over effect in a reduction in the risk of terrorism. Most policies seek to accomplish this by the targeting of underlying risk and protective factors. The findings of this review that Radical attitudes do have a significant relationship with Radical behaviors would appear to support the approach and the identification of a broad range of risk and protective factors for radical attitudes can serve as a basis for the selection of the types of factors that could be targeted in the context of counter‐radicalization interventions. Given that many of the factors with the most salient relationships with radicalization outcomes are traditional criminogenic factors, the findings suggest that the field of counter‐radicalization may be able to draw on existing evidence from criminology (LaFree & Miller, 2008; LaFree & Freilich, 2019; Hasisi et al., 2020).

### 8.2 Implications for research

#### 8.2.1 Theoretical implications

The results of this review demonstrate the relevance of traditional criminological frameworks to the study of risk factors for radicalization. As noted above, factors associated with Social Learning Theory, Social Control/Bonds Theory and Self‐Control Theory, were all found to have modest relationships with the outcomes examined. While Social learning and control theories appear well suited to explanations of radicalization, given their focus on socialization, Hirschi and Gottfredson (2001) previously suggested that self‐control would be unrelated to radicalization. Nevertheless, the current review summarizes what appears to be a growing body of evidence that indeed, self‐control and related factors are salient risk factors for radicalization (Rottweiler et al., 2020). With a growing body of criminological research into other radicalization related phenomena, the results are encouraging for the relevance of criminological theories for the identification of important risk and protective factors (LaFree et al., 2020).

At the same time, the results of the review highlight the importance of factors associated with key social‐psychological frameworks. Based on the range of psychological factors identified in this review, it would appear that Horgan's (2016) “call to arms” for more psychological research on radicalization has been heeded. As per the above discussion, we believe that the findings that psychological and personality related factors tend to cluster at the upper tiers of the rank orders with criminogenic factors provides justification for calls to integrate criminological and psychological approaches in the study of radicalization (Rice, 2009).

#### 8.2.2 Reconciling debates

The radicalization literature is full of debates concerning the relevance of a range of factors. To date, the different sides to these debates are able to rally anecdotal evidence to defend their opposing positions. Various and multiple case‐studies will often be cited in order to provide support for a given position, or to discredit the opposing view. Even in relying on empirical evidence, one could find studies that demonstrate both positive and negative relationships between factors such as Socio‐economic status and Education with radicalization outcomes. Indeed, in our analysis we had effect sizes for both of these factors which pointed in opposing directions. One of the advantages of a systematic review and meta‐analysis such as the current study is that it can help to reconcile such inconsistencies (Pratt & Cullen, 2005).

One of the ongoing debates in the literature surrounds the role of factors such as integration and societal connectedness. These factors often take a central role in counter radicalization strategies, which view poor integration, and alienation as significant risk factors for radicalization. In criticizing the overall approaches taken by many countries, some researchers have questioned, or even outright dismissed the role of integration related factors. They point to cases of seemingly well integrated individuals who have carried out acts of terrorism as evidence that integration may not be so important to radicalization outcomes (Pisoiu, 2012; Rahimi & Graumans, 2015). This review found that for radical attitudes, Social disconnectedness and Low integration fell exactly in the middle of the rank‐order. The estimate for Low integration on Radical intentions was only marginally smaller than it was for Radical attitudes, and it was found to have a small but significant effect on self‐reported Radical violence as well. As such, whilst perhaps not the most important—as assessed by effect size—of all factors, integration is relevant to radicalization outcomes.

Another key debate concerns the role of religion. While it is evident that many radical ideologies are steeped in religious doctrines, some believe that religion and religious practices per se are not risk factors for radicalization. These critics often point to the fact that the overwhelming majority of religious adherents are not radical at all. It may also be pointed out that many terrorists, were not overly religious, or otherwise lacked a strong religious knowledge. For example, many Islamist terrorists are known to have engaged in practices forbidden by their religion, such as the consumption of alcohol and drugs (Dawson, 2018). But religion and religiosity can be broken down in to multiple components, and as we found in this review, these different components have vastly different relative effects on radicalization. First, the estimate for Religiosity as it pertains to radical attitudes was found to be small and not statistically significant. However, the estimate for frequency of attendance at places of worship had a very small yet statistically significant effect. Only two factors in the rank order separated this factor from In‐group identity, which included studies measuring identification with one's religious group, which had a slightly larger yet statistically significant estimate. Additionally, the estimate for prayer frequency was more modest, and was situated close to the middle of the rank order. Importantly, the estimate for authoritarianism/fundamentalism had the 14th largest estimate, placing it as a relatively moderate estimate, and the estimate for in‐group superiority had the 5th largest effect size,. For radical intentions, it was found that being a religious convert had a modest effect. Additionally, the size of the estimate for in‐group identity was almost double the size as it was for radical attitudes, and In‐group superiority ranked as the 10th largest estimate. However, when it came to Radical behaviors, the estimates for both Religious upbringing and religious convert were the smallest of all factors analysed and were also not statistically significant. Nevertheless, the estimate for Authoritarianism/Fundamentalism had a robust relationship with self‐reported radical behaviors. As such, while not necessarily the most important factors, it is clear that some elements pertaining to religion, including ways of thinking and identity, are salient risk factors for radicalization more generally (Dawson, 2018).

These examples serve to demonstrate the importance of grounding our understandings about risk and protective factors in quantitative data. They also serve to demonstrate that it is important for research to identify subelements from different risk factor domains in order to identity the relative importance of different factors. Researchers should avoid hasty dismissals of factors as being relevant for radicalization before they have been thoroughly and systematically investigated. In this regard, it is clear that more research as needed.

#### 8.2.3 The importance of longitudinal research

As discussed above, this review was based primarily off of cross‐sectional studies. This means that with few exceptions, the factors described in this review can only be classified as putative factors. However, this should not be seen to much as a limitation of the review as much as it is a reflection of the state of the literature. As described above, only a small number of longitudinal studies were identified that met the inclusion criteria for the review. But the relatively recent publication of these studies indicates that there is an emerging shift towards longitudinal study in this field. This shift could possibly be aided and reinforced by the findings of this review. Researchers working in the field could build on the salient putative factors identified in this review to inform, and prioritize the types of factors assessed in costlier longitudinal research (Jacobi et al., 2004; Kraemer et al., 1997; Rutter, 2005).

## ROLES AND RESPONSIBILITIES

- *Content*: Michael Wolfowicz, Yael Litmanovitz, David Weisburd, and Badi Hasisi.
- *Systematic review methods*: Michael Wolfowicz, Yael Litmanovitz, and David Weisburd.
- *Statistical analysis*: Michael Wolfowicz, Yael Litmanovitz, David Weisburd, and Badi Hasisi.
- *Information retrieval*: Michael Wolfowicz and Yael Litmanovitz.

## SOURCES OF SUPPORT

This study was supported by the DHS Science and Technology Directorate and the Five Research and Development (5RD) Countering Violent Extremism Network.

This study also received support by the European Commission's Horizon 2020 programme, Grant no. 699824.

While the authors have been involved in the development of related research, no studies published by the authors are included in this review. The authors previously published the preliminary results of part of this study elsewhere.

## PLANS FOR UPDATING THE REVIEW

The main review author intends to update the systematic review every 5 years.

## DIFFERENCES BETWEEN PROTOCOL AND REVIEW

While the review itself did not deviate from the protocol, as described above there were some revisions made to the risk of bias items. The extraction tool reflects these changes which were made in order to for the items to be more informative given the nature of the data.

## DATA AND ANALYSES

### Searches

#### GESIS

Radikalismus OR Terrorismus OR Extremismus

#### ISI

AB=(Australia OR Czech OR Greece OR Japan OR Netherlands OR Slovenia OR Austria OR Denmark OR Hungary OR Korea OR New Zealand OR Spain OR Belgium OR Estonia OR Iceland OR Latvia OR Norway OR Sweden OR Canada OR Finland OR Ireland OR Lithuania OR Poland OR Switzerland OR Chile OR France OR Israel OR Luxemburg OR Portugal OR UK OR Columbia OR Germany OR Italy OR Mexico OR Slovakia OR USA OR America OR Democra* OR Europe OR EU OR OECD OR West* OR high income) AND AB=(Radical* OR recruit* OR extrem* OR politically motivated OR foreign fighter* OR Terror* OR Lone wol* OR homegrown OR homegrown OR sympath* OR support OR ORJustif* OR facilitate OR engage* OR activis* OR collective) AND AB=(Jihad* OR Islam* OR Salaf* OR rightwing OR neoNazi OR farright OR nationalist OR whitesupremacist OR RWE OR left wing OR extreme left OR anarchist OR LWE OR singleissue OR ethn* OR separatis*) AND AB=(Risk* OR factor* OR predict* OR propensity OR likelihood OR predispos* OR vulnerab* OR causal OR putative OR determinant OR Root OR correlat*) OR TI=(Australia OR Czech OR Greece OR Japan OR Netherlands OR Slovenia OR Austria OR Denmark OR Hungary OR Korea OR New Zealand OR Spain OR Belgium OR Estonia OR Iceland OR Latvia OR Norway OR Sweden OR Canada OR Finland OR Ireland OR Lithuania OR Poland OR Switzerland OR Chile OR France OR Israel OR Luxemburg OR Portugal OR UK OR Columbia OR Germany OR Italy OR Mexico OR Slovakia OR USA OR America OR Democra* OR Europe OR EU OR OECD OR West* OR high income) AND TI=(Radical* OR recruit* OR extrem* OR politically motivated OR foreign fighter* OR Terror* OR Lone wol* OR homegrown OR homegrown OR sympath* OR support OR ORJustif* OR facilitate OR engage* OR activis* OR collective) AND TI=(Jihad* OR Islam* OR Salaf* OR rightwing OR neoNazi OR farright OR nationalist OR whitesupremacist OR RWE OR left wing OR extreme left OR anarchist OR LWE OR singleissue OR ethn* OR separatis*) AND TI=(Risk* OR factor* OR predict* OR propensity OR likelihood OR predispos* OR vulnerab* OR causal OR putative OR determinant OR Root OR correlat*)

#### Ebsco (Criminal Justice Abstracts, ERIC, OpenDissertations, Political Science Complete, Social Work Abstracts, SocINDEX with Full Text, Violence & Abuse Abstracts)

AB (=(Australia OR Czech OR Greece OR Japan OR Netherlands OR Slovenia OR Austria OR Denmark OR Hungary OR Korea OR New Zealand OR Spain OR Belgium OR Estonia OR Iceland OR Latvia OR Norway OR Sweden OR Canada OR Finland OR Ireland OR Lithuania OR Poland OR Switzerland OR Chile OR France OR Israel OR Luxemburg OR Portugal OR UK OR Columbia OR Germany OR Italy OR Mexico OR Slovakia OR USA OR America OR Democra* OR Europe OR EU OR OECD OR West* OR high income) AND AB (Radical* OR recruit* OR extrem* OR politically motivated OR foreign fighter* OR Terror* OR Lone wol* OR homegrown OR homegrown OR sympath* OR support OR ORJustif* OR facilitate OR engage* OR activis* OR collective) AND AB (Jihad* OR Islam* OR Salaf* OR rightwing OR neoNazi OR farright OR nationalist OR whitesupremacist OR RWE OR left wing OR extreme left OR anarchist OR LWE OR singleissue OR ethn* OR separatis*) AND AB (Risk* OR factor* OR predict* OR propensity OR likelihood OR predispos* OR vulnerab* OR causal OR putative OR determinant OR Root OR correlat*)

#### Psychinfo

Abstract: Radical* *OR* Abstract: recruit* *OR* Abstract: extrem* *OR* Abstract: politically motivated *OR* Abstract: foreign fighter* *OR* Abstract: Terror* *OR* Abstract: Lone wol* *OR* Abstract: homegrown *OR* Abstract: homegrown *OR* Abstract: sympath* *OR* Abstract: support *OR* Abstract: ORJustif* *OR* Abstract: facilitate *OR* Abstract: engage* *OR* Abstract: activis* *OR* Abstract: collective *AND* Abstract: Risk* *OR* Abstract: factor* *OR* Abstract: predict* *OR* Abstract: propensity *OR* Abstract: likelihood *OR* Abstract: predispose* *OR* Abstract: predisposition *OR* Abstract: vulnerab* *OR* Abstract: causal *OR* Abstract: putative *OR* Abstract: determinant *OR* Abstract: Root *OR* Abstract: correlate* *AND* Abstract: Australia *OR* Abstract: Czech *OR* Abstract: Greece *OR* Abstract: Japan *OR* Abstract: Netherlands *OR* Abstract: Slovenia *OR* Abstract: Austria *OR* Abstract: Denmark *OR* Abstract: Hungary *OR* Abstract: Korea *OR* Abstract: New Zealand *OR* Abstract: Spain *OR* Abstract: Belgium *OR* Abstract: Estonia *OR* Abstract: Iceland *OR* Abstract: Latvia *OR* Abstract: Norway *OR* Abstract: Sweden *OR* Abstract: Canada *OR* Abstract: Finland *OR* Abstract: Ireland *OR* Abstract: Lithuania *OR* Abstract: Poland *OR* Abstract: Switzerland *OR* Abstract: Chile *OR* Abstract: France *OR* Abstract: Israel *OR* Abstract: Luxemburg *OR* Abstract: Portugal *OR* Abstract: UK *OR* Abstract: Columbia *OR* Abstract: Germany *OR* Abstract: Italy *OR* Abstract: Mexico *OR* Abstract: Slovakia *OR* Abstract: USA *OR* Abstract: America *OR* Abstract: Democra* *OR* Abstract: Europe *OR* Abstract: EU *OR* Abstract: OECD *OR* Abstract: West* *OR* Abstract: high income *AND* Abstract: Jihad* *OR* Abstract: Islam* *OR* Abstract: Salaf* *OR* Abstract: rightwing *OR* Abstract: neoNazi *OR* Abstract: farright *OR* Abstract: nationalist *OR* Abstract: whitesupremacist *OR* Abstract: RWE *OR* Abstract: left wing *OR* Abstract: extreme left *OR* Abstract: anarchist *OR* Abstract: LWE *OR* Abstract: singleissue *OR* Abstract: ethn* *OR* Abstract: separatis*

#### Pubmed

(((Australia[Title/Abstract] OR Czech[Title/Abstract] OR Greece[Title/Abstract] OR Japan[Title/Abstract] OR Netherlands[Title/Abstract] OR Slovenia[Title/Abstract] OR Austria[Title/Abstract] OR Denmark[Title/Abstract] OR Hungary[Title/Abstract] OR Korea[Title/Abstract] OR New Zealand[Title/Abstract] OR Spain[Title/Abstract] OR Belgium[Title/Abstract] OR Estonia[Title/Abstract] OR Iceland[Title/Abstract] OR Latvia[Title/Abstract] OR Norway[Title/Abstract] OR Sweden[Title/Abstract] OR Canada[Title/Abstract] OR Finland[Title/Abstract] OR Ireland[Title/Abstract] OR Lithuania[Title/Abstract] OR Poland[Title/Abstract] OR Switzerland[Title/Abstract] OR Chile[Title/Abstract] OR France[Title/Abstract] OR Israel[Title/Abstract] OR Luxemburg[Title/Abstract] OR Portugal[Title/Abstract] OR UK[Title/Abstract] OR Columbia[Title/Abstract] OR Germany[Title/Abstract] OR Italy[Title/Abstract] OR Mexico[Title/Abstract] OR Slovakia[Title/Abstract] OR USA[Title/Abstract] OR America[Title/Abstract] OR Democra*[Title/Abstract] OR Europe[Title/Abstract] OR EU[Title/Abstract] OR OECD[Title/Abstract] OR West*[Title/Abstract] OR high income[Title/Abstract]) AND (Radical*[Title/Abstract] OR recruit*[Title/Abstract] OR extrem*[Title/Abstract] OR politically motivated[Title/Abstract] OR foreign fighter*[Title/Abstract] OR Terror*[Title/Abstract] OR Lone wol*[Title/Abstract] OR homegrown[Title/Abstract] OR homegrown[Title/Abstract] OR sympath*[Title/Abstract] OR support[Title/Abstract] OR ORJustif*[Title/Abstract] OR facilitate[Title/Abstract] OR engage*[Title/Abstract] OR activis*[Title/Abstract] OR collective[Title/Abstract])) AND ((Jihad*[Title/Abstract] OR Islam*[Title/Abstract] OR Salaf*[Title/Abstract] OR rightwing[Title/Abstract] OR neoNazi[Title/Abstract] OR farright[Title/Abstract] OR nationalist[Title/Abstract] OR whitesupremacist[Title/Abstract] OR RWE[Title/Abstract] OR left wing[Title/Abstract] OR extreme left[Title/Abstract] OR anarchist[Title/Abstract] OR LWE[Title/Abstract] OR singleissue[Title/Abstract] OR ethn*[Title/Abstract] OR separatis*[Title/Abstract])) AND (Risk*[Title/Abstract] OR factor*[Title/Abstract] OR predict*[Title/Abstract] OR propensity[Title/Abstract] OR likelihood[Title/Abstract] OR predispos*[Title/Abstract] OR vulnerab*[Title/Abstract] OR causal[Title/Abstract] OR putative[Title/Abstract] OR determinant[Title/Abstract] OR Root[Title/Abstract] OR correlat*[Title/Abstract])

#### SocialCare Online

[‐ AbstractOmitNorms:'Australia OR Czech OR Greece OR Japan OR Netherlands OR Slovenia OR Austria OR Denmark OR Hungary OR Korea OR New Zealand OR Spain OR Belgium OR Estonia OR Iceland OR Latvia OR Norway OR Sweden OR Canada OR Finland OR Ireland OR Lithuania OR Poland OR Switzerland OR Chile OR France OR Israel OR Luxemburg OR Portugal OR UK OR Columbia OR Germany OR Italy OR Mexico OR Slovakia OR USA OR America OR Democra* OR Europe OR EU OR OECD OR West* OR high income'

‐ AND AbstractOmitNorms:'Radical* OR recruit* OR extrem* OR politically motivated OR foreign fighter* OR Terror* OR Lone wol* OR homegrown OR homegrown OR sympath* OR support OR ORJustif* OR facilitate OR engage* OR activis* OR collective OR Jihad* OR Islam* OR Salaf* OR rightwing OR neoNazi OR farright OR nationalist OR whitesupremacist OR RWE OR left wing OR extreme left OR anarchist OR LWE OR singleissue OR ethn* OR separatis*'

‐ AND AbstractOmitNorms:'Risk* OR factor* OR predict* OR propensity OR likelihood OR predispos* OR vulnerab* OR causal OR putative OR determinant OR Root OR correlat*']

## Supporting information

Supporting information

Supporting information

## Footnotes

1 We note that there are other approaches to categorizing correlation sizes (e.g., Hemphill, 2003).
