# Supplementary material for: Cognitive and behavioral radicalization: A systematic review of the putative risk and protective factors
Source: Campbell Syst Rev. 2021 Jul 20;17(3):e1174. doi: 10.1002/cl2.1174 (PMC10121227; doi:10.1002/cl2.1174)
Supplement: Supplementary file 1 — Supporting information [file CL2-17-e1174-s002.docx]

**Extraction and coding sheet**

|  | **Category** | **Data type** | **Selection/Data entry** | **Comments/ Notes** |  |  |  |
| --- | --- | --- | --- | --- | --- | --- | --- |
| **Publication information** |  |  |  |  |  |  |  |
|  | 1. Author(s) name(s) | Text |  |  |  |  |  |
|  | 2. Title | Text |  |  |  |  |  |
|  | 3. Year | Number |  |  |  |  |  |
|  | 4. Full reference | Text |  |  |  |  |  |
|  |  |  |  |  |  |  |  |
|  | Type: | Categorical |  |  |  |  |  |
|  | a. Journal article (peer reviewed) |  |  |  |  |  |  |
|  | b. Book |  |  |  |  |  |  |
|  | c. Government report |  |  |  |  |  |  |
|  | e. Police report |  |  |  |  |  |  |
|  | f. Technical report |  |  |  |  |  |  |
|  | g. Conference paper |  |  |  |  |  |  |
|  | h. Dissertation or thesis |  |  |  |  |  |  |
|  | J. Other (specify) |  |  |  |  |  |  |
|  |  |  |  |  |  |  |  |
|  | **Publication details (complete for each study reported)** |  |  |  |  |  |  |
|  | 5. Location/country | Text |  |  |  |  |  |
|  | 6. Language | Text |  |  |  |  |  |
|  | 7. Date(s) of research | Number |  |  |  |  |  |
|  | a. Start: | Date |  |  |  |  |  |
|  | b. Finish: | Date |  |  |  |  |  |
|  | 8. Source of funding | Categorical |  |  |  |  |  |
|  | a. Government |  |  |  |  |  |  |
|  | b. Foreign government |  |  |  |  |  |  |
|  | c. Local university/research body |  |  |  |  |  |  |
|  | d. Foreign university/research body |  |  |  |  |  |  |
|  | e. NGO |  |  |  |  |  |  |
|  | f. Other |  |  |  |  |  |  |
|  | 9. Bodies involved (tick all applicable) |  |  |  |  |  |  |
|  | a. University/research agency |  |  |  |  |  |  |
|  | b. Health Service |  |  |  |  |  |  |
|  | c. Police/ Justice system |  |  |  |  |  |  |
|  | d. Other government departments |  |  |  |  |  |  |
|  | e. Other |  |  |  |  |  |  |
|  |  |  |  |  |  |  |  |
|  |  |  |  |  |  |  |  |
|  | 10. Evaluated by | Text |  |  |  |  |  |
|  | 11. Conflict context (Y/N)? | Y/N |  |  |  |  |  |
|  | 12. Ethical issues (Y/N. If yes, describe)? | Y/N |  |  |  |  |  |
|  |  |  |  |  |  |  |  |
| **Methodology** |  |  |  |  |  |  |  |
|  | 13. Type of study: | Categorical |  |  |  |  |  |
|  | a. Longitudinal |  |  |  |  |  |  |
|  | b. Cross-sectional |  |  |  |  |  |  |
|  | c. Case control |  |  |  |  |  |  |
|  |  |  |  |  |  |  |  |
|  |  |  |  |  |  |  |  |
|  | 14. Radicalization outcome: | Categorical |  |  |  |  |  |
|  | a. Radical attitudes  b. Radical intentions  c. Radical behaviors |  |  |  |  |  |  |
|  |  |  |  |  |  |  |  |
|  | 14a. Measure of radical attitudes: | Categorical |  |  |  |  |  |
|  | a. Support/justification of radical violence  b. Support/justification of terrorism  c. Support/justification of specific radical event(s)  d. Other  14b. Measure of radical intentions:  a. Willingness/intentions towards carrying out of radical violence  b. Willingness/intentions towards carrying out of terrorism  14c. Measure of radical behaviors:  a. Convicted of radical offences  b. Convicted of terrorism offences  c. Self-reported involvement in radical behaviors |  |  |  |  |  |  |
|  |  |  |  |  |  |  |  |
|  | 15. Source of measure: | Categorical |  |  |  |  |  |
|  | a. Open-source |  |  |  |  |  |  |
|  | b. Official data (e.g. police) |  |  |  |  |  |  |
|  | c. Self-reported |  |  |  |  |  |  |
|  | d. Peer/Family-reported |  |  |  |  |  |  |
|  | e. Practitioner-reported |  |  |  |  |  |  |
|  | f. Other |  |  |  |  |  |  |
|  |  |  |  |  |  |  |  |
|  | 16. Term/s used by author to describe radicalization: | Text |  |  |  |  |  |
|  | a. Radical(s)/radicalization/radicalism |  |  |  |  |  |  |
|  | b. Terrorist(s) |  |  |  |  |  |  |
|  | c. Extremist(s)/violent extremist(s) |  |  |  |  |  |  |
|  | d. Islamist/Jihadist/Right-wing/Left-wing extremist |  |  |  |  |  |  |
|  | e. Other |  |  |  |  |  |  |
|  |  |  |  |  |  |  |  |
|  | 17. Author definition of radicalization: | Text |  |  |  |  |  |
|  |  |  |  |  |  |  |  |
|  | 18.Sample size |  |  |  |  |  |  |
|  | a. Total sample size | Numerical |  |  |  |  |  |
|  | b. Sample size of comparison group | Numerical |  |  |  |  |  |
|  |  |  |  |  |  |  |  |
|  | 19. Was attrition a problem? | Y/N |  |  |  |  |  |
|  | a. Yes (describe) |  |  |  |  |  |  |
|  | b. No |  |  |  |  |  |  |
|  | c. Not applicable |  |  |  |  |  |  |
|  |  |  |  |  |  |  |  |
|  | 20. Initial response rate | Numerical |  |  |  |  |  |
|  |  |  |  |  |  |  |  |
|  | 21. Sample age (mean) | Numerical |  |  |  |  |  |
|  |  |  |  |  |  |  |  |
|  | 22. Sample gender | Categorical |  |  |  |  |  |
|  | a. Male |  |  |  |  |  |  |
|  | b. Female |  |  |  |  |  |  |
|  | c. Mixed |  |  |  |  |  |  |
|  |  |  |  |  |  |  |  |
|  | 23. Sample socio-economic status | Categorical |  |  |  |  |  |
|  | a. Low |  |  |  |  |  |  |
|  | b. Average |  |  |  |  |  |  |
|  | c. High |  |  |  |  |  |  |
|  | d. Mixed |  |  |  |  |  |  |
|  | e. Other |  |  |  |  |  |  |
|  |  |  |  |  |  |  |  |
| **Risk of bias** |  |  |  |  |  |  |  |
|  | 24. Study population description. Does the document describe the source | Y/N |  |  |  |  |  |
|  | population in replicable detail? |  |  |  |  |  |  |
|  | a. Yes |  |  |  |  |  |  |
|  | b. No |  |  |  |  |  |  |
|  | c. Unclear |  |  |  |  |  |  |
|  |  |  |  |  |  |  |  |
|  | 25. Study population criteria: Does the document list all inclusion and exclusion criteria for participation? | Y/N |  |  |  |  |  |
|  | a. Yes |  |  |  |  |  |  |
|  | b. No |  |  |  |  |  |  |
|  | c. Unclear |  |  |  |  |  |  |
|  |  |  |  |  |  |  |  |
|  | 26. Outcome validity: Was the outcome measured using a validated measure? | Y/N |  |  |  |  |  |
|  | a. Yes |  |  |  |  |  |  |
|  | b. No |  |  |  |  |  |  |
|  | c. Unclear |  |  |  |  |  |  |
|  |  |  |  |  |  |  |  |
|  |  |  |  |  |  |  |  |
|  | 27. Risk factor validity: Were all measures of the risk factor based on a validated | Y/N |  |  |  |  |  |
|  | measure? |  |  |  |  |  |  |
|  | a. Yes |  |  |  |  |  |  |
|  | b. No |  |  |  |  |  |  |
|  | c. Unclear |  |  |  |  |  |  |
|  |  |  |  |  |  |  |  |
|  | 28. Selective risk factor reporting: was the study free from reporting bias? | Y/N |  |  |  |  |  |
|  | a. Yes |  |  |  |  |  |  |
|  | b. No |  |  |  |  |  |  |
|  | c. Unclear |  |  |  |  |  |  |
|  |  |  |  |  |  |  |  |
|  | 29. Selective analysis reporting: was the study free from analysis reporting bias? | Y/N |  |  |  |  |  |
|  | a. Yes |  |  |  |  |  |  |
|  | b. No |  |  |  |  |  |  |
|  | c. Unclear |  |  |  |  |  |  |
|  |  |  |  |  |  |  |  |
|  | 30. Is there evidence that the study's sample overlaps with that of another included study?  a. Yes  b. No  c. Suspected overlap |  |  |  |  |  |  |
|  |  |  |  |  |  |  |  |
|  | 31. Did the study report its sampling method?  a. Yes  b. No  c. Sampling method can be deduced from description  31a. If yes (to Q30), what is the sampling method? |  |  |  |  |  |  |
|  |  |  |  |  |  |  |  |
| **Risk factors (Complete** | | | | |  |  |  |
| **for each factor reported)** | 35. Risk factor | Text |  |  |  |  |  |
|  | 36. Conceptual definition of risk factor | Text |  |  |  |  |  |
|  | 37.Operational definition | Text |  |  |  |  |  |
|  | 38. Origin of the factor's variable: | Categorical |  |  |  |  |  |
|  | a. Official data (government/police) |  |  |  |  |  |  |
|  | b. Self-reported |  |  |  |  |  |  |
|  | c. Peer-reported |  |  |  |  |  |  |
|  | d. Family-reported |  |  |  |  |  |  |
|  | e. Practitioner-reported (including school) |  |  |  |  |  |  |
|  | f. Other |  |  |  |  |  |  |
|  |  |  |  |  |  |  |  |
|  | 39. Measured retrospectively? | Y/N |  |  |  |  |  |
|  | a. Yes |  |  |  |  |  |  |
|  | b. No |  |  |  |  |  |  |
|  | c. Unclear |  |  |  |  |  |  |
|  |  |  |  |  |  |  |  |
|  | 40. Time-invariant risk factor? (if the study design is not longitudinal and the | Y/N |  |  |  |  |  |
|  | factor is not time-invariant, the factor will be classified as a correlate) |  |  |  |  |  |  |
|  | a. Yes |  |  |  |  |  |  |
|  | b. No |  |  |  |  |  |  |
|  | c. Unclear |  |  |  |  |  |  |
|  |  |  |  |  |  |  |  |
|  |  |  |  |  |  |  |  |
|  | 41. Risk factor domain: | Categorical |  |  |  |  |  |
|  | a. Socio-demographic |  |  |  |  |  |  |
|  | b. Psychological/Personality |  |  |  |  |  |  |
|  | c. Experiential |  |  |  |  |  |  |
|  | d. Attitudinal |  |  |  |  |  |  |
|  | e. Criminogenic |  |  |  |  |  |  |
|  | f. Other |  |  |  |  |  |  |
|  |  |  |  |  |  |  |  |
|  | 42. Did a test of statistical significance indicate statistically significant | Categorical |  |  |  |  |  |
|  | differences between the comparison and radical group(s)? |  |  |  |  |  |  |
|  | a. Yes |  |  |  |  |  |  |
|  | b. No |  |  |  |  |  |  |
|  | c. Can’t tell |  |  |  |  |  |  |
|  | d. N/A (no testing completed) |  |  |  |  |  |  |
|  |  |  |  |  |  |  |  |
|  | 43. Was a standardized effect size reported? | Y/N |  |  |  |  |  |
|  | a. Yes |  |  |  |  |  |  |
|  | b. No |  |  |  |  |  |  |
|  | If Yes: |  |  |  |  |  |  |
|  | 44. Effect size measure  a. *r*  b. *b*  c. B(exp)  d. OR  e. Other | Categorical |  |  |  |  |  |
|  |  |  |  |  |  |  |  |
|  | 45. Effect size | Numerical |  |  |  |  |  |
|  | 46. Standard error of effect size | Numerical |  |  |  |  |  |
|  | 47. Effect size reported on page number | Numerical |  |  |  |  |  |
|  |  |  |  |  |  |  |  |
|  | 48. If no to Q43, are data available to calculate effect size? | Y/N |  |  |  |  |  |
|  | d. Yes |  |  |  |  |  |  |
|  | e. No |  |  |  |  |  |  |
|  |  |  |  |  |  |  |  |
|  | 49. If yes to Q48, type of data effect size can be calculated from: | Categorical |  |  |  |  |  |
|  | a. Means and standard deviations |  |  |  |  |  |  |
|  | b. Frequencies or proportions (dichotomous) |  |  |  |  |  |  |
|  | c. Frequencies or proportions (polychotomous) |  |  |  |  |  |  |
|  | d. Unadjusted correlation coefficient |  |  |  |  |  |  |
|  | e. Multiple regression coefficients (unstandardized) |  |  |  |  |  |  |
|  | f. Multiple regression coefficients (standardized) |  |  |  |  |  |  |
|  | g. Odds ratio(s) |  |  |  |  |  |  |
|  | h. t-value or F-value |  |  |  |  |  |  |
|  | i. Chi-square (df=1) |  |  |  |  |  |  |
|  | j.. Other (specify) |  |  |  |  |  |  |
|  |  |  |  |  |  |  |  |
|  | Select relevant as per response to Q49:  Means and Standard Deviations |  |  |  |  |  |  |
|  | 50. Radicalized/Radicalizing group mean | Numerical |  |  |  |  |  |
|  | 51.Comparison group mean | Numerical |  |  |  |  |  |
|  | 52.Radicalized/Radicalizing group standard deviation | Numerical |  |  |  |  |  |
|  | 53. Comparison group standard deviation | Numerical |  |  |  |  |  |
|  |  |  |  |  |  |  |  |
|  | Proportions or frequencies |  |  |  |  |  |  |
|  | 54. n of radicalized/radicalizing group with the risk factor(s) | Numerical |  |  |  |  |  |
|  | 55.n of comparison group with the risk factor(s) | Numerical |  |  |  |  |  |
|  | 56. Proportion of radicalized/radicalizing group with the risk factor | Numerical |  |  |  |  |  |
|  | 57.Proportion of comparison group with the risk factor | Numerical |  |  |  |  |  |
|  |  |  |  |  |  |  |  |
|  | Regression coefficients and correlations |  |  |  |  |  |  |
|  | 58.Unadjusted correlation coefficient | Numerical |  |  |  |  |  |
|  | 59.Standardized regression coefficient | Numerical |  |  |  |  |  |
|  | 60.Unstandardized regression coefficient | Numerical |  |  |  |  |  |
|  | 61.Standard deviation of predictor | Numerical |  |  |  |  |  |
|  | 62.Control variables | Numerical |  |  |  |  |  |
|  |  |  |  |  |  |  |  |
|  | Significance Tests |  |  |  |  |  |  |
|  | 63.t-value | Numerical |  |  |  |  |  |
|  | 64.F-value | Numerical |  |  |  |  |  |
|  | 65.Chi-square value (df=1) | Numerical |  |  |  |  |  |
|  |  |  |  |  |  |  |  |
|  | Calculated Effect Size |  |  |  |  |  |  |
|  | 67.Effect size | Numerical |  |  |  |  |  |
|  | 68.Standard error of effect size | Numerical |  |  |  |  |  |
|  |  |  |  |  |  |  |  |
| **Authors’ conclusion** | | | | |  |  |  |
|  | 70. What did the authors conclude about the relationship? | Categorical/Text |  |  |  |  |  |
|  | a. Risk factors increases likelihood of radicalization and/or recruitment |  |  |  |  |  |  |
|  | b. Risk factor reduces the likelihood of radicalization and/or recruitment |  |  |  |  |  |  |
|  | c. No effect of risk factor on radicalization and/or recruitment |  |  |  |  |  |  |
|  | d. Unclear/no conclusion stated by authors |  |  |  |  |  |  |
