# Supplementary material for: Cognitive and behavioral radicalization: A systematic review of the putative risk and protective factors
Source: Campbell Syst Rev. 2021 Jul 20;17(3):e1174. doi: 10.1002/cl2.1174 (PMC10121227; doi:10.1002/cl2.1174)
Supplement: Supplementary file 2 — Supporting information [file CL2-17-e1174-s001.docx]

# Online supplements

Table 13: Risk of bias assessment

| **Study** | **Sample age** | **Sample gender** | **Sample country** | **Other characteristics** | **Inclusion criteria** | **Sampling method** | **Validated outcome** | **Validated instruments** | **Overlap** | **NS findings** | **Reported all** | **Supplementary material** |
| --- | --- | --- | --- | --- | --- | --- | --- | --- | --- | --- | --- | --- |
| Abdi (2019) | Y | Y | Y | Y | Y | Snowball | Y | Y | Y | N | Y | N |
| Acevedo & Chaudhary(2015) | Y | Y | Y | Y | Y | Random | N | N | Y | Y | N | N |
| Adam-Troian et al (2020) | - | - | - | - | - | - | - | - | - | - | - | - |
| Study 1 | Y | Y | Y | N | N | Random | Y | Y | N | N | N | N |
| Study 2 | Y | Y | Y | N | N | Random | Y | Y | N | N | N | N |
| Study 3 | Y | Y | Y | N | N | Random | Y | Y | N | N | N | N |
| Ahearn et al (2020) | Y | Y | Y | Y | Y | Proportional quota | Y | Y | Y | Y | Y | N |
| Allington et al (2019) | Y | Y | Y | Y | Y | Representative random | Y | N | N | Y | N | N |
| Altunbas & Thornton (2011) | Y | Y | Y | Y | Y | Random | Y | N | Y | Y | Y | N |
| Baier et al (2010) | N | N | Y | Y | Y | Purposive random | N | Y | N | Y | Y | N |
| Baier et al (2016) | - | - | - | - | - | - | - | - | - | - | - | - |
| Right-wing sample | N | N | Y | Y | Y | Purposive random | Y | Y | N | Y | Y | N |
| Left-wing sample | N | N | Y | Y | Y | Purposive random | Y | Y | N | Y | Y | N |
| Islamist sample | N | N | Y | Y | Y | Purposive random | Y | Y | N | Y | Y | N |
| Becker (2019) | Y | Y | Y | Y | Y | Representative random | Y | Y | Y | Y | N | N |
| Becker (2020) | Y | Y | Y | Y | Y | Random | Y | Y | N | Y | Y | N |
| Bélanger et al (2014) | Y | Y | Y | Y | Y | Random | N | Y | N | N | N | N |
| Bélanger et al (2019a) |  |  |  |  |  |  |  |  |  |  |  |  |
| Study 1 | Y | Y | Y | Y | Y | Purposive random | N | Y | N | Y | N | Y |
| Study 2 | Y | Y | Y | Y | Y | Purposive random | N | Y | N | Y | N | Y |
| Study 3 | N | Y | Y | Y | Y | Purposive random | N | Y | N | Y | N | Y |
| Study 4 | Y | Y | Y | Y | Y | Purposive random | N | Y | N | Y | N | Y |
| Study 5 | Y | Y | Y | Y | Y | Purposive random | N | Y | N | Y | N | Y |
| Bélanger et al (2019b) | - | - | - | - | - | - | - | - | - | - | - | - |
| Study 1 | Y | Y | Y | Y | N | Random | Y | Y | N | Y | N | N |
| Study 2 | Y | Y | Y | Y | N | Random | Y | Y | N | Y | N | N |
| Study 3 | Y | Y | Y | Y | N | Random | Y | Y | N | Y | N | N |
| Beller & Kröger (2020) |  |  |  |  |  |  |  |  |  |  |  |  |
| Study 1 | Y | Y | Y | Y | Y | Random | N | N | Y | Y | N | Y |
| Study 2 | N | Y | Y | Y | Y | Random | N | N | N | Y | N | Y |
| Berger (2016) |  |  |  |  |  |  |  |  |  |  |  |  |
| UK sample | Y | Y | Y | N | N | Random | N | N | Y | Y | N | Y |
| France sample | Y | Y | Y | N | N | Random | N | N | Y | Y | N | Y |
| Germany sample | Y | Y | Y | N | N | Random | N | N | Y | Y | N | Y |
| Spain sample | Y | Y | Y | N | N | Random | N | N | Y | Y | N | Y |
| Berrebi (2007) | Y | Y | Y | Y | Y | Representative | Y | Y | N | Y | Y | N |
| Besta et al (2015) | Y | Y | Y | N | Y | Random | N | Y | N | Y | Y | N |
| Bhui et al (2014) | Y | Y | Y | Y | Y | Proportional quota | Y | Y | Y | Y | Y | Y |
| Bhui et al (2016) | Y | Y | Y | Y | Y | Proportional quota | Y | Y | Y | Y | Y | Y |
| Bhui et al (2019) | N | Y | Y | Y | Y | Quota | Y | Y | N | Y | Y | Y |
| Brettfeld & Wetzels (2007) | Y | Y | Y | Y | Y | Purposive random | N | Y | N | Y | Y | N |
| Capelos & Demertzis (2018) | N | N | Y | N | N | Representative | N | N | N | Y | N | Y |
| Cardeli et al (2020) | Y | Y | Y | Y | Y | Snowball | Y | Y | Y | Y | Y | Y |
| Charkawi et al (2020) | N | Y | Y | Y | Y | Random | N | N | N | Y | N | N |
| Cherney & Murphy (2017) | Y | Y | Y | Y | Y | Representative | N | N | N | Y | Y | N |
| Clemmow et al (2020) | Y | Y | Y | Y | Y | Random | Y | Y | N | Y | Y | Y |
| Coid et al (2016) | Y | Y | Y | Y | Y | Quota | Y | Y | N | Y | N | Y |
| Dahl (2019) | Y | Y | Y | Y | Y | Representative | N | N | N | Y | N | N |
| De Waele & (2016) | N | Y | Y | Y | Y | Random | N | Y | Y | Y | N | N |
| Decker & Pyrooz (2018) | Y | Y | Y | Y | Y | Disproportionate stratified random | Y | Y | N | Y | Y | N |
| Delia Deckard & Jacobson (2015) | Y | Y | Y | Y | Y | Stratafied random | N | N | N | Y | Y | N |
| Doosje et al (2012) | Y | Y | Y | N | N | Random | N | Y | Maybe | Y | N | N |
| Doosje et al (2013) | Y | Y | Y | Y | N | Random | N | Y | Maybe | Y | N | N |
| Egger & Magni-Berton (2019) | Y | Y | Y | Y | Y | Random | N | N | Y | Y | N | N |
| Ellis et al (2015) | Y | Y | Y | Y | Y | Snowball | Y | Y | Y | Y | N | N |
| Ellis et al (2016) | Y | Y | Y | Y | Y | Snowball | Y | Y | Y | Y | Y | N |
| Ellis et al (2019) | Y | Y | Y | Y | Y | Snowball | Y | Y | Y | Y | Y | N |
| Faragó et al (2019) | N | N | Y | N | N | Representative | N | N | N | Y | Y | N |
| Feddes et al (2015) | Y | Y | Y | Y | Y | Purposive | N | Y | N | Y | Y | N |
| Fodeman et al (2020) | N | Y | Y | Y | Y | Quota | Y | Y | N | Y | Y | N |
| Frissen (2019) | Y | Y | Y | Y | N | Disproportional stratified | Y | Y | N | Y | Y | N |
| Frissen et al (2019) | Y | Y | Y | Y | N | Disproportional stratified | Y | Y | Maybe | Y | Y | N |
| Frounfelker et al (2019) | Y | Y | Y | Y | Y | Disproportional stratified | Y | Y | N | Y | Y | N |
| Goede et al (2019) | Y | Y | Y | Y | Y | Purposive random | Y | Y | N | Y | Y | N |
| Gøtzsche-Astrup (2019a) |  |  |  |  |  |  |  |  |  |  |  |  |
| Study 1 | Y | Y | Y | Y | N | Probability | Y | Y | N | Y | Y | Y |
| Study 2 | Y | Y | Y | Y | N | Probability | Y | Y | N | Y | Y | Y |
| Gøtzsche-Astrup (2019b) | N | N |  |  |  |  |  |  |  |  |  |  |
| Study 1 | " | " | Y | N | N | Representative | Y | Y | N | Y | N | N |
| Study 2 | " | " | Y | N | N | Representative | Y | Y | N | Y | N | N |
| Study 3 | " | " | Y | N | N | Representative | Y | Y | N | Y | N | N |
| Gøtzsche-Astrup (2020) |  |  |  |  |  |  |  |  |  |  |  |  |
| Sample 1 | Y | Y | Y | Y | N | Quota | Y | Y | N | Y | N | Y |
| Sample 2 | Y | Y | Y | Y | N | Quota | Y | Y | N | Y | N | Y |
| Sample 3 | Y | Y | Y | Y | N | Quota | Y | Y | N | Y | N | Y |
| Gousse-Lessard et al (2013) |  |  |  |  |  |  |  |  |  |  |  |  |
| Study 1 | Y | Y | Y | Y | Y | N/R | Y | Y | N | Y | N | N |
| Study 2 | Y | Y | Y | Y | Y | Purposive | N | Y | N | Y | N | N |
| Study 3 | Y | Y | Y | Y | Y | Purposive | N | Y | N | Y | N | N |
| Groppi (2018) | Y | Y | Y | Y | Y | Convenience | N | N | N | Y | Y | N |
| Harms (2017) | Y | Y | Y | Y | Y | Representative | Y | Y | N | Y | Y | N |
| Jackson et al (2013) | Y | N | Y | Y | Y | Quota | N | Y | N | Y | Y | Y |
| Jahnke et al (2020) |  |  |  |  |  |  |  |  |  |  |  |  |
| Study 1 | Y | Y | Y | Y | Y | Convenience | Y | Y | N | Y | Y | Y |
| Study 2 | Y | Y | Y | Y | Y | Convenience | Y | Y | N | Y | Y | Y |
| Jasko et al (2016) | Y | Y | Y | Y | Y | Representative random | Y | Y | Y | Y | N | N |
| Jensen et al (2016) | Y | Y | Y | Y | Y | Representative random | Y | Y | Y | Y | Y | N |
| Jones et al (2020) |  |  |  |  |  |  |  |  |  |  |  |  |
| Study 1 | Y | Y | Y | Y | Y | Convenience | N | Y | N | Y | N | N |
| Study 2 | Y | Y | Y | Y | Y | Purposive | Y | Y | N | Y | N | N |
| Kalmoe (2014) |  |  |  |  |  |  |  |  |  |  |  |  |
| Study 1 | N | N | Y | N | N | Random | N | Y | N | Y | N | Y |
| Study 2 | N | N | Y | N | N | Random | N | Y | N | Y | N | Y |
| Study 3 | Y | Y | Y | N | N | Convenience | N | Y | N | Y | N | N |
| Kerodal et al (2016) | N | N | Y | Y | Y | Representative | Y | Y | N | Y | N | N |
| Krueger (2008) | Y | Y | Y | Y | Y | Random | Y | Y | Y | Y | N | N |
| Kunst et al (2018) |  |  |  |  |  |  |  |  |  |  |  |  |
| Study 1 | Y | Y | Y | Y | Y | Purposive | Y | Y | N | Y | N | Y |
| Study 2 | Y | Y | Y | Y | Y | Purposive | Y | Y | N | Y | N | Y |
| Study 3 | Y | Y | Y | Y | Y | Purposive | Y | Y | N | Y | N | Y |
| LaFree et al (2018) | Y | Y | Y | Y | Y | Representative random | Y | Y | Y | Y | N | N |
| LaRue (2012) | N | N | Y | Y | Y | Random | N | N | Y | Y | N | N |
| Lemieux & Asal (2010) | Y | Y | Y | Y | Y | Random | N | Y | N | Y | Y | N |
| Littler (2017) | Y | Y | Y | Y | Y | Random | N | N | Y | Y | N | N |
| Ljujic et al (2020) | Y | Y | Y | Y | Y | Random | Y | Y | N | Y | Y | N |
| Lobato et al (2018) |  |  |  |  |  | Convenience | Y | Y | N |  |  |  |
| Study 1 | Y | Y | Y | Y | Y |  |  |  |  | Y | N | N |
| Study 2 | Y | Y | Y | Y | Y | N/R | Y | Y | N | Y | N | N |
| Study 3 | Y | Y | Y | Y | Y | N/R | Y | Y | N | Y | N | N |
| Study 4 | Y | Y | Y | Y | Y | Random | Y | Y | N | Y | N | N |
| Lobato et al (2020) | Y | Y | Y | Y | N | Random | Y | Y | N | Y | N | Y |
| Loughery (2018) | Y | Y | Y | Y | Y | Convenience | Y | Y | N | Y | N | N |
| Lyons (2015) |  |  |  |  |  |  |  |  |  |  |  |  |
| Study 1 | Y | Y | Y | Y | Y | Purposive | N | Y | N | Y | N | N |
| Study 2 | Y | Y | Y | Y | Y | Purposive | N | Y | N | Y | N | N |
| Study 3 | Y | Y | Y | Y | Y | Purposive | N | Y | N | Y | N | N |
| Macdougall et al (2018) | Y | Y | Y | Y | N | Random | N | Y | Maybe | Y | N | N |
| Mahfud & Adam-Troian (2019) |  |  |  |  |  |  |  |  |  |  |  |  |
| Study 1 | Y | Y | Y | Y | Y | Random | Y | Y | N | Y | Y | Y |
| Study 2 | Y | Y | Y | Y | Y | Random | Y | Y | N | Y | Y | Y |
| Manzoni et al (2019) | Y | Y |  |  |  |  |  |  |  |  |  |  |
| Right-wing sample | N | N | Y | Y | Y | Purposive random | Y | Y | N | Y | Y | N |
| Left-wing sample | N | N | Y | Y | Y | Purposive random | Y | Y | N | Y | Y | N |
| Islamist sample | N | N | Y | Y | Y | Purposive random | Y | Y | N | Y | Y | N |
| McCauley (2012) | Y | Y | Y | Y | N | Random | N | N | Y | Y | N | N |
| Miconi et al (2019) | N | Y | Y | Y | Y | Purposive | Y | Y | Y | Y | Y | N |
| Moskalenko & McCauley (2009) |  |  |  |  |  |  |  |  |  |  |  |  |
| Study1 | Y | Y | Y | Y | N | Convenience | Y | Y | N | N | N | N |
| Study2 | Y | Y | Y | Y | N | Representative | Y | Y | N | N | N | N |
| Moyano (2011) | Y | Y |  |  |  |  |  |  |  |  |  |  |
| Sample 1 | N | N | Y | Y | N | Accidental | Y | Y | N | Y | Y | N |
| Sample 2 | N | N | Y | Y | N | Accidental | Y | Y | N | Y | Y | N |
| Moyano & Trujillo (2014) | Y | Y |  |  |  |  |  |  |  |  |  |  |
| Sample 1 | N | Y | Y | Y | N | N/R | Y | Y | N | Y | N | N |
| Sample 2 | N | Y | Y | Y | N | N/R | Y | Y | N | Y | N | N |
| Narraina (2013) | Y | Y | Y | Y | Y | Representative | N | N | Y | Y | N | N |
| Nivette et al (2017) | Y | Y | Y | Y | Y | Stratafied random | N | Y | N | Y | N | N |
| Obaidi et al (2018a) |  |  |  |  |  |  |  |  |  |  |  |  |
| Study 1 | N | Y | Y | Y | N | Purposive | N | Y | N | Y | N | N |
| Study 2 | N | Y | Y | Y | N | Snowball | N | Y | N | Y | N | N |
| Obaidi et al (2018b) |  |  |  |  |  |  |  |  |  |  |  |  |
| Study 1 | N | Y | Y | Y | Y | Random | N | Y | N | Y | N | Y |
| Study 2 | N | Y | Y | Y | Y | Random | N | Y | Y | Y | N | Y |
| Obaidi et al (2018c) |  |  |  |  |  |  |  |  |  |  |  |  |
| Study 1 | N | Y | Y | Y | Y | Purposive | N | Y | Y | Y | N | Y |
| Study 2 (Sample 1) | Y | Y | Y | Y | Y | Purposive | N | Y | N | Y | N | Y |
| Study 2 (Sample 2) | Y | Y | Y | Y | Y | Purposive | N | Y | N | Y | N | Y |
| Obaidi et al (2019) |  |  |  |  |  |  |  |  |  |  |  |  |
| Study 1 | N | Y | Y | Y | N | Purposive | N | Y | N | Y | N | Y |
| Study 2 | N | Y | Y | Y | N | Purposive | N | Y | N | Y | N | Y |
| Study 3 | N | Y | Y | Y | N | Purposive | N | Y | N | Y | N | Y |
| Study 4 | N | Y | Y | Y | N | Purposive | N | Y | N | Y | N | Y |
| Study 5 | N | Y | Y | Y | N | Purposive | N | Y | N | Y | N | Y |
| Study 6 | N | Y | Y | Y | N | Snowball | N | Y | N | Y | N | Y |
| Obaidi (2020) | Y | Y | Y | Y | Y | Convenience/Snowball | N | Y | N | Y | N | Y |
| Obaidi et al (2020) |  |  |  |  |  |  |  |  |  |  |  |  |
| Study 1 | N | Y | Y | N | N | N/R | N | Y | N | Y | Y | N |
| Study 2 | Y | Y | Y | N | N | N/R | N | Y | N | Y | Y | N |
| Study 3 | Y | Y | Y | N | N | N/R | N | Y | N | Y | Y | N |
| Okan (2017) | N | Y | Y | N | N | Random | N | Y | N | Y | N | N |
| Oskooii & Dana (2017) | Y | Y | Y | Y | Y | Random | N | N | N | Y | N | Y |
| Ozer (2020) | Y | Y | Y | Y | Y | Purposive non-random | Y | Y | Y | Y | N | Y |
| Ozer et al (2019) |  |  |  |  |  |  |  |  |  |  |  |  |
| Sample 1 | Y | Y | Y | Y | N | Purposive non-random | Y | Y | N | Y | N | Y |
| Sample 2 | Y | Y | Y | Y | N | Purposive non-random | Y | Y | Y | Y | N | Y |
| Ozer & Bertelsen (2018) |  |  |  |  |  |  |  |  |  |  |  |  |
| Sample 1 | Y | Y | Y | N | N | Random | Y | Y | Y | Y | N | N |
| Sample 2 | Y | Y | Y | N | N | Random | Y | Y | Y | Y | N | N |
| Ozer & Bertelsen (2019) |  |  |  |  |  |  |  |  |  |  |  |  |
| Sample 1 | Y | Y | Y | N | N | Random | Y | Y | Y | Y | N | N |
| Sample 2 | Y | Y | Y | N | N | Random | Y | Y | Y | Y | N | N |
| Ozer & Bertelsen (2020) | Y | Y | Y | N | N | Random | Y | Y | Y | Y | N | N |
| Pauwels & Heylen (2017) | N | Y | Y | Y | Y | Random | N | Y | Y | Y | N | N |
| Pauwels & De Waele (2014) | N | Y | Y | Y | N | Random | N | Y | Y | Y | N | N |
| Pauwels & Schils (2016) | Y | Y | Y | Y | N | Random | N | Y | Y | Y | Y | N |
| Pauwels & Boudry (2017) | N | Y | Y | Y | N | Random | N | Y | Y | Y | N | N |
| Pedersen et al (2017) | Y | Y | Y | Y | Y | Representative | N | N | Y | Y | Y | N |
| Perliger et al (2016) | N | Y | Y | Y | Y | Convenience | Y | Y | N | Y | Y | N |
| Pfundmair et al (2019) | Y | Y | Y | Y | Y | Representative | Y | Y | N | N | Y | Y |
| Rip et al (2012) | Y | Y |  |  |  |  |  |  |  |  |  |  |
| Study 1 | Y | Y | Y | Y | Y | Purposive | N | Y | N | Y | N | N |
| Study 2 | Y | Y | Y | Y | Y | Purposive | N | Y | N | Y | N | N |
| Rousseau et al (2016) | Y | Y | Y | Y | Y | Convenience | Y | Y | Y | Y | Y | N |
| Rousseau et al (2019a) | N | Y | Y | Y | Y | Purposive | Y | Y | Y | Y | Y | Y |
| Rousseau et al (2019b) | N | Y | Y | Y | Y | Purposive | Y | Y | Y | Y | Y | N |
| Rousseau et al (2020) | Y | Y |  |  |  |  |  |  |  |  |  |  |
| Sample 1 | N | N | Y | Y | Y | Purposive | Y | Y | Y | Y | Y | N |
| Sample 2 | N | N | Y | Y | Y | Purposive | Y | Y | Y | Y | Y | N |
| Schbley & McCauley (2005) | N | N | Y | Y | Y | Convenience | N | N | N | N | N | N |
| Schmuck & Tribastone (2020) | Y | Y | Y | Y | Y | Purposive | N | Y | N | N | N | Y |
| Schumpe et al (2018) |  |  |  |  |  |  |  |  |  |  |  |  |
| Study 1 | Y | Y |  |  |  | Purposive | N | Y | N | Y | N | N |
| Study 2 | Y | Y |  |  |  | Purposive | N | Y | N | Y | N | N |
| Schumpe et al (2020) |  |  |  |  |  |  |  |  |  |  |  |  |
| Study 1 | Y | Y | Y | N | N | Random | Y | Y | N | Y | Y | N |
| Study 2 | Y | Y | Y | N | N | Random | Y | Y | N | Y | Y | N |
| Study 3 | Y | Y | N | N | N | Random | Y | Y | N | Y | Y | N |
| Study 4 | Y | Y | N | N | N | Random | Y | Y | N | Y | Y | N |
| Study 5 | Y | Y | N | N | Y | Purposive | N | Y | N | Y | Y | N |
| Study 6 | Y | Y | N | N | Y | Purposive | N | Y | N | Y | Y | N |
| Study 7 | Y | Y | N | N | Y | Purposive | N | Y | N | Y | Y | N |
| Study 8 | Y | Y | N | N | Y | Purposive | N | Y | N | Y | Y | N |
| Study 9 | Y | Y | N | N | Y | Purposive | N | Y | N | Y | Y | N |
| Simon et al (2013) | Y | Y | Y | Y | Y | Convenience | N | Y | N | Y | Y | N |
| Stankov et al (2010a) | Y | Y | Y | Y | N | N/R | Y | Y | N | Y | N | N |
| Stankov et al (2010b) | Y | Y | Y | Y | N | N/R | Y | Y | N | Y | N | N |
| Storm et al (2020) |  |  |  |  |  |  |  |  |  |  |  |  |
| Study 1 | Y | Y | Y | Y | Y | Representative | N | N | Y | Y | N | N |
| Study 2 | Y | Y | Y | Y | Y | Representative | N | N | Y | Y | N | N |
| Study 3 | Y | Y | Y | Y | Y | Purposive random | N | N | N | Y | N | N |
| Study 4 | Y | Y | Y | Y | Y | Representative | N | N | Y | Y | N | N |
| Szlachter et al (2012) | Y | Y | Y | Y | N | Purposive random | N | Y | N | Y | N | N |
| Tahir et al (2019) |  |  |  |  |  |  |  |  |  |  |  |  |
| Study 1 | Y | Y | Y | Y | N | Purposive random | N | Y | N | Y | Y | Y |
| Study 2 | Y | Y | Y | Y | N | Purposive random | N | Y | N | Y | Y | Y |
| Tausch et al (2009) | Y | Y | Y | Y | Y | Purposive random | N | N | N | Y | Y | N |
| Tausch et al (2011) | Y | Y | Y | Y | Y | Purposive random | N | N | N | Y | Y | N |
| Travaglino & Moon (2020) |  |  | - | - | - | - | - | - | - | - | - | - |
| Study 1 | Y | Y | Y | Y | N | Convenience | Y | Y | N | Y | Y | Y |
| Study 2 | Y | Y | Y | Y | N | Convenience | Y | Y | N | Y | Y | Y |
| Study 3 | Y | Y | Y | Y | N | Convenience | Y | Y | N | Y | Y | Y |
| Study 4 | Y | Y | Y | Y | N | Convenience | Y | Y | N | Y | Y | Y |
| Study 5 | Y | Y | Y | Y | N | Convenience | Y | Y | N | Y | Y | Y |
| Troian et al (2019) |  |  | - | - | - | - | - | - | - | - | - | - |
| Study 1 | Y | Y | Y | Y | N | Purposive | Y | Y | N | Y | N | N |
| Study 2 | Y | Y | Y | Y | N | Random | Y | Y | N | Y | N | N |
| Study 3 | Y | Y | Y | Y | N | Random | Y | Y | N | Y | N | N |
| Trujillo et al (2016) |  |  | - | - | - | - | - | - | - | - | - | - |
| Study1 | Y | Y | Y | Y | N | Accidental | Y | Y | N | Y | Y | N |
| Study2 | Y | Y | Y | Y | N | Accidental | Y | Y | N | Y | Y | N |
| van Bergen et al (2015) |  |  |  |  |  |  |  |  |  |  |  |  |
| Sample1 | Y | Y | Y | Y | Y | Purposive random | N | Y | Maybe | N | N | N |
| Sample2 | Y | Y | Y | Y | Y | Purposive random | N | Y | Y | N | N | N |
| van Bergen et al (2016) | Y | Y | Y | Y | Y | Purposive random | N | Y | Y | Y | N | N |
| Van den Bos et al (2009) | Y | Y | - | - | - | - | - | - | - | - | - | - |
| Islamist model | N | N | Y | Y | Y | Random | N | Y | Maybe | Y | Y | N |
| Right-wing model | N | N | Y | Y | Y | Random | N | Y | Maybe | Y | Y | N |
| Left-wing model | N | N | Y | Y | Y | Random | N | Y | Maybe | Y | Y | N |
| van der Veen (2016) |  |  | - | - | - | - | - | - | - | - | - | - |
| Study1 | Y | Y | Y | Y | N | Random | N | Y | N | Y | Y | Y |
| Study2 | Y | Y | Y | Y | N | Random | N | Y | Maybe | Y | Y | Y |
| Vergani et al (2019) | Y | Y | Y | Y | Y | Random | Y | Y | N | N | N | Y |
| Victoroff et al (2012) |  |  | - | - | - | - | - | - | - | - | - | - |
| EU model | Y | Y | Y | Y | Y | Random | N | N | Y | Y | N | N |
| US model | Y | Y | Y | Y | Y | Random | N | N | Y | Y | N | N |
| Wojcieszak (2010) | Y | Y | Y | Y | Y | Purposive random | N | N | N | Y | Y | N |
| Zaidise et al (2007) | N | Y | Y | Y | Y | Random | N | N | N | Y | Y | N |
| Zhirkov et al (2014) | Y | Y | Y | Y | Y | Random | N | N | Y | Y | N | N |
